# Magnetron-Sputtered Lead Titanate Thin Films for Pyroelectric Applications: Part 2—Electrical Characteristics and Characterization Methods

**DOI:** 10.3390/ma17030589

**Published:** 2024-01-25

**Authors:** Morteza Fathipour, Yanan Xu, Mukti Rana

**Affiliations:** 1 Division of Physics, Engineering, Mathematics and Computer Sciences & Optical Science Center for Applied Research, Delaware State University, Dover, DE 19901, USA; m.fathipour33@gmail.com; 2 Division of Physics, Engineering, Mathematics and Computer Sciences, Delaware State University, Dover, DE 19901, USA; yxu@desu.edu

**Keywords:** lead titanate, PCT, PZT, PLZT, PT, pyroelectric materials, ferroelectric material, ferroelectricity, pyroelectricity, electrical characterization methods

## Abstract

Pyroelectric materials are naturally electrically polarized and exhibits a built-in spontaneous polarization in their unit cell structure even in the absence of any externally applied electric field. These materials are regarded as one of the ideal detector elements for infrared applications because they have a fast response time and uniform sensitivity at room temperature across all wavelengths. Crystals of the perovskite lead titanate (PbTi$O_{3}$) family show pyroelectric characteristics and undergo structural phase transitions. They have a high Curie temperature (the temperature at which the material changes from the ferroelectric (polar) to the paraelectric (nonpolar) phase), high pyroelectric coefficient, high spontaneous polarization, low dielectric constant, and constitute important component materials not only useful for infrared detection, but also with vast applications in electronic, optic, and MEMS devices. However, the preparation of large perfect and pure single crystals PbTi$O_{3}$ is challenging. Additionally, difficulties arise in the application of such bulk crystals in terms of connection to processing circuits, large size, and high voltages required for their operation. In this part of the review paper, we explain the electrical behavior and characterization techniques commonly utilized to unravel the pyroelectric properties of lead titanate and its derivatives. Further, it explains how the material preparation techniques affect the electrical characteristics of resulting thin films. It also provides an in-depth discussion of the measurement of pyroelectric coefficients using different techniques.

## 1. Introduction

The electrical characteristics and characterization methods of magnetron-sputtered lead titanate thin films play a crucial role in understanding and optimizing their performance for pyroelectric applications. In this second part of the review article, we focus on providing a comprehensive insight into the advances made in the electrical characterization of these materials. A key aspect of this exploration is the systematic study of the conduction mechanism within lead titanate thin films. It is essential to explore the conduction mechanism as it can provide information about phenomena such as contact effects, the physical nature of the interface, and the bulk space charges that often dominate the transport mechanisms in these materials. By delving into the conduction mechanisms, researchers can gain invaluable information about the intricate interplay of charges and the material’s electrical properties. Understanding the conduction mechanisms becomes particularly crucial when addressing device-reliability issues. Imprint, for instance, refers to the preference of a ferroelectric (FE) capacitor for one polarization state over the other. Investigating the conduction mechanisms aids in deciphering the factors influencing imprint and finding ways to mitigate its effects, thereby enhancing the stability and reliability of devices. Fatigue is another important reliability concern, involving the loss of polarization due to bipolar cycling of the capacitor. By studying the conduction mechanisms, researchers can identify the underlying causes of fatigue and develop strategies to improve the endurance of lead titanate thin films in practical applications. Polarization relaxation, characterized by the momentary lag in the dielectric constant of a material, is also a significant aspect addressed in the electrical characterization. This phenomenon is typically caused by the delay in molecular polarization concerning a changing electric field in a dielectric medium. Understanding the conduction mechanisms aids in elucidating the factors contributing to polarization relaxation, enabling researchers to design materials with improved response times. Resistance degradation and breakdown are additional challenges that demand a thorough exploration of the electrical characteristics. It is essential to identify the mechanisms leading to resistance degradation and breakdown to ensure the longevity and robustness of devices based on magnetron-sputtered lead titanate thin films. In conclusion, the systematic study of the conduction mechanisms in lead titanate thin films offers crucial insights into various electrical phenomena and device-reliability issues. This knowledge is instrumental in advancing the understanding and optimization of these materials for pyroelectric applications, ultimately paving the way for enhanced performance and reliability in practical devices [1].

## 2. Measurement of the Electrical Characteristics

Methods developed for the electrical characterization of FE materials such as current-voltage, I(V), current-time, I(t), capacitance-voltage $C\left(V\right),$ dielectric constant- electric field D(E), and polarization-electric field P(E) are also compatible with the characterization of the pyroelectrics. However, for pyroelectric materials, the measurement of the pyroelectric coefficient, p, is also extremely important. In this part, we discuss the abovementioned development and measurement methods and identify the strongest stress on thin-film pyroelectric materials. In this section, we delve further into the specific electrical characterization methods and processes relevant to thin film pyroelectric materials, as outlined in the following. The first point is the measurement of hysteresis. Hysteresis curves are essential for understanding the ferroelectric and pyroelectric behavior of materials. By applying an electric field and measuring the polarization response, researchers can characterize the hysteresis loop, providing insights into the switching dynamics and polarization properties of thin-film pyroelectric materials. Second, dielectric permittivity measurements involve determining the ability of a material to store electrical energy in an electric field. This is crucial for understanding the energy storage capabilities of thin-film pyroelectric materials, influencing their performance in various applications. Third, the measurement of capacitance-voltage characteristics provides information about the capacitive properties and response to varying voltages since capacitance-voltage characteristics are pivotal for understanding the charge storage and transport mechanisms in thin-film pyroelectric materials. The fourth point is that the measurement of leakage current characteristics in lead titanate (PT)-based thin films helps identify potential issues such as defects or breakdowns that could affect device performance, where it is critical to understand leakage currents to assess the integrity and reliability of pyroelectric devices. Fifth, fatigue measurements involve studying the degradation of polarization over repeated cycles. Assessing fatigue in PT-based materials is essential for predicting the lifespan and durability of pyroelectric devices. Techniques such as pulse testing or continuous cycling are employed for fatigue evaluation. Sixth, poling is a process where an electric field is applied to align the polar domains in a ferroelectric or pyroelectric material. For thin-film pyroelectric materials, optimizing the poling process is crucial for enhancing their pyroelectric performance and ensuring a stable polarization state. Then, pyroelectric measurements involve assessing the ability of a material to generate polarization in response to temperature changes. These measurements, often involving the pyroelectric coefficient (p), are fundamental for characterizing the pyroelectric properties of thin-film materials. Lastly, understanding the interplay between the processing methods and resulting electrical properties is crucial for optimizing thin-film pyroelectric materials. Factors such as deposition techniques, annealing processes, and film thickness can significantly influence the electrical characteristics of the material.

### 2.1. Measurement of the Hysteresis Curves

A P(E) loop is a plot of the charge per unit area or polarization (P) developed because of a field applied to that device (E) at a given frequency. Most testing methods of FE capacitors utilize either charge or current integration techniques for measuring hysteresis loops (see Appendix A.1). Hysteresis loops in the context of lead titanate films play a crucial role in understanding and characterizing the electrical behavior of these materials. Hysteresis, in general, refers to the phenomenon where the response of a system lags behind and depends on its history. In the case of lead titanate films, hysteresis loops are often observed in the polarization-electric field curve. These loops are indicative of the ferroelectric nature of lead titanate films. Ferroelectric materials, such as lead titanate, exhibit spontaneous polarization that can be switched by an external electric field. The hysteresis loop represents the relationship between the polarization and the applied electric field during both the polarization and depolarization processes. The significance of hysteresis loops lies in their ability to provide insights into the ferroelectric properties of lead titanate films. The loop’s shape and size can reveal information about the coercive field (the electric field required to switch the polarization) and the remanent polarization (the polarization retained when the external field is removed). These parameters are crucial in understanding the switching behavior and stability of lead titanate films. In practical applications, lead titanate films are used in various devices, including ferroelectric random access memory (FeRAM), sensors, actuators, and transducers. The ability to control and manipulate polarization in lead titanate films, as indicated by hysteresis loops, is exploited in these applications. For example, in FeRAM, the hysteresis loop characteristics help store and retrieve information based on the polarization states, providing a non-volatile memory solution. Hysteresis loops in pyroelectric materials are typically measured using a ferroelectric tester or a precision analyzer. The material is subjected to an oscillating electric field, and the resulting polarization is recorded as a function of the applied field. The hysteresis loop provides information about the ferroelectric and pyroelectric properties of the material. Understanding the coercive field, remanent polarization, and switching behavior is crucial for optimizing the material’s performance in pyroelectric applications. The hysteresis loop provides insight into the switching behavior, essential for the design of reliable infrared sensors.

Pintilie et al. [2] measured the hysteresis loop at 1 kHz using a TF 2000 analyzer in dynamic mode (aixACCT Systems GmbH, Aachen, Germany) for the epitaxial Pb(${Zr}_{0.2}{Ti}_{0.8}$)$O_{3}$(PZT) layers with both top and bottom electrodes made of SrRu$O_{3}$(SRO). 

**Figure 1:**
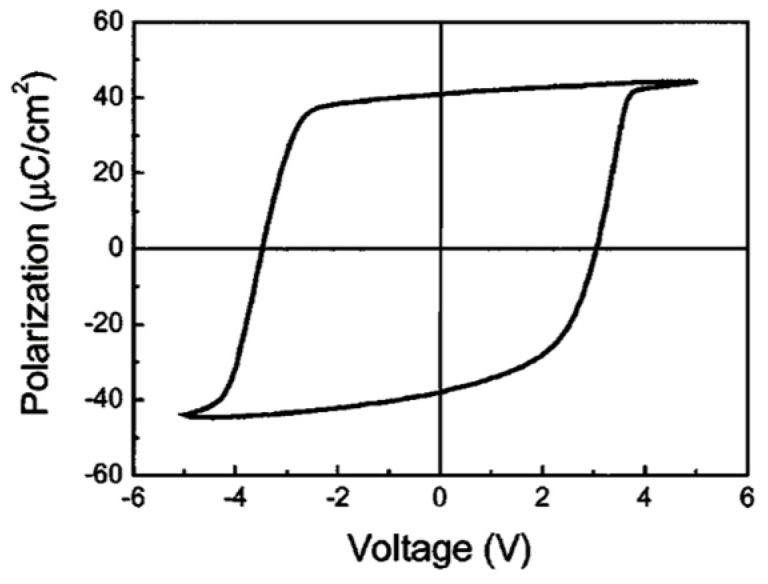
A typical hysteresis loop acquired on an epitaxial SRO/PZT (20/80)/SRO layer structure at 1 kHz [2]. Reproduced from [2], Figure 1, Figure 2, Figure 3 and Figure 4, with the permission of AIP Publishing. https://doi.org/10.1063/1.1926403.

As shown in Figure 1, typically, a remnant polarization of about 40 μC/${cm}^{2}$ and a coercive field of about 150 kV/cm was achieved [2]. The shape of the hysteresis loop is nearly rectangular and very close to an ideal loop. Yet, the reversal is not symmetric with voltage polarity, which suggests that the two FE interfaces with the electrodes are not equivalent, as discussed later.

**Figure 2:**
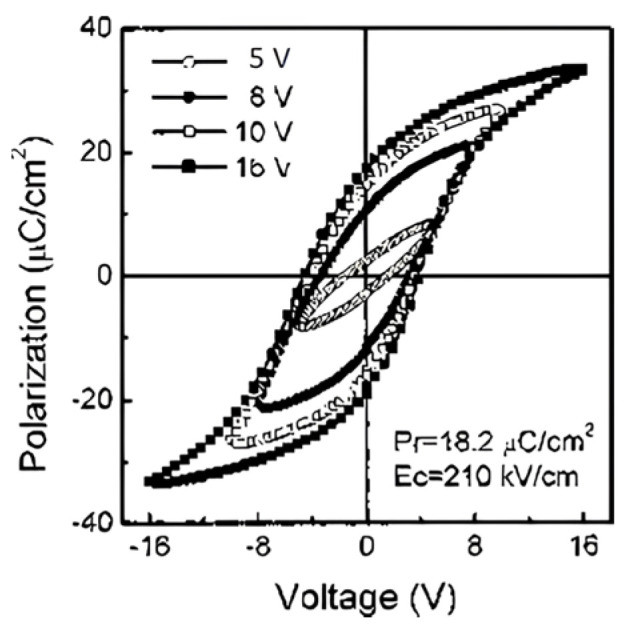
Typical P(E) hysteresis loops for the PCT film on a Pt/Ti/Si$O_{2}$/Si (100) substrate [3]. Reprinted from [3], Figure 2, Figure 3, Figure 4, Figure 5, Figure 6 and Figure 7, with the permission of AIP Publishing.

Tang, et al. [3] prepared 200 nm thick, highly (111)-oriented (${Pb}_{0.76}{Ca}_{0.24}$) Ti$O_{3}$ (PCT) thin films on Pt/Ti/Si$O_{2}$/Si substrates using a sol–gel process. Their capacitor structure consisted of top (0.2-mm diameter, Ohmic) Au/PCT/bottom (Schottky) Pt on Ti/Si$O_{2}$/Si (100) substrate. Figure 2 shows the typical P(E) hysteresis loops at an applied field of 800 kV/cm with remnant polarization ($P_{r}$) and coercive electric field ($E_{cr}$) values of 18.2 m C/${cm}^{2}$ and 210 kV/cm, respectively [3].

Figure 2 shows that the P(E) hysteresis loop is asymmetric, with an imprint voltage $\left[\left(-V_{c}\right)+\left(+V_{c}\right)\right]/2=-0.22V$. The voltage shift indicates the presence of a trap distribution near film-substrate interface [4], and its magnitude is affected by parameters such as the top and bottom electrodes’ work function difference, the magnitude of polarization, and/or the contribution of defect-dipole complexes. The polarization establishes a potential well that attracts the charge carriers to the interfacial defect sites where they are trapped. Remnant polarization and coercive electric field for several processes are compared in Table 1.

Asymmetrical contacts to FE films lead to the built-in electric fields and imprint the FE hysteresis loop toward one bias [7]. Simulation has shown that unequal Schottky barriers at the film/electrode interface are the main source of imprint [8]. Symmetrical electrodes can also give rise to the imprint due to the presence of a fabrication defect-induced passive layer within the metal/FE/metal capacitor [9]. Imprint can be beneficial since it allows the observation of high field properties even at low or zero applied fields. In pyroelectric applications, beneficial pyroelectric coefficients increase, and detrimental dielectric loss decreases at higher fields [10]. It is valuable to observe these properties at lower fields.

### 2.2. Measurement of the Dielectric Permittivity

**Figure 3:**
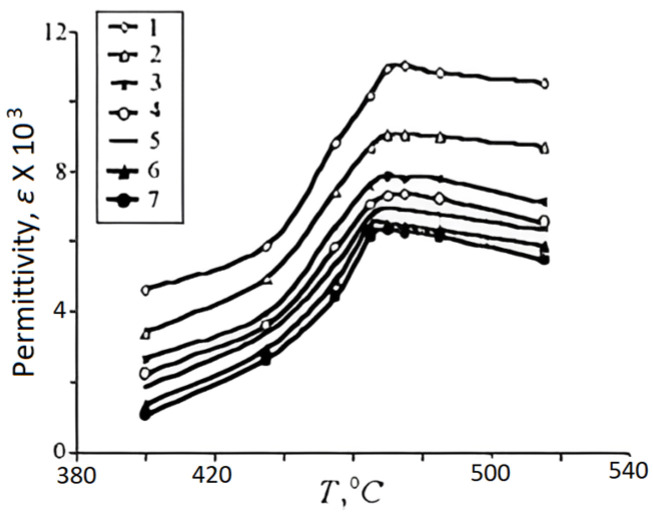
Dependencies of dielectric constant for lead titanate films at 1—1 kHz, 2—3.16 kHz, 3—7.5 kHz, 4—12 kHz, 5—18.18 kHz, 6—33.33 kHz, and 7—50 kHz [11]. Reproduced from [11], Figure 5 and Figure 6, with permission from Trans Tech Publications.

**Figure 4:**
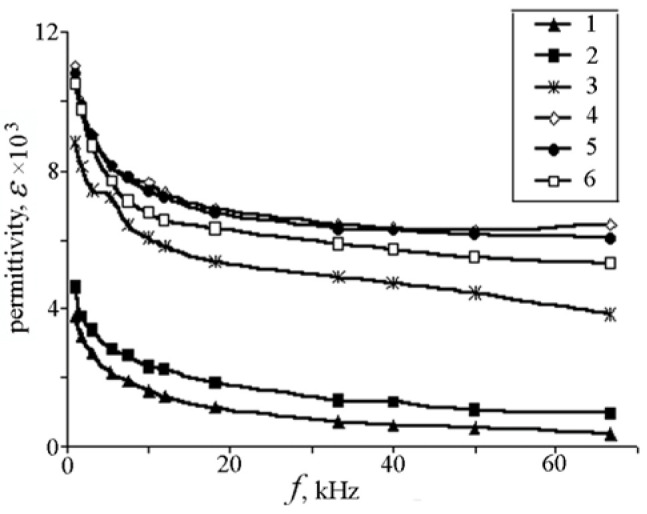
Frequency dependencies of dielectric constant for lead titanate films at 1—310 °C, 2—400 °C, 3—455 °C, 4—475 °C, 5—485 °C, and 6—515 °C [11]. Reproduced from [11], Figure 5 and Figure 6, with permission from Trans Tech Publications.

The relative permittivity ($\varepsilon_{r}$) of a dielectric (dielectric constant) indicates to what extent a finite volume of a dielectric is polarized when it is biased under an electric field. Dielectric permittivity is often measured using impedance analyzers or LCR meters. The sample is subjected to an alternating electric field, and the response in terms of capacitance and phase shift is analyzed. Dielectric permittivity is indicative of the material’s ability to store electrical energy. In pyroelectric materials, the permittivity may change with temperature, affecting the material’s overall pyroelectric response. Understanding dielectric permittivity aids in designing efficient pyroelectric energy harvesting devices that can convert temperature variations into electrical energy. Sidorkin et al. [11] prepared lead titanate films on ${Al}_{2}O_{3}$ using layer by layer magnetron sputter deposition of Ti and Pb in an argon environment, without interruption to atmosphere, in a chamber evacuated to a pressure of $3.3\times 10^{-3}$ Pa, followed by annealing in $O_{2}$ at 700 °C for one hour. Under these conditions, a (Pb/Ti)=1.25 in the deposited films provided optimal PT stoichiometry and crystallinity. The frequency dependence of ε(T), for 300 °C<T<550 °C (around of the phase transition temperature) and the temperature dependence of ε(f) for 100 Hz<f<100 kHz are shown in Figure 3 and Figure 4, respectively [11]. Measurements were carried out under an applied voltage of 0.5 V.

Curie temperature (Tc) is the temperature at which any ε(T) in Figure 3 reaches its maximum and marks the borderline between (orthorhombic) ferro- and (cubic) para-electric phases. In other words, the Curie temperature is defined as the temperature when the phase of a material changes because of the application of an electric field. Like the bulk samples, Figure 4 shows a relaxation character of dispersion: At any temperature, ε(f) is reduced as f is increased, and at high frequencies, it assumes a relatively constant value. This behavior stems from dipole inertia: Various dipoles lag behind the electric field. At low frequencies, all the dipoles contribute to the value of the dielectric constant. As the frequency is increased, those dipoles with larger relaxation time constants cease to respond and hence, the dielectric constant decreases. The delay in response leads to a loss and decline in the dielectric constant.

The dielectric constant of thin film ferroelectric materials is smaller than that of their bulk counterparts and generally decreases as the film thickness is reduced [12]. It is measured via the capacitance of parallel plate-type capacitors at the low-frequency end and is interpreted in terms of an interfacial layer with the low dielectric constant in series with the bulk dielectric. Such a rationale conforms with theoretical polarization models since the loss of dipole-dipole interactions at the film interface results in the reduction of the dielectric constant. A second source contributing to the reduction of the measured capacitance from the value expected for an ideal parallel plate capacitor is associated with electric field penetration into the metal electrodes [13,14]. However, the dielectric properties of the thin films depend on their microstructure, and it is challenging to separate the influence of thickness-dependent variations in the microstructure on the dielectric constant from intrinsic thickness variations of the dielectric constant. Studies on the dielectric properties of thin polycrystalline $Pb{Zr}_{x}{Ti}_{1-x}O_{3}$ (PZT) films conform with the general thickness dependence trend [15,16]. For epitaxial films, Fujisawa et al. [17] report that the variation of the dielectric constant with thickness is smaller compared to that of polycrystalline PZT films. Pintilie et al. [18] found that the intrinsic dielectric constant is close to the measured value in the fully depleted thin films and attributed the decrease in the dielectric constant with decreasing film thickness to the leaky bulk region of the film.

### 2.3. Measurement of Capacitance-Voltage Characteristics

The small signal capacitance is measured using a composite signal obtained by superimposing a small signal (with an amplitude 50–100 mV) AC voltage at some specific frequency, typically 1 kHz, over a DC voltage (typically a slowly varying triangular or sinusoidal waveform) that traces the hysteresis loop. Then, the component of the current will be 90° out of phase with respect to the driving AC voltage that defines the capacitance. C-V measurements involve applying a voltage across the material and measuring the resulting capacitance. This is particularly useful for understanding the electronic properties of interfaces in pyroelectric devices. C-V characteristics help determine the density of charge carriers, interface states, and the effectiveness of the material in responding to varying electric fields, which is crucial for optimizing device performance. C-V characteristics help optimize the interface properties of pyroelectric materials in thermal imaging devices, enhancing their sensitivity and resolution [2,12,19,20,21].

**Figure 5:**
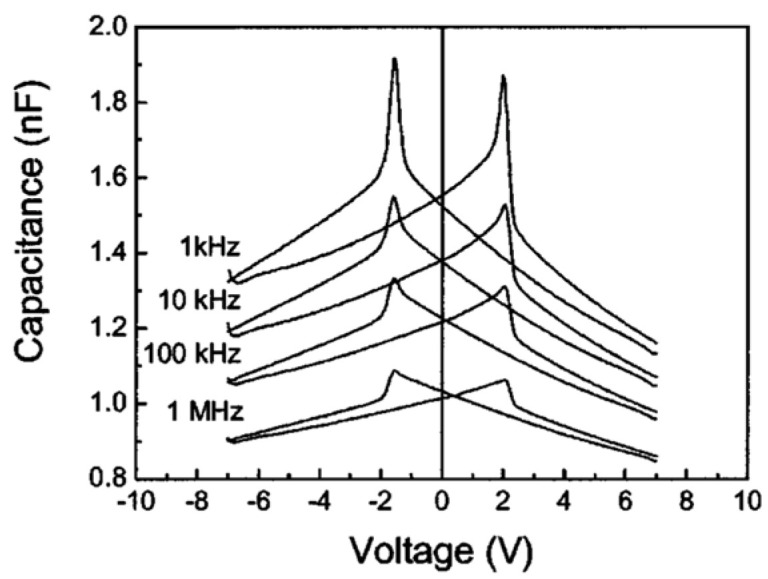
C-V characteristics at different frequencies. The AC signal was 50 mV and the step size was 35 mV. The corresponding frequency was about 0.028 Hz. The shape is the same when the voltage is swept up-down and vice versa [2]. Reproduced from [2], Figure 1, Figure 2, Figure 3 and Figure 4, with the permission of AIP Publishing.

The C-V characteristics of an FE capacitor have a butterfly-loop shape with sharp capacitance peaks at both positive and negative voltages. The peak values depend on frequency as shown in Figure 5 and transform into capacitance discontinuities at higher frequencies [2].

**Figure 6:**
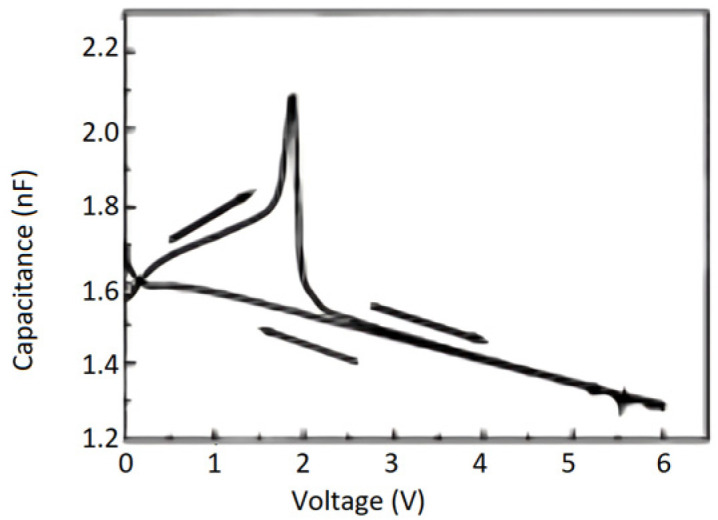
C-V characteristics at 1 kHz measured after polishing the film with 6 V for one minute [2]. Reproduced from [2], Figure 1, Figure 2, Figure 3 and Figure 4, with the permission of AIP Publishing.

Poling affects the C-V curve as shown in Figure 6. Pintilie et al. [2] established that the sharp capacitance peaks/discontinuities observed in the C-V characteristics at different frequencies are associated with the polarization reversal and took the difference between slopes toward the switching peaks for the two branches as qualitative evidence supporting their proposition. Furthermore, if the FE film acts as a large band-gap semiconductor and the SRO-PZT interface behaves as a Schottky contact, according to Pintilie et al. [2], this structure can be modeled as two Schottky diodes connected back–to–back. The PZT film was considered p-type. The built-in potential, which is a measure of the band bending near the electrode interfaces [19], is controlled by the value and sign of the polarization charge. Polarization charges have opposite effects on the built-in potentials ($V_{bi}$) at the two SRO-PZT interfaces [12]. At one interface, the polarization charge decreases $V_{bi}$ compared to the case where the polarization is absent, while at the other interface, the polarization charge increases with $V_{bi}$. Therefore, when the polarization switches, charges change suddenly, and this is accompanied by changing the sign of the polarization charges at the two interfaces. The sudden and irreversible change in charge due to polarization reversal leads to the discontinuity in C-V. Once the polarization is reversed and saturated, the built-in potential remains the same up to the coercive voltage (of opposite sign).

**Figure 7:**
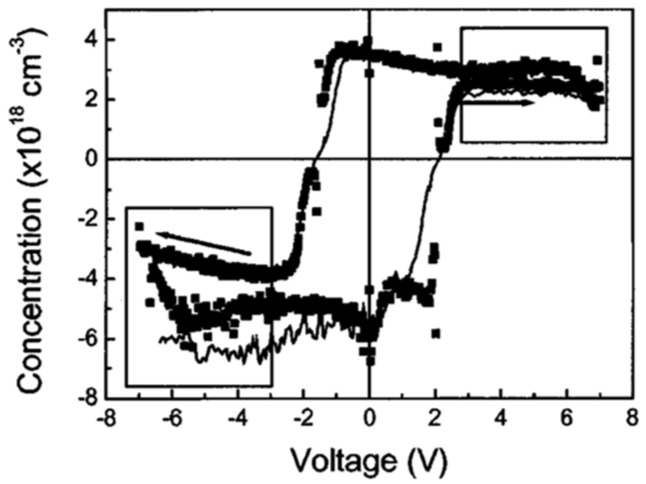
Impurity concentration calculated using (1) from the C-V measurements at 1 kHz (dots) and 1 MHz (solid line). The boxes indicate the voltage ranges in which (1) is applicable [2]. Reproduced from [2], Figure 1, Figure 2, Figure 3 and Figure 4, with the permission of AIP Publishing.

The behavior of the C-V curve for values $-V_{c}<V<V_{c}$ is governed by the voltage dependence of the dielectric constant. For $V<{-V}_{c}$ or $V>V_{c}$, the polarization becomes fully saturated, and the dielectric constant becomes relatively independent of voltage. For these regions, shown in the boxes in Figure 7, the system behaves like a normal Schottky contact with dielectric material acting as a semiconductor. Thus, the C-V can be used to extract the (donor or acceptor) impurity concentration in the dielectric material using the relation:

(1) $$C\left(T\right)=\frac{2}{qA^{2}\varepsilon_{0}\varepsilon_{r}[d(1/C^{2})/dv]}.$$

Caution must be exercised to avoid artifacts such as contributions from spurious elements in the system including electrodes, grain boundaries, leads, etc.

The DC bias sets the polarization value, while the small-signal AC voltage produces reversible movements of the domain walls. Bolten et al. [19] used this phenomenon to measure the reversible component of P and separate it from its irreversible component. Furthermore, they investigated the influence of the dielectric composition on the reversible and irreversible contribution of polarization in FE thin films. Monitoring the reversible polarization component is also helpful in determining fatigue. The electric field can be measured from the C-V curve using the Equation (2):

(2) C(E)/A=(E)/d(E).

C-V characteristics of metal-insulator-metal (MIM) systems (I=FE dielectric) bear contributions from two sources: changes in Ε and the changes in the depletion layer width, d(E), which acts as an artifact when measuring the dielectric constant of a FE material. When the field dependence of the dielectric constant explains the C-V characteristics, the dielectric acts like an ideal insulator. On the other hand, when the voltage dependence of the dielectric’s depletion layers describes C-V behavior, it acts like a semiconductor [14].

Considering the nonlinearity of the PZT’s dielectric response, Basceri et al. [20] expressed their C-V results based on a Landau-Ginzburg expansion of the polarization. Outzourhit et al. [21] calculated the change in the field due to a nonlinear dielectric response. Interestingly, both the Landau-Ginzburg expansion and the semiconductor model expansion of the polarization provided similar results. The relative size of the contributions may vary depending on the sample and experimental conditions. It can be concluded that C-V measurement may convey an ambiguous value for $\varepsilon_{r}(E)$ [2].

Domain walls contribute to the permittivity in an FE material. FE domains in a dielectric nucleate at specific sites in the vicinity of structural defects. Upon application of an external field, the domain walls propagate [17]. However, their motion is influenced by the existing local defects [18]. This phenomenon is referred to as the “pinning” of the domain wall movement by a local defect. Motion and/or pinning of domain walls can contribute to the permittivity when $E_{c}/3<E<E_{c}/2$, where the density and structure of the domain walls and phase boundaries remain unchanged with field cycling.

Domain wall pinning of PZT contributes to dielectric permittivity according to Raleigh law. It has two components: a reversible component—$\left(\varepsilon_{init}^{\prime }\right)$, the relative dielectric constant and an irreversible component—$\left(\alpha_{\varepsilon }^{\prime }E_{ac}\right)$, both of which contribute to the dielectric permittivity [19,20,21]:

(3) $$\varepsilon^{\prime }=\varepsilon_{init}^{\prime }+\alpha_{\varepsilon }^{\prime }E_{ac},$$

### 2.4. Measurement of Leakage Current Characteristics in PT-Based Materials

The leakage current reduces the overall efficiency of the pyroelectric detectors. In thin insulating films, the current depends on the applied voltage (V), time (t), and temperature (T). A compilation of possible transport mechanisms in thin films is provided in Appendix A.1. PT-based materials exhibit different conduction behavior depending on physical parameters such as film microstructure, thickness, doping level, and electrode materials. Among different PT-based materials, PZT has been the focus of much attention due to its applicability in ferroelectric random access memory (FERAM) technology and has been prepared by the sol-gel technique almost exclusively. 

#### 2.4.1. Metal/PZT Band Diagram

To identify leakage mechanisms in PT-based dielectric insulators, it is crucial to consider the band diagram of the metal-semiconductor (M-S) contacts. In this section, we discuss the electronic properties of PZT material. We begin with the theoretical analysis of the Pt/PZT interface.

PZT is a relatively wide bandgap semiconductor with $E_{g}=3.41\;eV$ and generally has p-type conductivity. Raymond and Smyth [22] studied polycrystalline bulk samples at high temperatures (500 °C<T<700 °C). At such high temperatures, both grain boundary and bulk conduction may contribute to the transport mechanism. Unintentionally doped PZT possesses a large concentration of acceptor impurities but is generally compensated by the formation of oxygen vacancies ($V_{o}^{++}$), which leads to a relatively small concentration of the active acceptors. Hence, the Fermi level ($E_{F}$) resides at energy relatively far from the valence band edge ($E_{V}$).

**Figure 8:**
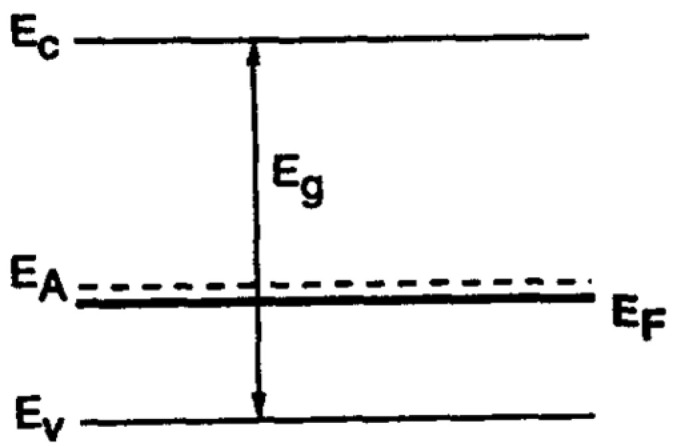
PZT energy band diagram (for 200 °C<T<350 °C) [22]. Reprinted from [22], Figure 1, Figure 2, Figure 3, Figure 4 and Figure 5, with permission from Elsevier.

Due to the large ionization energy (0.71 eV) of the acceptor impurity level ($E_{A}$), the impurities freeze out at lower temperatures (200 °C<T<350 °C). In this case, $E_{F}$ resides below $E_{A}$, as shown in Figure 8. Except for the deep hole trap formed by the acceptor impurities, ${Pb}^{+3}$ can act as a shallow hole trap and ${Ti}^{+3}$ as a deeper (~1 eV) electron trap. These traps can influence the free-carrier conduction in PZT via Poole-Frenkel or hopping conduction [12,23,24]. Two kinds of crystalline structures are important in thin-film PZTs:

1. fine-grained films, with grain boundaries (GB) parallel and perpendicular to the current flow, and
2. columnar grain structures, with GBs parallel to the current flow.

Grain boundaries can act as barriers when they stand perpendicular to the current flow. These structural differences may result in a variety of I(V) behaviors. In the following analysis, to simplify the discussion, PZT film is assumed to be a single crystal material.

Below T=600 °C, PZT accommodates only a minute concentration of holes and thus, behaves very much like an insulator. Consequently, in a PZT ferroelectric capacitor (FECAP) with blocking contacts, a supply of carriers at the metal-PZT contacts is required if the charge is to be transported across the insulator.

**Figure 9:**
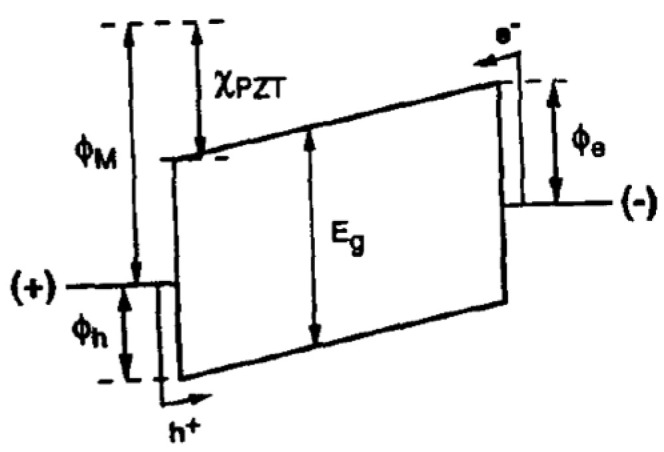
Definition of the electron ($\Phi_{e}$) and hole barrier ($\Phi_{h}$) for injection from the metal into fully depleted PZT, with $\Phi_{pt}=5.56\;eV$, $\chi_{PZT}=2.6\;eV$, $\Phi_{h}=0.36\;eV$, and $\Phi_{e}=3.05\;eV$ [22]. Reprinted from [22], Figure 1, Figure 2, Figure 3, Figure 4 and Figure 5, with permission from Elsevier.

Figure 9 depicts the energy band diagram proposed by Wouter et al. [22] for the Pt/PZT/Pt structure, which predicts a hole barrier of $\Phi_{h}=0.36\;eV$ and an electron barrier of $\Phi_{e}=3.05\;eV$ for injection from the metal into the fully depleted PZT. Assuming PZT behaves like a fully depleted “insulator”, Pt contacts are employed and Schottky theory applies ($\Phi_{m}=5.62\;eV$) [23]. Wouter et al. [22] argued that hole injection prevails. The electron affinity for PZT ($\chi_{PZT}$) is derived by assuming $E_{F}-E_{V}\sim 0.65\;eV$ and using the experimental value of $\Phi_{PZT}=5.35\;eV$ for the PZT work function [24].

At higher temperatures, oxygen vacancies ($V_{o}^{++}$), which act as compensating negatively charged acceptors, are mobilized and drifted under the influence of the electrical field, induced by contact potential, for example [25]. These charges will then accumulate near the Pt electrode and provide the required positive space charge needed to neutralize the negative electrode charges caused by contact potential.

This process is important during the annealing of the Pt-PZT interface [26].

**Figure 10:**
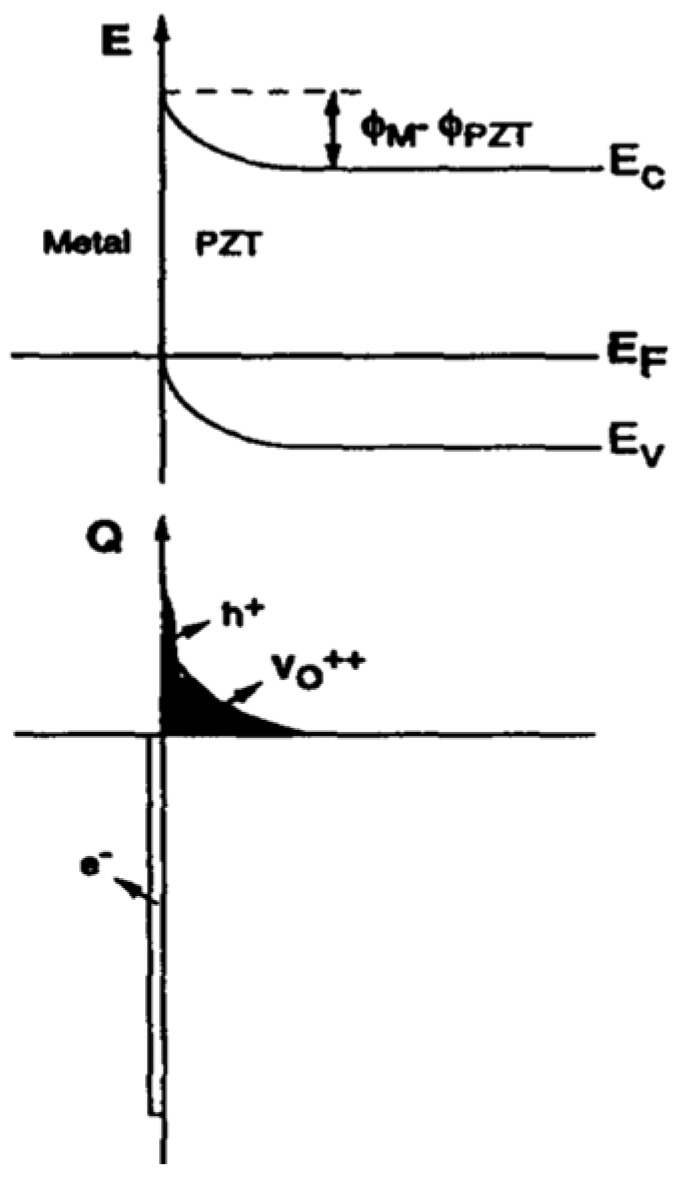
Band bending and space charge at a heat-treated Pt-PZT contact [22]. Reprinted from [22], Figure 1, Figure 2, Figure 3, Figure 4 and Figure 5, with permission from Elsevier.

If the metal is negatively (or positively) biased relative to the semiconductor (PZT), the required balancing charge in the semiconductor (PZT) interfacial region is positive (or negative), which must be supplied through hole accumulation (hole depletion, i.e., ionized acceptors or inversion) and is accompanied by an upward (or downward) bending of the energy bands. For the lightly p-doped compensated PZT films, the concentration of the holes in the thin film is inadequate to support balancing charges on the electrodes. Hence, negligible band bending may occur even when a large negative (or positive) voltage is applied. As a result, in both cases, PZT will become fully depleted and behave as an ideal insulator. Since $\Phi_{Pt}$ > $\Phi_{PZT}$, $V_{o}^{++}$ accumulates near the Pt contact during the annealing. Throughout the cooling down period, however, the space charge created remains frozen-in near the Pt/PZT interface. This causes the bands to bend up, as shown in Figure 10.

While the oxygen vacancies freeze-in at lower temperatures, the accumulated holes remain mobile. If this carrier reservoir can be easily refilled, either by carriers supplied by the metal (when $E_{F}<E_{V}$), or by fast carrier generation, an Ohmic contact is achieved, which enables a hole current to flow from the positively biased contact through the PZT film. At least in a low current regime, this hole current is not controlled by the interface but by the conduction process in the bulk of the PZT film.

The hole accumulation region eventually disappears if the bias is increased, and the contact returns to a blocking state, where the current will become interface-limited, and holes can only be injected into the PZT film by crossing over the barrier.

The non-annealed Pt-PZT interfaces present a poor interface, which further impedes the injection of carriers. Then, ionic conduction becomes dominant.

In contrast to bulk models, the current increase in the current-degradation regime is not caused by bulk conductivity changes but by (increased) carrier injection at the Pt-PZT interface, while either the carrier injection mechanism or bulk conduction processes may limit the current in the saturation current region.

#### 2.4.2. Leakage Mechanisms in PT-Based Materials

Various processing steps are utilized for the preparation of PT-based thin films, which lead to different electrical properties. Consequently, numerous physical models have been proposed to describe the leakage current behavior in PZT films. A summary of the proposed conduction mechanisms in PT-based films is provided in Table 2 and has been analyzed by Wouters [22] (see Appendix A.1).

**Figure 11:**
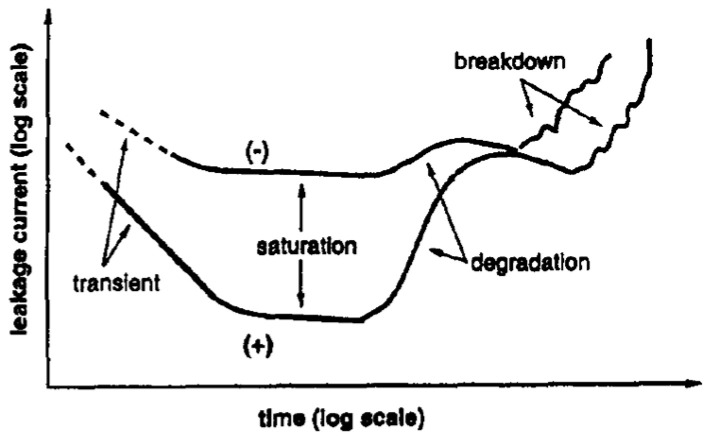
Schematic I(t) characteristics of a Pt-PZT-Pt PECAP after the application of a (+) and (−) DC voltage to the top electrode of an unannealed sample [22]. Reprinted from [22], Figure 1, Figure 2, Figure 3, Figure 4 and Figure 5, with permission from Elsevier.

Figure 11 shows the typical transient, saturation (true leakage), and degradation regime, as well as the breakdown region commonly observed in I(t) characteristics of Pt-PZT-Pt capacitors. Four distinct regimes in I(t) characteristics of the Pt-PZT-Pt FECAP can be identified [27]:

1. The transient current: This is the initial decrease in current with time.
2. The saturation (“true” leakage), which becomes pronounced after current transients are died out and predominates leakage for a certain period.
3. The resistance degradation regime, where I(t) increases until a maximum value is reached.
4. The dielectric breakdown region.

Thin polycrystalline alkaline-earth titanates normally contain substantial concentrations of positively charged oxygen vacancies. Thus, in a DC electrical field, they migrate toward the cathode. This makes it easier for carriers (electrons) to overcome the barrier because it modifies the shape of the barrier. These vacancies are positively charged with respect to the regular lattice and are commonly held responsible for resistance degradation [29,39] that provides a quantitative analytical model for the resistance degradation regime.

**Figure 12:**
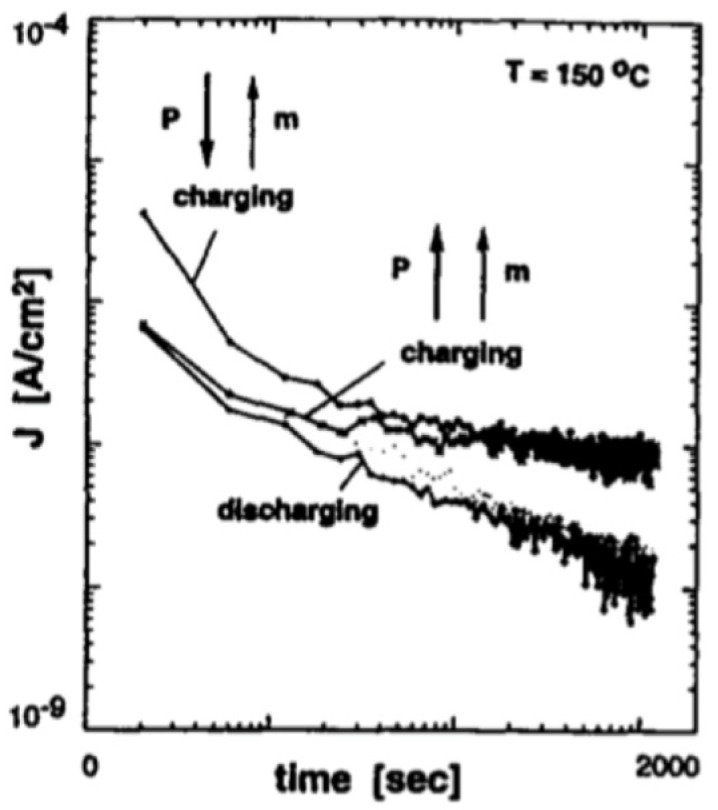
Charging and discharging currents after pre-poling the sample with a poling field parallel or antiparallel to the measuring field [22]. Reprinted from [22], Figure 1, Figure 2, Figure 3, Figure 4 and Figure 5, with permission from Elsevier.

Figure 12 shows the charging and discharging currents measured at T=150 °C. This sample was pre-poled with a poling field parallel or antiparallel to the applied electric field. The magnitude of I(t) depends on the sample history [22]. These observations show that the identification of the conduction mechanism (s) in PT-based films is complex. This is because:

1. The data on I(V) and I(t) measurements depend on the separation technique used for the “true” leakage current from the dielectric relaxation current. In perovskites, the current comprises a time-dependent component dielectric relaxation, which is strongly dependent on the measurement technique, prior history of the sample, and other factors, as seen in Figure 12.
2. Often, different conduction mechanisms result in similar I(V) curves, especially when the analysis is carried out on a limited voltage interval.
3. Different processing factors and physical parameters including microstructure, thickness, doping level, and electrode materials affect conduction behavior in polycrystalline films.

Thus, a comprehensive experimental study on transport mechanisms in PT-based perovskite dielectrics requires the utilization of diverse measuring techniques for samples prepared with various processing parameters such as thickness, grain structure, interface structure, and doping levels.

In the case of perovskite materials, the leakage current can be described as a superposition of two components: the relaxation current, [$J_{0}\left(V,\;T\right)t^{-n}$] which tends to vanish as the stress time is increased, and the “true” leakage current, $J_{true}$ which reflects the real evolution of leakage current inside the dielectric [22,40]. Although the true leakage current is usually considered static, it does evolve with time. To consider this time evolution, two complementary factors is introduced: the resistance degradation factor $\varphi_{RD}(V,\;T)$ [41] and the resistance restoration factor $\varphi_{RR}(V,\;T)$ [42].

(4) $$J\left(V,\;T,\;t\right)=J_{0}\left(V,\;T\right)t^{-n}+J_{true}\varphi_{RD}(V,\;T)\varphi_{RR}(V,\;T),$$

where n is the slope of J(t) in the log-log plot. $J_{true}$ becomes predominant after a certain time has elapsed [43]. There are three well-known mechanisms that can give rise to the exponential dependence of current density on the elapsed time; these are space-charge trapping, relaxation time distribution, and electrical charge hopping [44].

The precise determination of conduction mechanisms requires accurate measurements of the “true” leakage current. Measurements of J(V) and J(T) characteristics must be carried out in the saturated current regime at 100 °C<T<200 °C. The time dependency of the current density makes it challenging to obtain J(V) curves representative of the true leakage current.

1. Dielectric relaxation regime.

The first term on the right-hand side of Equation (4) follows a Curie Von-Schweidler law $\left(J\propto t^{-n}\right)$ and is due to dielectric relaxation [42]. As shown in Figure 11, it is attributed to the transient regime and characterized by the initial decrease in current due to the displacement of charges [45]. For PT-based insulators, the relaxation mechanism is pronounced at T<125 °C, while the term $J_{0}(V,\;T)$ is thermally activated with an activation energy ~0.3 eV, and the slope in relaxation regime (n) appears to be temperature-independent, with n=0.55. Several authors proposed possible mechanisms responsible for the dielectric relaxation process for PT-based materials. Chen et al. [45] attributed dielectric relaxation to either the electrical charge hopping or to a Maxwell-Wagner polarization with a wide distribution of relaxation times. Nagaraj et al. [46] assumed oxygen vacancies as well as ${Ti}^{4+}$ ions as possible trapping sites and considering the energy levels of these defects in the band gap of PZT, they attributed dielectric relaxation to charge entrapment in respective trapping sites. On the other hand, Simons [47] suggested that electrons associated with oxygen vacancies account for the relaxation current.

2. The saturation region.

This is the relatively constant low-level current density component in Figure 11 and represents the true leakage current. It can be described as a thermionic Schottky injection of holes through the anode/PZT interface, which follows the relationship:

(5) $$J_{true}=A^{*}T^{2}\exp\left(-\frac{\Phi_{ini}}{kT}\right),$$

where $A^{*}$ is the Richardson constant, $\Phi_{ini}$ is the initial potential barrier height at the anode interface, and k is the Boltzmann constant. This region is characterized by the fact that the charging and discharging currents are not identical [45]. Saturation is not usually a prominent component of the leakage current at room temperature even after a few 1000 s. Except for small biases, it is best revealed in the temperature range of 100 °C<T<125 °C.

3. Resistance degradation and restoration region.

This is the term attributed to the increasing current region in Figure 11, next to the saturation region. The amount of increase in the current depends on the sample, which is noticeable for positively biased samples with unannealed top electrode [41] (not to be confused with dielectric breakdown). Oxygen vacancies are present in significant concentrations in alkaline-earth titanates. They are positively charged with respect to the regular lattice. In a DC electric field, they migrate toward the cathode. The redistribution of the oxygen vacancies near the interfaces alters the shape of the barrier for carrier injection, making it easier for electrons to overcome [41]. This increase in current density may be modeled by a decrease in the effective barrier height at the cathode. Zafar et al. [39] modeled this resistance degradation region by a factor $\varphi_{RD}\left(V,\;T\right)$ as:

(6) $$\varphi_{RD}\left(V,\;T\right)=\exp\left(-\frac{\Delta \Phi_{1}\left(V,\;T,t\right)}{kT}\right),$$

where $\Delta \Phi_{1}\left(V,\;T,t\right)$ represents the metal/PZT barrier height lowering [41]. The onset of this current degradation regime is shifted to shorter times with increasing temperature and voltage [41], making it difficult to distinguish the transition from the saturated current regime to the resistance degradation regime. 

As the time elapsed, oxygen vacancies accumulated near the neutralized cathode. Consequently, space charge density is decreased at the cathode interface, for which electron injection may be modeled by an effective increase in the barrier height leakage, which reaches a maximum and then starts to decrease. This behavior is modeled by the resistance restoration $\varphi_{RR}\left(V,\;T\right)$ factor [42]:

(7) $$\varphi_{RR}\left(V,\;T\right)=\exp\left(-\frac{\Delta \Phi_{2}\left(V,\;T,t\right)}{kT}\right),$$

where $\Delta \Phi_{2}\left(V,\;T,t\right)$ describes the barrier height restoration potential. Then, after a certain period of stress time, the metal/PZT barrier height tends to recover its initial value due to space charge reorganization induced by lead vacancies. 

To investigate the conduction processes, measurements of the I(V) and I(t) characteristics should be carried out in the saturated current regime (100 °C<T<200 °C) [41].

#### 2.4.3. The Effect of Dielectric Thickness on Leakage Current Mechanisms

**Figure 13:**
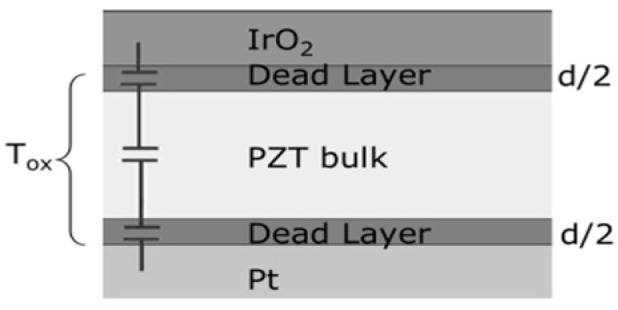
Schematic representation of three series capacitors modeling for dead layer thickness extraction [27]. Reprinted from [27], Figure 5, Figure 7, Figure 8 and Figure 14, with the permission of AIP Publishing. https://doi.org/10.1063/1.3055416.

Chentir et al. [27] investigated the evolution of leakage current in PZT capacitors as a function of dielectric thickness. A protocol was introduced, which avoids artifacts commonly encountered in the measurement of current in perovskite dielectrics (Appendix A.4). A change in leakage current mechanism from Schottky to Poole–Frenkel (PF) mechanisms (Appendix A.3) was observed as PZT thickness decreased and was accompanied by a drift in the value of the device capacitance. Modeling the capacitor as in Figure 13 provides an estimate of ~40 nm thickness for the “dead layer”, although the nature of such layer has long been a matter of much controversy [29,48]. The switch from an interface-limited conduction mode to a bulk-limited mode is attributed to the presence of a higher defect concentration due to oxygen vacancies (in comparison with PZT bulk) in the dead layer present at PZT-electrode interfaces.

#### 2.4.4. Case Study: Leakage Current Mechanisms in Au/PCT/Pt Structure

Tang et al. [3] measured the room temperature I(V) characteristics of the Au/PCT/Pt structure under different applied DC voltages (with the positive or negative potential connected to the Pt and maintained for 3 s). The dielectric relaxation current versus time characteristics of the Au/PCT/Pt capacitor was measured after removal of the DC field.

**Figure 14:**
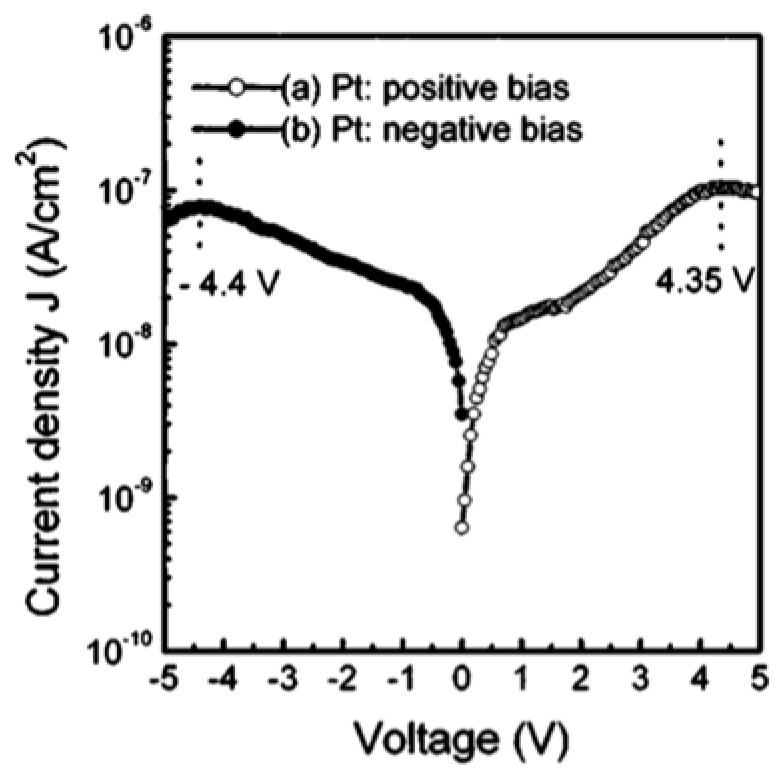
Leakage current density vs. voltage characteristic for the Au/PCT/Pt structure [3]. Reprinted from [3], Figure 2, Figure 3, Figure 4, Figure 5, Figure 6 and Figure 7, with the permission of AIP Publishing.

Figure 14 shows the current density as a function of voltage with Pt electrode biased at −5 V<V<5 V. At low electric fields with negatively biased Pt electrodes, the Pt/PCT interface exhibits a Schottky barrier characteristics, while the Au/PCT interface forms an Ohmic contact. This explains the dependence of leakage current density on the bias polarity.

**Figure 15:**
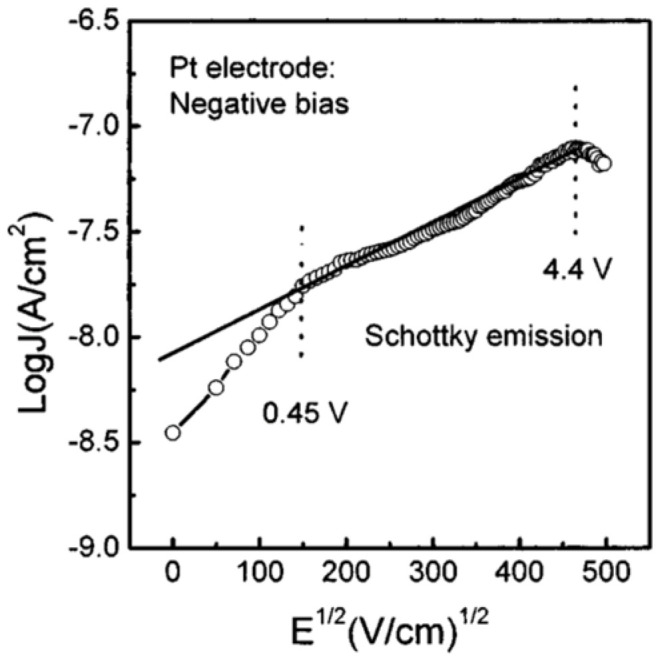
LogJ vs. $E^{\left(1/2\right)}$ plot when the Pt bottom electrode of the Au/PCT/Pt thin-film capacitor is under a negative bias [3]. Reprinted from [3], Figure 2, Figure 3, Figure 4, Figure 5, Figure 6 and Figure 7, with the permission of AIP Publishing.

As shown in Figure 15, when the Pt electrode is biased negatively, the Au/PCT interface forms an Ohmic contact for V<0.45 V. Schottky emissions over the barrier dominate when 0.45 V<V<4.4 V.

**Figure 16:**
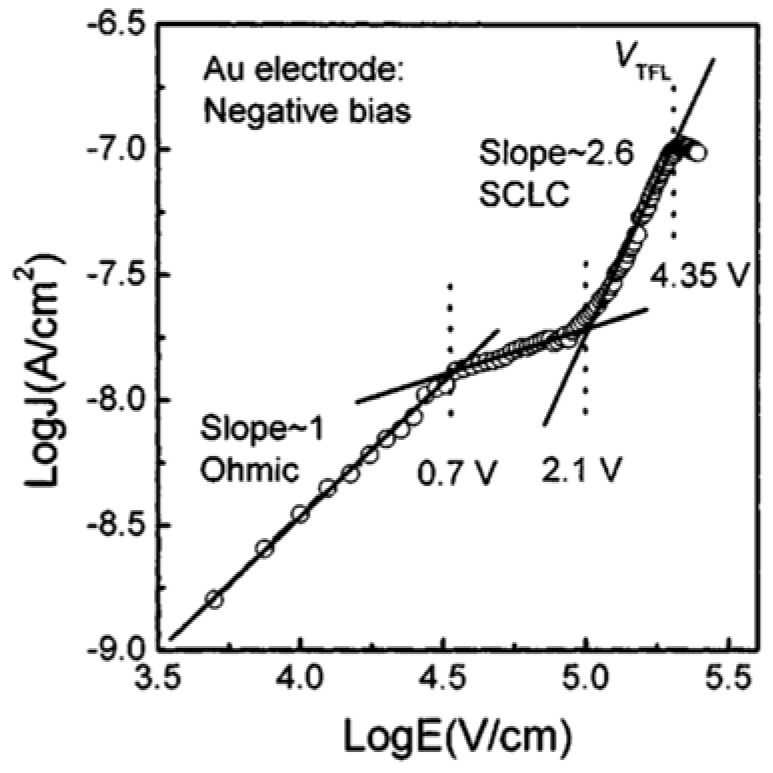
LogJ vs. LogE plot when the Au electrode of the Au/PCT/Pt thin-film capacitor is negatively biased. The conduction current shows space-charge-limited behavior for 2.1 V<V< 4.35 V when the Au electrode is negatively biased [3]. Reprinted from [3], Figure 2, Figure 3, Figure 4, Figure 5, Figure 6 and Figure 7, with the permission of AIP Publishing.

Figure 16 indicates that an Ohmic contact is formed when V<0.7 V, while the current obeys SCLC behavior (when 2.1 V<V<4.35 V) and the voltage range of 0.7 V<V<2.1 V marks the region where conduction mechanism is transformed from Ohmic to SCLC. The trap-filled limit voltage $\left(V_{TFL}\right)$ is 4.35 V. The dielectric relaxation current behavior of Au/PCT/Pt capacitor conforms to the universal (Curie–von Schweidler) law at a low electric field. At higher fields, the current has contributions from both a dielectric relaxation current and a leakage current.

**Figure 17:**
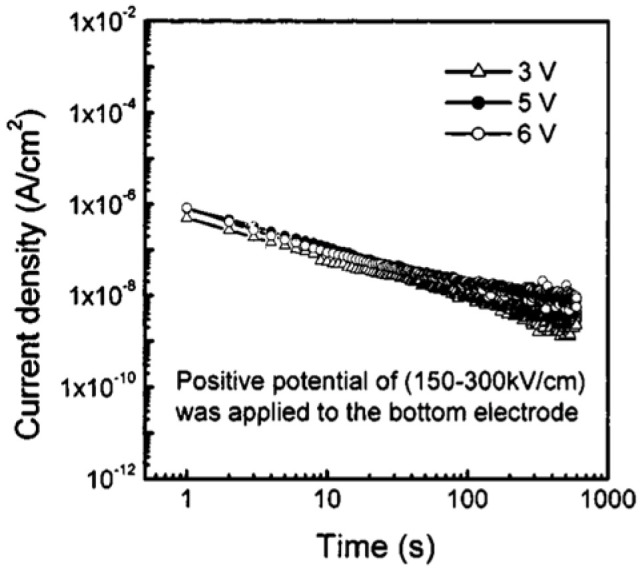
Dielectric relaxation current of the Au/PCT (24)/Pt capacitor with different positive bias voltages applied to the Pt bottom electrode [3]. Reprinted from [3], Figure 2, Figure 3, Figure 4, Figure 5, Figure 6 and Figure 7, with the permission of AIP Publishing.

Figure 17 depicts the J(t) for several bias voltages applied to the bottom Pt electrodes. The currents measured at low fields (150 and 250 kV/cm) indicate the contribution of pure dielectric relaxation currents.

**Figure 18:**
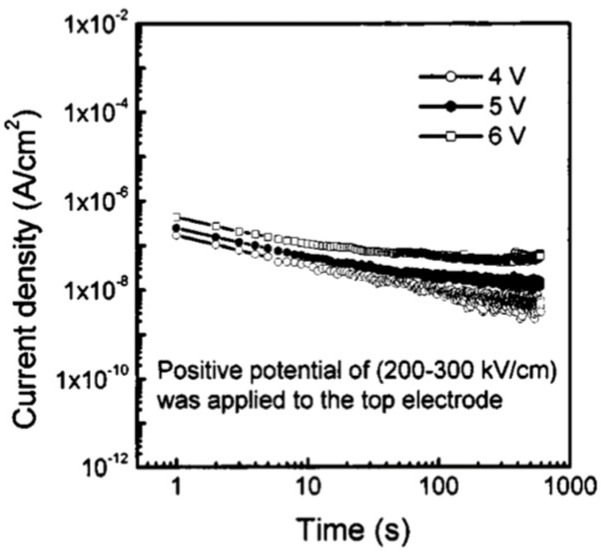
Dielectric relaxation current of the Au/PCT (24)/Pt capacitor with different positive bias voltages applied to the Au top electrode [3]. Reprinted from [3], Figure 2, Figure 3, Figure 4, Figure 5, Figure 6 and Figure 7, with the permission of AIP Publishing.

The dielectric relaxation current behavior of Au/PCT (24)/Pt capacitor obeys the Curie–von Schweidler law at low electric fields, with n=0.52 and 0.65 at E=150 and 250 kV/cm, respectively. Similarly, when positive potential is applied to the Pt bottom electrode at low fields, J(t) indicates the contribution of pure relaxation current (Figure 18). While at higher fields (250 and 300 kV/cm), the current has contributions from both a dielectric relaxation current and a leakage current, which itself comprises effects of oxygen vacancy concentration, the magnitude of polarization, space charge trapping, electrical charge hopping, and/or the contribution of defect-dipole complexes.

### 2.5. Measurement of Fatigue in PT Based Materials

Considerable disagreement exists in the literature regarding fatigue mechanisms in Pt/Pb (Zr, Ti)$O_{3}$/Pt (Pt/PZT/Pt). Dimos [49] attributed fatigue to domain pinning, while Desu et al. [50] corelated fatigue to the space charge. Conductive oxide electrodes are known to improve fatigue properties of FE memory devices. When Pt electrodes are replaced by oxide electrodes such as Ru$O_{2}$, SrRu$O_{3}$, and (La, Sr) Co$O_{3}$(LSCO), the capacitors show excellent fatigue and imprint properties [51,52]. It has been proposed that oxide electrodes act as sinks for oxygen vacancies, thereby eliminating fatigue. To understand fatigue and degradation mechanisms in Pt/Pb (Zr, Ti)$O_{3}$/Pt (Pt/PZT/Pt) heterostructures, Nagaraj et al. [26] investigated leakage current mechanisms in lead-based thin-film FE capacitors. Distinct differences exist between the electrical behavior of Pt/PZT and LSCO/PZT capacitors, which may stem from their interface disparities. Leakage in Pb based perovskite-type titanates Pb (Zr, Ti)$O_{3}$ thin films has been previously investigated [37]. Studies on Pt/PZT/Pt structures reported Schottky emission with a barrier potential of 1.41 eV [45,53,54]. 

**Figure 19:**
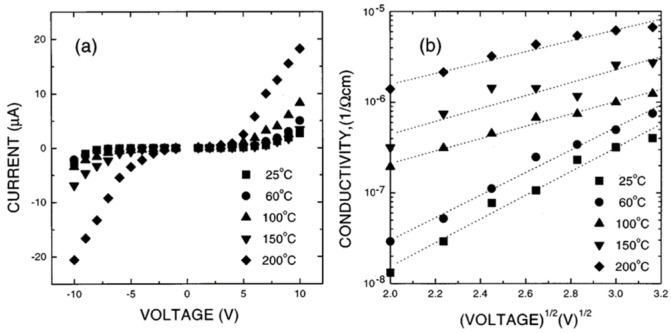
(**a**) I-V-T plots for a typical LSCO/PZT/LSCO capacitor showing nonlinear I-V characteristics and positive TC current. (**b**) $\ln\sigma -V^{\frac{1}{2}}-T$ plot for a typical LSCO/PZT/LSCO capacitor showing linear trend at fields higher than $10^{5}$ V/cm [26]. Reprinted from [26], Figure 2, Figure 3 and Figure 5, with permission from the American Physical Society.

Nagaraj et al. [26] prepared epitaxial (La, Sr) Co$O_{3}$/Pb (Zr, Sr) $O_{3}$/(La, Sr) Co$O_{3}$ capacitor structures to investigate the dominant leakage current-voltage, I(V) and the behaviors of such capacitors. Epitaxial growth of (La, Sr) Co$O_{3}$ was purposely utilized to eliminate the effects associated with grain boundaries. It has been established that PZT and PLZT capacitors show Ohmic conduction at low fields (10 kV/cm), with a slope of nearly 1 (Figure 19). The I(V) curves are (i) nearly symmetric, (ii) dependent on temperature, and (iii) possess a positive temperature coefficient (TC).

**Figure 20:**
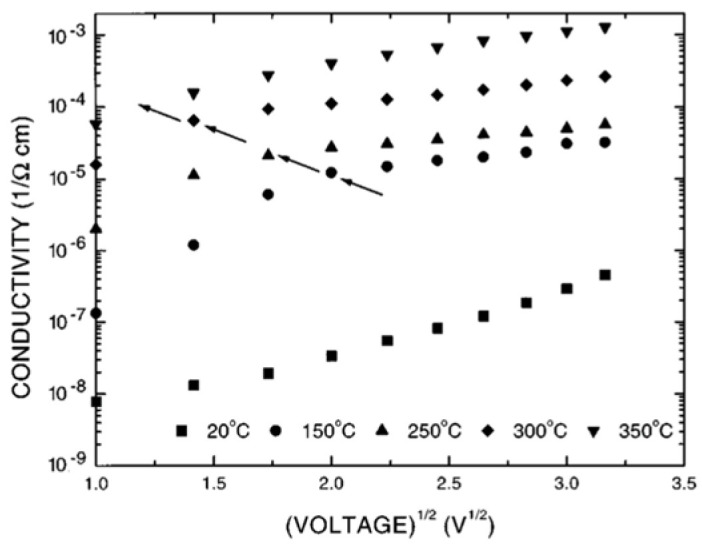
This $\ln\sigma -V^{\frac{1}{2}}-T$ plot for a typical LSCO/PZT/LSCO capacitor at fields higher than $10^{4}$ V/cm [26]. Reprinted from [26], Figure 2, Figure 3 and Figure 5, with permission from the American Physical Society.

**Figure 21:**
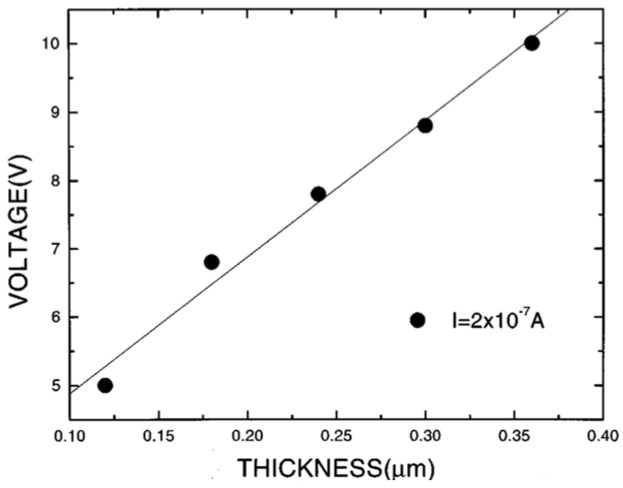
V(d) plot at a constant current $\left(I=2\times 10^{-7}A\right)$ in LSCO/PZT/ LSCO capacitor showing a linear trend indicating a uniform field in the bulk of PZT [26]. Reprinted from [26], Figure 2, Figure 3 and Figure 5, with permission from the American Physical Society.

I(V) is nearly symmetrical but nonlinear at higher fields and temperatures. Figure 20 shows the plots $\ln\sigma -V^{\frac{1}{2}}$ at different temperatures. A linear relationship is detected at high temperatures and fields. From the slope of these curve, ε≈7 is obtained for the PZT and PLZT films. This behavior also suggests that at high fields and temperatures, conduction is dominated by field-enhanced thermal ionization of trapped carriers (i.e., Poole-Frenkel emission). Figure 21 shows the plot of voltage versus thickness (V-d) at a constant current $\left(I=2\times 10^{-7}A\right)$ in the LSCO/PZT/ LSCO capacitor. The linear trend indicates a uniform field in the bulk of PZT.

The fact that the electric field is uniform in the FE film and that both temperature and field activate the current further confirms that PF emission is responsible for the transport mechanism. The trap ionization energies calculated from the slope of lnσ_0 versus 1/T plots are in the range 0.5–0.6 eV for PZT and PLZT capacitors. This suggests that the same trap center acts as the P-F center in both PZT and PLZT films. The fact that the ionization energy of ${Ti}^{4+}$ to ${Ti}^{3+}$ is 0.5 eV suggests that ${Ti}^{4+}$ could be the possible PF center. No space-charge region is created at the (PZT or PLZT)/LSCO interface. This could be the reason for good fatigue and imprint characteristics in these capacitors.

Bouyssou et al. [55] investigated the reliability properties and the degradation mechanisms in the Ir$O_{2}$/Pb${Zr}_{x}{Ti}_{1-x}O_{3}$ (PZT)/Pt capacitors by characterizing leakage current conduction and provided a model for the evolution of current in the Ir$O_{2}$/PZT/Pt capacitor as a function of time, voltage, and temperature.

The leakage current conduction in Ir$O_{2}$/PZT/Pt structures was found to be controlled by the energetics of the barrier height at the cathode interface. Hence, the voltage and temperature evolution of leakage current were interpreted as an interface-controlled thermionic injection of carriers over a potential barrier at the cathode/PZT contact. The time evolution of the leakage current is mainly characterized by the resistance degradation phenomenon. The model is based on the redistribution of oxygen vacancies near the cathode interface, but also includes the role of oxygen vacancies on dielectric relaxation and trapping phenomena.

### 2.6. Poling of Pyroelectric Thin Films

Poling is the process carried out to achieve maximum possible FE polarization parallel to an external electric field. During this process, domains with a favorable polarization direction are encouraged to grow at the expense of domains with unfavorable directions. In an unpoled polycrystalline thin film, most grains have an unfavorable crystallographic orientation which prevents the polarization direction to attain a direction perpendicular to the substrate plane and hence leads to a reduction of the maximum.

Once poled, an FE sample will remain poled unless it is de-poled, via repetitive heating and cooling through its transition temperature.

Kohli et al. [56] studied poling conditions for <111> oriented Pb_1−x_Ca_x_Ti$O_{3}$(PCT), with 30%<x<50% as well as <111> and <l00> oriented PbZrTi_1−x_Ti$O_{3}$, with x=15% (PZT 15/85) thin films. Ca addition to PbTi$O_{3}$ decreased the tetragonality of the unit cell and thus reduced the Tc, which in turn resulted in higher pyroelectric coefficients. Kohli et al. [56] also used the controlled heating-rate technique [57] to achieve reduced values for dielectric permittivity for the PCT15 $\left(3<\varepsilon_{r}<310\right)$ by controlling porosity by adjusting nucleation and growth of their sol-gel grown films.

**Figure 22:**
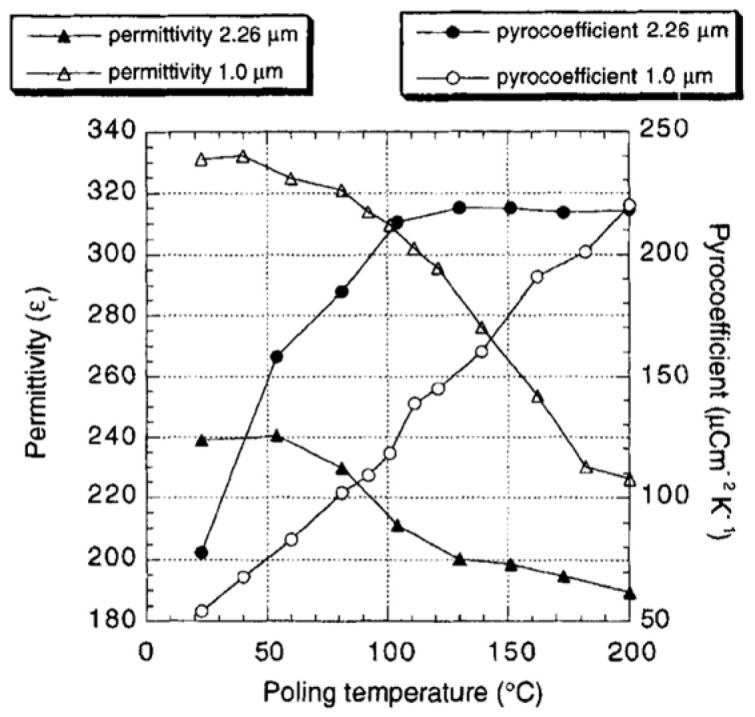
Permittivity and pyroelectric coefficient as a function of the poling temperature for (111) oriented PZT 15/85 thin films. The films were poled at 250 kV/cm for 10 min [56]. Reprinted from [56], Figure 1, Figure 3, Figure 4 and Figure 5, with permission from the Copyright Clearance Center Inc. (CCC) on Taylor and Francis’s behalf.

The effect of poling temperature on p and $\varepsilon_{r}$ at a constant field of 250 kV/cm is shown in Figure 22. In both films, the pyroelectric coefficient increases with the poling temperature, whereas the permittivity as well as the dielectric loss tangent decreases.

**Figure 23:**
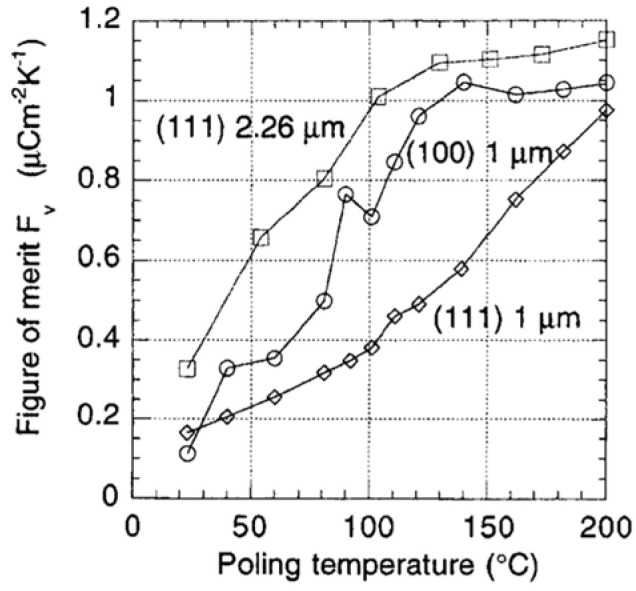
Voltage figure of merit $F_{v}=p/\varepsilon_{r}$ versus poling temperature (poled at 250 kV/cm) of (111) and (100) oriented PZT 15/85 thin films [56]. Reprinted from [56], Figure 1, Figure 3, Figure 4 and Figure 5, with permission from the Copyright Clearance Center Inc (CCC) on Taylor and Francis’s behalf.

Two figures of merits are of interest: one for voltage response $F_{v}=p/\varepsilon_{r}$, favoring a high pyroelectric coefficient p and a low permittivity $\varepsilon_{r}$ and the other for detectivity $F_{D}=p/\sqrt{{\varepsilon_{0}\varepsilon }_{r}\tan\delta }$, which, in addition, requires a low tan⁡δ. Figure 23 depicts the voltage figure of merit $F_{v}=p/\varepsilon_{r}$ versus poling temperature (poled at 250 kV/cm) of (111) and (100) oriented PZT 15/85 thin films.

**Figure 24:**
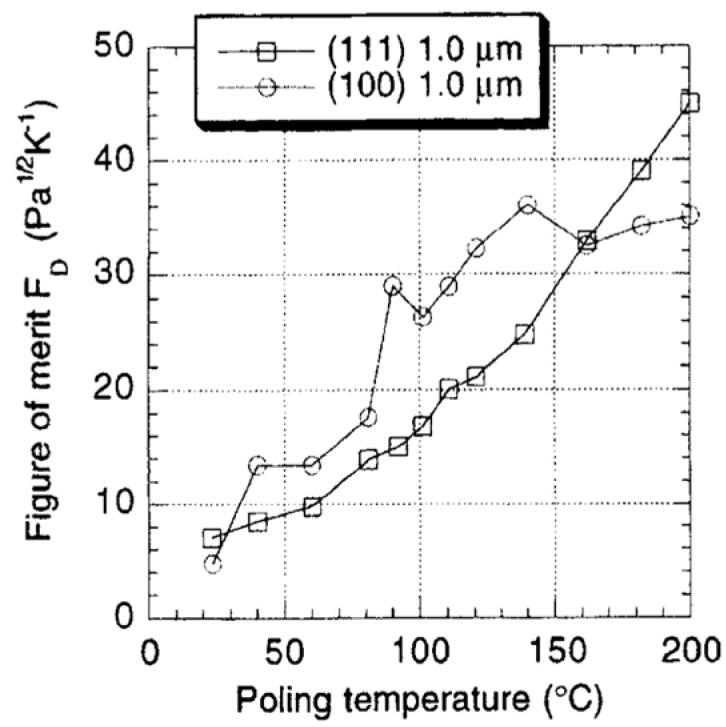
Detectivity ($F_{D}$) versus poling temperature (poled at 250 kV/cm) of (111) and (100) oriented PZT 15/85 thin films [56]. Reprinted from [56], Figure 1, Figure 3, Figure 4 and Figure 5, with permission from the Copyright Clearance Center Inc (CCC) on Taylor and Francis’s behalf.

Optimization of the poling temperature and the electrical field leads to ~40% improvement, as shown in the figure of merit for voltage and detectivity. Due to a significant reduction in the dielectric constant of porous films, values of Fv are 3X higher in PCT than in PZT thin films, which suggests that PCT is an excellent candidate for pyroelectric applications. Figure 24 shows that detectivity ($F_{D}$) versus poling temperature (poled at 250 kV/cm) of (111) and (100) oriented PZT 15/85 thin films 180 domains contribute to phase instability. Thermal activation helps to avoid obtaining such domain configuration.

**Figure 25:**
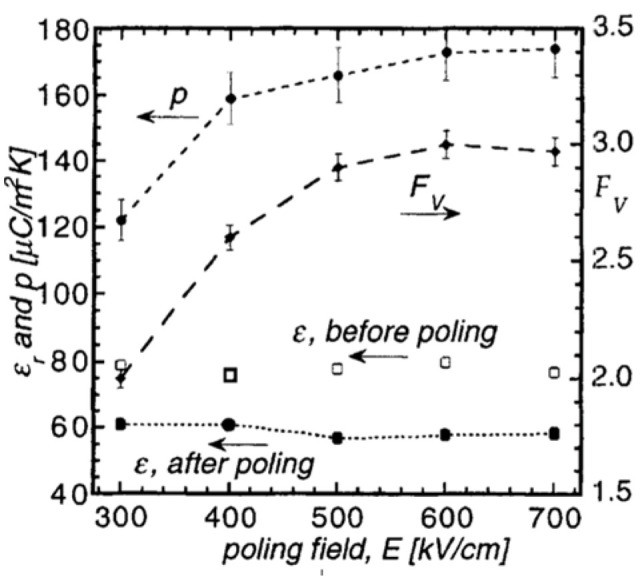
Development of $p,\;\varepsilon_{r}$, and $F_{v}$ (with same units as p) for poling at 170 °C/10 min as a function of electric field for PCT15 films [56]. Reprinted from [56], Figure 1, Figure 3, Figure 4 and Figure 5, with permission from the Copyright Clearance Center Inc (CCC) on Taylor and Francis’s behalf.

Figure 25 depicts the changes of the pyroelectric coefficient, the permittivity and the figure corresponding to voltage for the PCT15 as a function of poling electric field. Poling was performed at 170 °C for 10 min. Pyroelectric coefficient is seen to steadily increase, reaching a plateau at about 600 kV/cm, which can be attributed to the continued switching of 90°- and/or 180°-domains. The permittivity measured after poling remains approximately constant for all electric fields; thus, $F_{v}$ remains constant when p changes. The necessary poling fields are high compared to PZT, which may be due to the small grain size of about 55 nm for porous PCT15. Domain wall motion and thus polarizability is known to be significantly reduced in small-grained material [55,58]. The dielectric breakdown strength for films of all compositions was equally high and occurred after about 5 min at 160 °C between 1 and 1.5 MV/cm.

Hot poling reduces a-domain population in <100> oriented PZT thin films. At high temperatures, the mobility of oxygen vacancies increases such that it can migrate even under small electric fields. The oxygen vacancies combine with lead vacancies as well as other impurities like ${Na}^{+},{Cu}_{2}^{+},{Fe}_{2}^{+}$, and ${Fe}_{3}^{+}$ to form defect dipoles that interact with the domain walls [59]. The defect-dipole complexes reorient themselves under an applied field [60].

The FE domain configuration is frozen during cool-down to room temperature and the polarization is stabilized. The domain pinning by defects leads to a large internal field of 140 to 180 kV/cm (imprint) [56,61]. After poling, some of the poled domains may switch back due to phase instability. Back switching reduces the polarization, and as a result, the pyroelectric signal decreases with time. High coercive field materials with good retention are thus better candidates for pyroelectric applications. Interestingly, these materials also offer comparatively lower dielectric constants.

The decrease in the $\varepsilon_{r}$ and tan⁡δ after the poling process is attributed to the elimination of domain walls by the internal field (Figure 25). As the DC-poling field increases, the dependence of $\varepsilon_{r}$ and tan⁡δ on the AC-field drops compared to the unpoled state. This is because domain walls are removed. Hence, the wall contribution diminishes. The combination of these phenomena with porosity and an increased pyroelectric coefficient from Ca-addition result in an improved figure of merit.

### 2.7. Pyroelectric Measurement

It has already been shown that temperature changes in some crystals (e.g., perovskites) modify the magnitude of their permanent dipole moments or their polarization. This phenomenon is referred to as “pyroelectricity”, which may arise from one or more of the following sources [62]:

- Changes in the permanent polarization along the x direction $\left(∆P_{X0}\right)$,
- Changes in dielectric permittivity of medium leading to the electric field induced polarization $\left(∆\varepsilon_{Xk}\right)$,
- Changes in the piezoelectricity $\left[∆\left(d_{Xkl}\right)\right]$, and
- Changes in the flexoelectric polarization $∆\left(\mu_{Xijk}\frac{{\delta \mu }_{jk}}{\delta_{i}}\right)$.

Therefore, the changes in the polarization along x axis is described by:

(8) $$∆P_{x}=∆P_{x0}+∆\varepsilon_{xk}E_{k}+∆\left(d_{xkl}\sigma_{kl}\right)+∆\left(\mu_{xijk}\frac{{\delta \mu }_{jk}}{\delta_{i}}\right),$$

where $P_{x0}$ is the permanent polarization along x axis, $\varepsilon_{xk}$ represents components of the dielectric tensor, $E_{k}$ represents the electric fields in the *k* directions, $d_{xkl}$ represents components of the piezoelectric tensor, $\sigma_{kl}$ represents components of the stress tensor, $\mu_{xijk}$ represents the components of the flexoelectric tensor, and $\mu_{jk}$ represents the components of the strain tensor. This 1D analysis can be generalized to 2D or 3D without any loss of generality. By dividing both sides by incremental changes in temperature (ΔT) and considering the limiting case, where ΔT⟶0, the following equation is obtained:

(9) $$\frac{\partial P_{x}}{\partial T}=\frac{\partial P_{x0}}{\partial T}+\varepsilon_{0}E_{k}\frac{\partial \varepsilon_{xk}}{\partial T}+\frac{\partial \left(d_{xkl}\sigma_{kl}\right)}{\partial T}+\frac{\partial \left(\mu_{xijk}\frac{{\delta \mu }_{jk}}{\delta_{i}}\right)}{\partial T},$$

Thus, the pyroelectric coefficient is calculated as follows:

(10) $$\alpha_{x}=P_{x}+\varepsilon_{0}E_{k}\frac{\partial \varepsilon_{xk}}{\partial T}+\frac{\partial \left(d_{xkl}\sigma_{kl}\right)}{\partial T}+\frac{\partial \left(\mu_{xijk}\frac{{\delta \mu }_{jk}}{\delta_{i}}\right)}{\partial T},$$

where $P_{x}=\frac{\partial \varepsilon_{xk}}{\partial T}$. When a field is applied externally, the second term in (10), may become enormously large in the vicinity of phase transitions, where $\frac{\partial \varepsilon_{xk}}{\partial T}$ becomes very large [63].

This criterion is implemented for the device operation in the “dielectric bolometer mode”. It must be noted that as the electric field increases, $\frac{\partial \varepsilon_{xk}}{\partial T}$ decreases, and thus, the second term in (10) remains finite. Nevertheless, it is undesirable for pyroelectric detection because it obscures the pyroelectric effect. The effect of the flexoelectric term, although considerable at the nanoscale [64], where large strain gradients are present, is neglected in the present treatment.

#### 2.7.1. Measurement of the Pyroelectric Effect

There are three main approaches to the measurement of the pyroelectric effects on bulk samples: (1) direct measurement of the polarization and/or dielectric constant at two or more fixed temperatures, (2) measurement of the pyroelectric current induced by a ramping temperature up (or down), and (3) measurement of the pyroelectric current induced by periodic temperature change. These are briefly discussed below:

##### Direct Measurement of the Polarization and/or Dielectric Constant at Two or More Fixed Temperatures

A.1. Sawyer-Tower (ST) Bridge Technique

**Figure 26:**
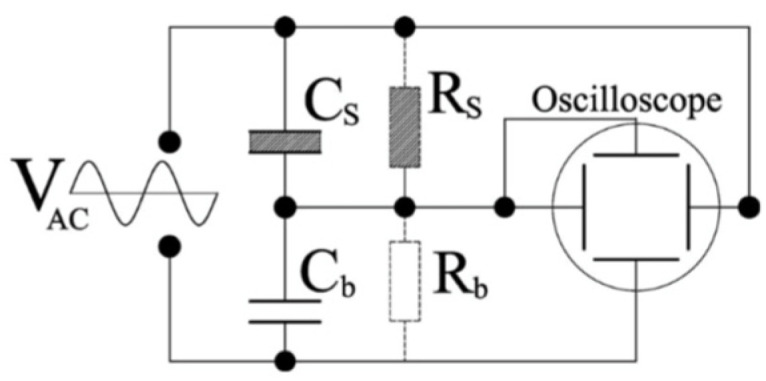
The Sawyer-Tower bridge. In its original form, it did not consider the presence of the leakage current represented here by $R_{s}$. This current must be partially balanced by a shunt resistor $R_{b}$ and polarization hysteresis curve must be checked. A properly balanced $R_{s}$ would show a square-shape polarization hysteresis loop. The capacitance of the material is denoted by $C_{s}$. The bridge is constructed using a linear capacitor $C_{b}$ [62]. Reprinted from [62], Figure 1, Figure 2, Figure 3, Figure 4, Figure 5, Figure 6, Figure 7, Figure 8 and Figure 9, with the permission of AIP Publishing.

Ferroelectrics can reverse the direction of their spontaneous polarization under the influence of an external electric field and hence allow the direct measurement of the magnitude of the permanent dipole moment. In the ST technique [65,66], the polarization and/or dielectric constant of the material is directly measured at two or more fixed temperatures, and the pyroelectric coefficient is obtained from $P_{x}=\partial P_{x0}/\partial T$. The method is restricted to ferroelectrics, where coercive fields are much smaller than their breakdown fields. The currents are evaluated by running the ST bridge as shown in Figure 26. 

There are three main approaches to the measurement of the pyroelectric effects on bulk samples.

The analysis above assumes that polarization (P) is perpendicular to the plane of the contacts. If P makes an angle Ω with respect to the to the plane of the contacts, this technique provides the projection of the polarization on the plane of the contacts, Pcos⁡Ω. The bridge is generally constructed using a linear capacitor ($C_{b}$), where $C_{b}\gg C_{s}$ and $C_{s}$ is the capacitance of the FE material, which includes polarization switching $A_{s}\cdot P_{0}$. Here, the sample area is denoted by $A_{s}$. The voltage measured across $C_{b}$ is proportional to the total surface charge on $C_{s}$, which represents the polarization charge stored on the surface of the FE material in the absence of a real electric current in the FE medium. Most FE media exhibit ohmic conductivity and/or charge trapping effects. The Ohmic conductivity can be partially compensated for by shunting $C_{b}$ by resistor, $R_{b}$ whose value is varied until an ideal square polarization is achieved.

P(E) exhibits an ‘S’ shape rather than a rectangular contour if the coercive field is not uniform over the volume of the medium under investigation. It is then impossible to distinguish between Ohmic conductivity and true polarization effects. Because no value of the shunting resistor exists for which the hysteresis loop becomes rectangular. Then the ST bridge, using a shunting resistor, becomes unreliable for accurate determination of the polarization. This reservation also applies to a variation of the ST bridge, in which $C_{b}$ is replaced by a current-to-voltage converter, whose output is either electrically or “computationally” integrated and allows for extraction of the polarization and coercive field; this has become a popular ST bridge technique in industry [67].

A second issue associated with ST bridge measurement is due to charge trapping at the contact-sample interface or, sometimes, in the bulk of the medium and leads to real current components. In this case, no technique based on measuring the reversible charge storage using an alternating electric field will produce meaningful data (an example of such FE-like behavior is electret [68]. The ST technique is prone to inaccuracies stemming from the Ohmic conductivity as well as noise currents caused by interface/bulk trapped charges and is appropriate only for ideal pyroelectric materials with square hysteresis loop.

A.2. Modified ST bridge (Liu) method

This method assumes that the loss (trap) current ($I^{T}$) is a function of the applied voltage and only the displacement current is proportional to $r_{v}=\partial V/\partial t$. Liu [69] proposed a similar method that allows the extraction of the polarization and coercive field. The total polarization P* is the sum of the permanent polarization $P_{x}$, and the polarization induced by the electric field εV/d, where d is the sample thickness.

**Figure 27:**
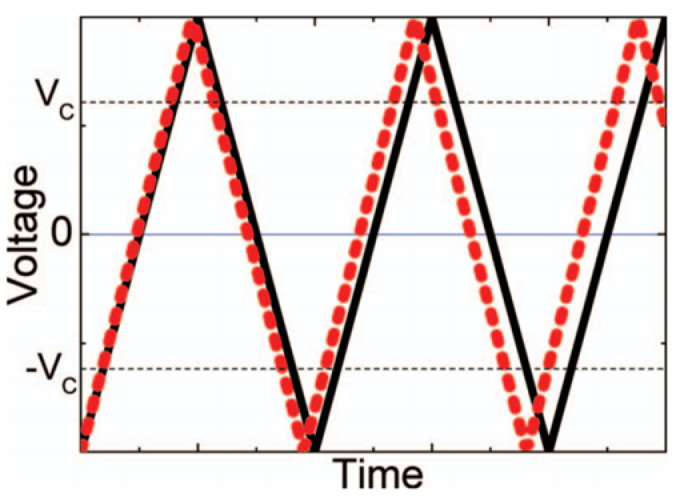
Two saw-tooth voltage-time profiles with frequencies differing by 5% [62]. Reprinted from [62], Figure 1, Figure 2, Figure 3, Figure 4, Figure 8 and Figure 9, with the permission of AIP Publishing.

In this method, the currents are evaluated by running the ST bridge with a triangular-shaped voltage input at two closely spaced frequencies. To ensure that complete switching of the polarization takes place between applied voltages, amplitudes of the two input voltages chosen are larger than Vc. (Figure 27).

By integrating the current difference at the same voltage of the two voltage wave forms, it can be shown that:

(11) $$P^{*}\left(V\right)=\int_{V_{1}}^{V_{2}}\frac{I^{T}}{A\cdot ∆\Gamma_{V}}dV.$$

Equation (11) is useful when polarization switching, rather than the externally applied voltage, dominates the current. This condition is fulfilled when the coercive field is small. By calculating $P^{*}\left(V\right)$ at two different temperatures, one can evaluate the sum $\left(P_{x}+\varepsilon_{0}E_{k}\frac{\partial \varepsilon_{xk}}{\partial T}\right)$.

Furthermore, $P_{x}$ is determined by evaluating $P^{*}$ at V=0.

Another technique is based on the use of an AC bridge to measure C(V); then, $P^{*}(V)$ is written as:

(12) $$P^{*}\left(V\right)=\int_{V_{1}}^{V_{2}}C(V)dV.$$

The measurement of $P^{*}\left(V\right)$ provides a simple means to verify the presence of ferroelectricity in bulk materials since its value is independent of frequency up to the GHz range, at least in the case of “ideal” FE material. The drawback to capacitance measurement by an AC bridge is that it always comprises artifact components due to displacement, ohmic, and charge-injection currents. The latter not only contributes to the inaccuracy of the capacitance measurements but also affects the electric field in the sample.

##### Measurement of the Pyroelectric Current Induced by Ramping Temperature Up (or Down)

The continuous temperature ramp technique measures the current flowing between two contacts on a pyroelectric sample induced by continuous heating or cooling. Although the thermal diffusion coefficient ($D_{th}$) for most pyroelectric materials is quite low (0.01–0.1 cm^2^/s), even very rapid heating rates ($R_{th}$~1°K/s) does not lead to inhomogeneous heating of the unclamped samples. Thus, the sample is uniformly heated and hence, only the first two terms in (10) will contribute to the change of polarization. The effective pyroelectric coefficient is obtained by using two measurement techniques: (1) measuring voltage as suggested by Lang and Steckel [70], and (2) measuring the current flowing under short circuit conditions according to Glass [71] and Byer and Roundy [72].

B.1. Measurement of the pyroelectric voltage developed across the film by ramping temperature up (or down):

Lang and Steckel [70] described the following method for measuring the pyroelectric coefficient, DC dielectric constant, and volume resistivity of lead-zirconate-titanate ceramic over a wide temperature range. The technique is based on the observation of the capacitive charging of a pyroelectric sample by the pyroelectric current generated during a continuous temperature change. 

**Figure 28:**
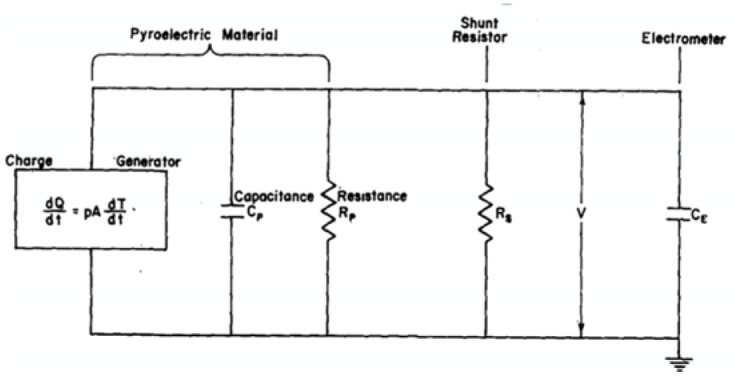
Lang and Steckel measurement circuit [70]. Reprinted from [70], Figure 1, with the permission of AIP Publishing.

Figure 28 depicts the equivalent circuit for the pyroelectric device, along with its measurement circuit. Assuming that the polar axis is normal to the sample electrodes, the method then leads to $p,\;{\;C}_{p}$, and $R_{p}$ being parallel to the polar axis. The circuit of Figure 28 is shown below:

(13) $$\left(PA/C_{T}\right)\left(dT/dt\right)=dV/dt+\left(1/R_{T}C_{T}\right)V,$$

(14) $$C_{T}=C_{P}+C_{E},$$

(15) $$R_{T}^{-1}=R_{p}^{-1}+R_{S}^{-1},$$

where $C_{T}$ is the equivalent (total) capacitance of the pyroelectric element $C_{P}$ and the electrometer $C_{E}$, and $R_{T}$ is the equivalent (total) resistance of pyroelectric element $R_{P}$, and the shunt resistance $R_{S}$ (external to sample).

Assuming that the time constant characterizing the rate of temperature change of the samples is much greater than $R_{T}C_{T}$, the integration of (13) with the initial condition $V\left(0\right)=0$ gives:

(16) $$V=PAR_{T}{\left(dT/dt\right)}_{0}\left[1-e^{-\frac{1}{R_{T}C_{T}}}\right],$$

Here, ${\left(dT/dt\right)}_{0}$ represents the time derivative of the temperature at t=0. The method is easily realizable using a temperature-controlled table. Since the sample temperature is directly measured, P can be determined for uniformly heated samples by measuring $I_{P}\left(T\right)$ with at least two different shunt resistors $\left(R_{S}\right)$. This technique does not require measurement of heat flux that does not attain isothermal conditions and does not alter the domain structure of a FE material. However, it does require prior knowledge of conductivity as a function of temperature and is a suitable alternative when $R_{ammeter}\ll R_{S}$ does not hold. 

##### Measurement of the Short Circuit Current Produced by a Continuous Temperature Ramping (Up or Down)

**Figure 29:**
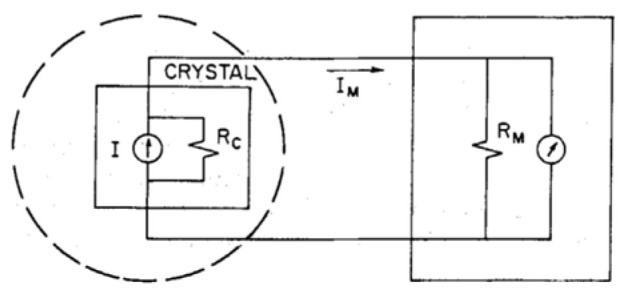
Measurement apparatus equivalent circuit [72]. Reprinted from [72], Figure 1, with permission from the Copyright Clearance Center Inc (CCC) on Taylor and Francis’s behalf.

Figure 29 shows the equivalent circuit for DUT that is connected to an ammeter, which was used in [70,71,72] to directly measure pyroelectric current from

(17) $$I_{P}=AP\left(T\right)dT/dt,$$

where $P\left(T\right)=dP/dt$ is the pyroelectric coefficient, P is the polarization, $R_{C}$ is the crystal leakage resistance, and $R_{M}$ is the ammeter input resistance. The measured current is given by:

(18) $$I_{M}=\frac{R_{C}}{R_{C}+R_{M}}I.$$

As long as $R_{M}\gg R_{C}$, $I_{M}$ gives an accurate estimate of the pyroelectric current (denoted by I in Figure 29).

##### Temperature Oscillation (Dynamic) Method

**Figure 30:**
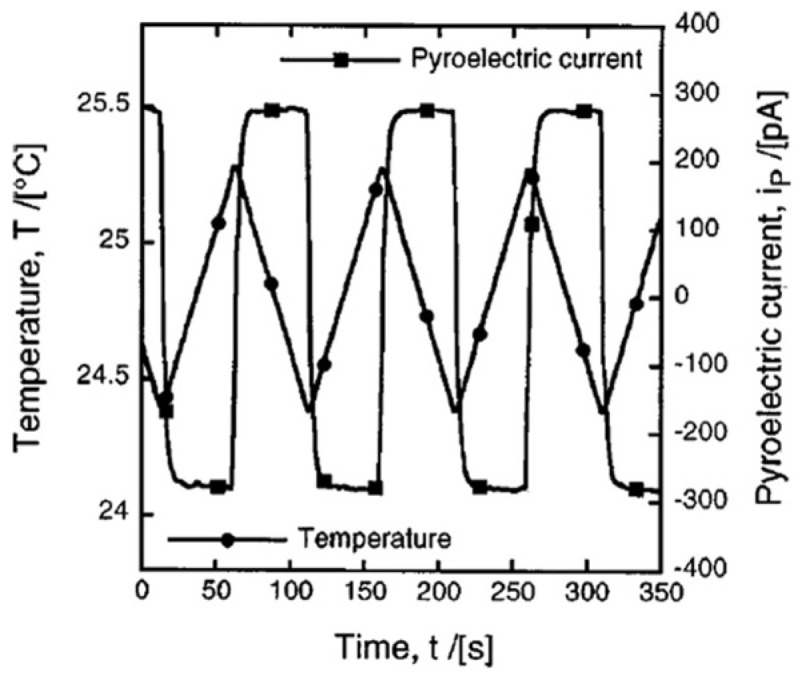
An example of the dynamic time ramping technique used to measure the pyroelectric coefficient of a $67\%\left({Mg}_{1/3}{Nb}_{2/3}\right)O_{3}-33\%\;PbTiO_{3}$ single crystal (a Peltier element run at 1 mHz during a 16 min 40 s period was used to create the saw-tooth temperature profile with an amplitude of 1 degree) [62]. Reprinted from [62], Figure 1, Figure 2, Figure 3, Figure 4, Figure 8 and Figure 9, with the permission of AIP Publishing.

This method was utilized by Davis et al. [73] to measure the pyroelectric coefficient of a thin- plate specimen using the apparatus described by Daglish [74]. The sample temperature is cycled at a frequency of 10 mHz over 1 °C. Using a Peltier element, a triangular temperature wave is formed. The current between the two electrodes is then recorded and the value of p is calculated via (27). A typical temperature waveform as well as resultant pyroelectric current is shown in Figure 30. This technique is simple and direct and does not require prior knowledge of conductivity as a function of temperature. The sample is poled once and then, a series of limited temperature runs is performed at T < Tc. Repeated oscillations over a finite temperature range will eventually deplete the traps [73].

Since $\varepsilon_{0}E_{k}\left(\frac{\partial \varepsilon_{xt}}{\partial T}\right)\left|sckt\right.\approx 0$, the primary unclamped pyroelectric coefficient is obtained from (10) (see Appendix A.3). However, following issues remain:

1. Creation and measurement of a large (dT/dt) with low noise.
2. Thermoelectric effect is due to contacts which contribute to measured current.
3. Thermally activated current due to non-uniformly distributed charged traps [75] is indistinguishable from $I_{xp}$ and may dominate, especially in the poled ferroelectrics. Depletion of the traps requires a large number of repetitions (e.g., in electrets).
4. Very long trap time response.
5. Measurements of the current through a short circuit require $R_{ammeter}\ll R_{S}$ (for all Ts).

In case of thin films, the following obstacles further complicate the measurement of pyroelectric coefficient:

1. Depending on their preparation details, thin films generally contain pinholes which decrease the “apparent” resistivity of the sample.
2. When the trap concentration $N_{t(thin\;film)}\gg N_{t(bulk)}$, where $N_{t(bulk)}$ is the trap concentration in the bulk, it is possible that $I^{t}>i_{P}$, where $i_{P}$ is the pyroelectric current.
3. It is possible that the current due to the thermoelectric effect $i_{th-el}\gg i_{P}$.

Periodic temperature change techniques can be divided into two categories:

1. Periodic pulse techniques, and
2. The continuous oscillation method.

C.1. Temperature Changes Using Periodic Pulse Techniques

**Figure 31:**
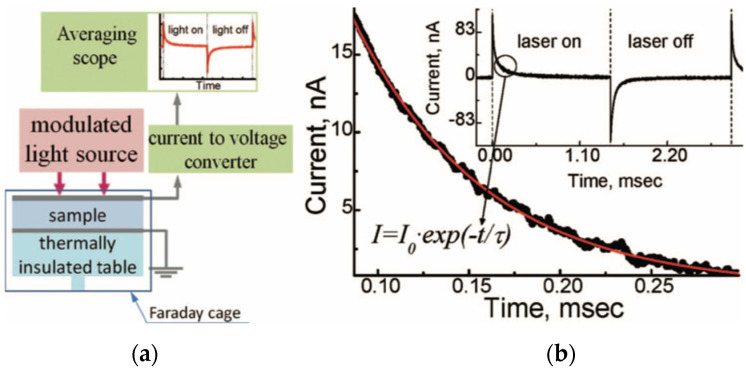
(**a**) Scheme of the set-up for the basic Chynoweth method, and (**b**) an example of the pyroelectric current measured with the Chynoweth technique. The sample is 0.2 × 0.2 μm wide, 0.4 μm thick self-supported film of BaTi$O_{3}$ tethered to a Si substrate [62]. The top and the bottom contacts are of Ag and account for more than 90% of the heat loss. The inset shows a full period (laser on and off, 666 Hz); the main panel shows an exponential fit. The film was irradiated by an IR laser (λ = 1310 nm) with a flux of 3 mW/mm^2^. Note: for a self-supported film, a lump model is applicable, and the heating and the cooling processes are fully symmetric [62]. Reprinted from [62], Figure 1, Figure 2, Figure 3, Figure 4, Figure 8 and Figure 9, with the permission of AIP Publishing.

Chynoweth [76] used a periodic step-like heating from a modulated IR laser, which was focused on the thermally insulated sample. A schematic of the instrumentation is given in Figure 31a.

**Figure 32:**
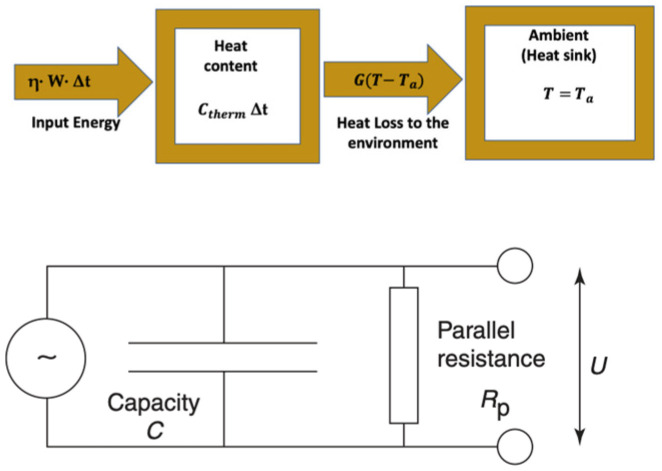
Lump model for a pyroelectric IR detector: schematic diagram of the thermal model and electrical equivalent circuit of a pyroelectric detector. Upon absorption of fraction η of IR radiation (power W), the sensor temperature increases by ΔT, which may be obtained from the heat flow balance. The temperature change for a given amount of heat input η·W·Δt depends on the heat conductivity $G_{th}$ to the surrounding environment assumed at temperature $T_{0}$, the heat capacity $C_{th}$ of the element. For a thin film structure uniformly illuminated by the IR radiation, it is generally safe to neglect thermal wavelength effects and assume that the element retains a uniform temperature. Reprinted from [77], Figure 5, with permission from Elsevier.

Large pyroelectric currents in response to very small temperature variations is possible if a modulated IR laser is employed as the heating source since, in this case, warm-up and cool-down rates can be much larger than the those in temperature ramping techniques. This technique relies on the following two assumptions:

1. A lump model can be devised for the DUT (Figure 32). Accurate prediction of the pyroelectric device behavior under broad range of frequencies requires distributed parameter modeling of the thermal gradients. However, if temperature differences within the DUT are much smaller than the temperature changes induced by the input energy η·W·∆t (where, η is the fraction of absorbed IR power, W is the input power, and ∆t is the duration period), a reasonably accurate lumped parameter modelling approach is also possible to create. This requires identification of the thermal capacitance $C_{th}$ and heat conductance to the environment $G_{th}$ for the DUT if temperature differences within the DUT are much smaller than the temperature changes induced by the input energy. Otherwise, one can introduce “pseudo-lumped” parameters if the temperature rise and fall obey the following:
  (19) $$\tau_{th}=C_{th}/G_{th}\;,$$
  (20) $$T\left(t\right)=T_{max}\left[1-\exp\left(-t/\tau_{th}\right)\right]\;.$$

Here, $\tau_{th}$ is the characteristic thermal time constant, and the maximum temperature is $T_{max}=F/G_{th}$, where F=η·W/A, with A= detector area in the input flux [62].

2. The recording of the pyroelectric current transients and averaging of the output signal is possible over many cycles to improve the signal to noise ratio (SNR).

As schematically shown in Figure 31, the modulated IR laser capable of providing a few mW of power is directed onto the sample. The pyroelectric current generated from the sample is then fed to a current-to-voltage converter and the voltage is recorded using an averaging oscilloscope. Chynoweth [76] modeled DUT as a homogenously heated body with a heat capacitance, connected to its surroundings via $G_{th}$. As seen in Figure 31b, the pyroelectric current decays exponentially with time and the response to “radiation on” and “radiation off” is symmetric. $\tau_{th}$ is determined from the $i_{P}$ decay wave form. If voltage is measured, both the $\tau_{th}$ and the electrical time constant $\tau_{el}$ are obtained and if $C_{th}$ is known, the only unknown parameter is the amount of radiation being absorbed by the sample.

For bulk samples both the measurement of the $C_{th}$ and the derivation of the pyroelectric current ($I_{P}$) generated in response to the periodic heating is relatively simple. For the case of thin films, P is found from an analytical solution. With this method, electromechanical isolation of the sample from noise is feasible. Thus, the sensitivity is improved. Also, the effect of trapped charges is detectable [62].

##### Temperature Change Using Continuous Oscillation Method

**Figure 33:**
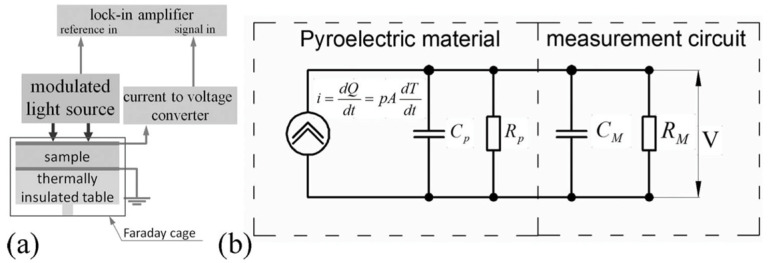
(**a**) The instrumentation diagram for the continuous temperature oscillation technique, and (**b**) the equivalent electrical circuit with the pyroelectric material and a high-impedance voltmeter connected in parallel [62]. Reprinted from [62], Figure 1, Figure 2, Figure 3, Figure 4, Figure 8 and Figure 9, with the permission of AIP Publishing.

In this technique, the sample is subjected to a continuous, sinusoidally modulated heat source (typically a modulated laser), and the generated current or voltage is recorded with a phase sensitive device (typically a lock-in amplifier). The lock-in technique provides a good signal to noise (S/N) ratio for a single frequency component (Figure 33).

If the sample can be modeled by the lumped parameter approach, the derivation of the current generated in response to the periodic heat input is relatively simple. However, for the case of thin films or multiple layer structures, the lumped parameter model is not always adequate. Holeman [78] proposed models applicable to thin films for a number of practical cases.

D.1. Extended version of continuous oscillation technique

For the cases in which the material’s conductivity is known, Sharp and Garn [79] and Whatmore [80] proposed to excite the sample by a sinusoidal thermal wave with a frequency low enough to insure homogeneous heating of the DUT (typically 0.2–0.02 Hz). Then, the pyroelectric component of the current will have a 90° phase difference with respect to the sinusoidal thermal wave input. This allows the accurate measurement of the pyroelectric effect in the presence of finite Ohmic losses and thermally stimulated current (TSC) due to the release of trapped charges. This technique requires $R_{ammeter}\ll R_{S}$ (for all Ts). However, for thin films or multiple layer structures [81], the lumped parameter model is not always adequate.

D.2. Temperature change using continuous oscillation method as applied to substrate supported thin films

There are two fundamentally different approaches to measuring the pyroelectric coefficient of thin films on substrates.

D.2.1. Applying Bulk Techniques to Measure Pyroelectric Coefficient Using the Continuous Oscillation Method

When using periodic temperature change techniques, one has to ensure that the film is uniformly heated and cooled. The first approach of measuring p is to heat and cool the substrate, ignoring the presence of the film. The fact that the thermal capacitance of the substrate $C_{th(sub)}$ is much larger than that of the film $C_{th(film)}$ ensures that the film is heated homogenously throughout its volume. This criterion is satisfied when the modulation frequency $f_{mod}$ is chosen such that $f_{mod}\ll 1/\tau_{D(film)}$, where $\tau_{D(film)}$ is the thermal diffusion time of the film. In this case, the film and the substrate may be treated as a single body during heat-up and cool-down process and any of the temperature change techniques using continuous oscillation method already discussed above is applicable.

D.2.2. Holeman Periodic Temperature Change Method

The second approach is to use the Holeman methods [62,78]. Holeman has shown that as the modulation frequency increases, the pyroelectric response eventually depends only on the properties of the film and not of the substrate. This occurs if the $f_{mod}\gg 1/\tau_{D(film)}$ modulation frequency is at least one order of magnitude higher than the reciprocal of the thermal diffusion time through the film. However, it is necessary to know the thermal properties of the film. $\tau_{D(film)}$ is found by plotting the pyroelectric response vs. frequency. The $I_{P}$ measured under short circuit conditions $I_{p,sckt}$ increases with f until $f_{mod}\gg 1/\tau_{D(film)}$. This corresponds to the transition from homogeneous heating of the film (low frequency) to heating of only the top layer of the film (high frequency). Above this transition frequency, the current becomes independent of frequency. However, $C_{th(film)}$ is needed in order to calculate the p. For very thin films, $\tau_{D(film)}$ may easily be so short as to require $f_{mod}\gg 1$ mHz, thereby making the periodic temperature change technique impractical. This subject is further discussed below:

Researchers in [62,78] noted that using complex notation to represent T(x,t) can greatly simplify the mathematical modeling of the relevant heat transfer problem. A “complex” temperature term implies that the absolute value of temperature $\left(\left|T\;(x,\;t)\;\right|\right)$ oscillates with frequency ω. Researchers in [62,78] developed models for several cases of practical interest and showed that if a thermal detector could be modeled via a lump modeling approach and heated by a sinusoidally modulated heat wave with a modulation frequency $f_{mod}$ at a flux (F), the time-dependent portion of temperature varies with frequency as:

(21) $$T\left(\omega \right)=\frac{F}{G_{th}}\left(\frac{1}{\sqrt{1+w^{2}\tau_{th}^{2}}}\right),$$

where $\tau_{th}=c_{th}/G_{th}$ is the thermal time constant. The pyroelectric current is found from (27):

(22) $$I_{P}=\frac{AP\omega F}{G_{th}\sqrt{1+w^{2}\tau_{th}^{2}}}.$$

Thus, at low modulation frequencies $\left(w^{2}\tau_{th}^{2}\ll 1\right)$, the pyroelectric current increases linearly with $\omega =2\pi f_{mod}$ as:

(23) $$I_{low}=APF\omega /G_{th}.$$

This corresponds to a homogeneous heating regime, where both the substrate and the film may be treated as a single entity. As $f_{mod}$ is further increased to at least one order of magnitude higher than the reciprocal of the thermal diffusion time through the film, $I_{low}$ begins to saturate as follows:

(24) $$I_{low}=APF/C_{therm}.$$

**Figure 34:**
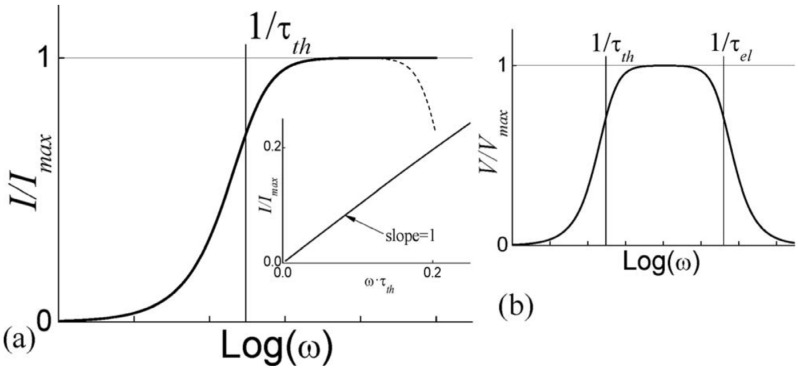
(**a**) Typical frequency dependence of pyroelectric current generated in response to sinusoidally modulated heating when the lumped model is applicable; the bold line shows the idealized case when a current-to-voltage converter has a very small impedance over all frequency ranges. At sufficiently high frequencies $\left(\omega^{2}\tau_{th}^{2}\gg 1\right)$, the pyroelectric current remains constant $I_{max}=A\cdot P\cdot F/G_{th}$. In practical measurements, above some frequency, the input impedance of the sample decreases sufficiently to cause a decrease in the measured current (shown as a dashed line). The actual pyroelectric currents are known to remain constant into the nanosecond time scale. The inset shows that at very low frequencies, the current is directly proportional to frequency. (**b**) Typical frequency dependence of the pyroelectric voltage. [62]. Reprinted from [62], Figure 1, Figure 2, Figure 3, Figure 4, Figure 8 and Figure 9, with the permission of AIP Publishing.

This corresponds to the non-homogenous heating regime, where the response is affected only by the film (and is independent of substrate) properties. Therefore, if either $C_{th}$ or $G_{th}$ is known for the film, its pyroelectric coefficient can be extracted. In practice, as frequency further increases, the input impedance of the sample reduces while the impedance of the current-to-voltage converter remains relatively constant. Thus, at a sufficiently high frequency, the current flowing through the sample will dominate the total current, causing an apparent decrease in the pyroelectric current flowing through the current-to-voltage converter in Figure 34.

For very thin films, the thermal diffusion time can easily become so short as to require modulation frequencies in the MHz range, thereby making the periodic temperature change technique impractical. If the measurement is performed with the finite impedance of an equivalent resistor (R) and equivalent capacitor (C) as shown in Figure 33, then the voltage is given by:

(25) $$V=I\left|Z\right|=I\frac{R}{\sqrt{1+\omega^{2}\tau_{e}^{2}}}=A\alpha \frac{F}{G_{th}}\frac{\omega }{\sqrt{1+\omega^{2}\tau_{th}^{2}}}\frac{R}{\sqrt{1+\omega^{2}\tau_{e}^{2}}},$$

where $\tau_{e}=RC$ is the electrical time constant. The equivalent resistance is given by $R=R_{P}R_{M}/\left(R_{P}+R_{M}\right)$ and the equivalent capacitance $C=C_{P}+C_{M}$. If $\tau_{e}\ll \tau_{th}$, the voltage increases linearly with frequency and then saturates at a value shown in Figure 34b:

(26) $$V_{max}=APFR/G,$$

for $1/\tau_{th}<f_{mod}<1/\tau_{e}$. Since R is not known, a comparison of the values of $V_{max}$ measured with different values of $R_{S}$ is needed to deduce P.

This method has been extended by Sharp-Garn to separate various thermally stimulated relaxation processes from the pyroelectric current [79,82]. By differentiating Equation (21), it is realized that for $\omega \cdot \tau_{e}<0.1$, the pyroelectric current is 90° out of phase with respect to the input sinusoidal heat wave. On the other hand, as long as the temperature range is limited to less than 0.5°K, thermally stimulated current arising from various relaxation processes can be approximated as:

(27) $$i_{TSC}=i_{TSC0}+TB_{T}.$$

Here, $i_{TSC0}$ is the temperature independent term, and $B_{T}$ is a constant that depends on the nature of the thermal traps. If the sample is heated at a constant rate b, superimposed on a sinusoidally modulated component:

(28) $$T=T_{0}+bt+T_{1}\sin\left(\omega t\right).$$

Then the non-periodic component of the induced current is given by

(29) $$i_{DC}=i_{TSC0}+B_{T}\left(T_{0}+bt\right)+PAb,$$

and the periodic part of the current is given by

(30) $$i_{AC}=B_{T}T_{1}\sin\left(\omega t\right)+PAT_{1}\omega \cos\left(\omega t\right).$$

Thus, by measuring $i_{AC}$, the component that is out of phase with respect to the temperature, p is extracted. Practical applications of this method are described in [79,80].

##### Pyroelectric Current Generated in a Film Supported by a Substrate (Heat Sink) in Response to Sinusoidally Modulated, Uniform, and Lateral Heating

This considers the one-dimensional temperature distribution of a dielectric film of thickness d, which is supported by a thermally conductive substrate whose temperature is assumed to be $T_{sub}=0$ and assumes that the whole system is being heated by a sinusoidally modulated heat wave of the form $F_{d}\left[1+\exp\left(j\omega t\right)\right]$. Furthermore, assuming heat losses through radiative and convective processes can be neglected and thus, the heat loss takes place only through the substrate. Holeman [78] showed that:

(31) $$T\left(x,t\right)=\frac{F_{d}}{G_{th}}\left[\left(\frac{\tanh\left(\omega d\right)\cosh\left(\omega x\right)}{\omega }-\frac{\sinh\left(\omega x\right)}{\omega }\right)\exp\left(j\omega t\right)+\left(d-x\right)\right],$$

where $\omega =\left(1+j\right)\sqrt{\omega /2D}$ is an inverse characteristic length. 

Proceeding the method outlined in [62,78], it can be noted that $I_{P}$ has a low frequency $\left(\omega \tau_{th}<1\right)$ asymptote given by:

(32) $$\left|I_{low}\left(\omega \right)\right|=\frac{AP\left(F_{d}\right)\tau_{th}}{2dC_{th}}\omega =\frac{AP\left(F_{d}\right)d}{2G_{th}}\omega ,$$

where $\tau_{th}=G_{th}/C_{th}$ is the thermal time constant of the film, $C_{th}$ is the thermal capacitance, $G_{th}$ is the thermal conductivity and d is the thickness of the film. Thus, if either the thermal capacitance $C_{th}$ or thermal conductivity $G_{th}$ is known, the slope of the plot $\left|I_{low}\left(\omega \right)\right|$ vs. ω can be utilized to extract the pyroelectric coefficient.

Also, the high frequency $\left(\omega \cdot \tau_{th}>3\right)$ asymptote of $I_{P}\;$becomes independent of frequency, as shown in [62,78]:

(33) $$\left|V_{high}\right|=\frac{AP\left(F_{d}\right)}{dC_{th}},$$

(34) $$V_{high}=I_{high}\left|Z\right|=I\frac{R}{\sqrt{1+\omega^{2}\tau_{e}^{2}}}=AP\frac{F_{d}}{dC_{th}}\frac{R}{\sqrt{1+\omega^{2}\tau_{e}^{2}}},$$

where R is the resistance of the voltmeter and $\tau_{e}$ is the electrical time constant. Generally, the measurement is repeated with various shunt resistors, a procedure similar to that already pointed out.

In practice, a top non-pyroelectric layer (e.g., a black metal) with thickness $d_{1}$ is sometimes needed to cover the pyroelectric film with thickness $d_{2}$. For such a double layer structure supported on a heat sink, Holeman [78] found that temperature in the pyroelectric film varied in response to a sinusoidally modulated, laterally uniform heating as:

(35) $$T_{2}\left(x,\;t\right)=\left(A_{2}\cosh\left(\omega_{2}d_{2}\right)+B_{2}\sinh\left(\omega_{2}d_{2}\right)\right).\exp\left(j\omega t\right).$$

Similar to the procedure outlined for this case, it was found that the $I_{P}$ has a low frequency asymptote:

(36) $$\left|I_{low}\left(\omega \right)\right|=\frac{AP\left(F_{d}\right)d_{2}}{2G_{2th}}\omega .$$

In this regime, the top layer has no effect on the pyroelectric current. If $\tau_{1th}\ll \tau_{2th}$, then the $\left|I_{low}\left(\omega \right)\right|\propto \omega$ regime continues to dominate as long as $\omega \tau_{2th}<0.1$. However, if $\tau_{1th}\approx \tau_{2th}$ or $\tau_{1th}\gg \tau_{2th}$, then the $\left|I_{low}\left(\omega \right)\right|\propto \omega$ regime will only continue until either of the conditions $\omega \tau_{2th}<1$ or $\omega \tau_{1th}<1$ is violated. Once the regime $\left|I_{low}\left(\omega \right)\right|\propto \omega$ is left, the current quickly decays with frequency. A simple approach to measure $\tau_{1th}$ is given in [83]. The phase shift between the incoming radiation and the pyroelectric current reaches 180° at the frequency $\omega \tau_{1th}\approx 3\pi /2$, from which the thermal diffusion coefficient $\tau_{1th}$ is determined.

### 2.8. Interaction between the Processing and Electrical Properties of the PT-Based Titanate Thin Films

Processing conditions such as the deposition process, thin film composition, and thickness influence film morphology, domain microstructure, and defect concentration in a polycrystalline thin film and hence affect the electrical performance of the film. A broad range of grain sizes adversely affects the domain structure, degrading leakage current and reducing dielectric strength.

The study of conductivity of PZT ceramic with a Zr:Ti ratio of 53:47 in the temperature range of 150 °C<T<603 °C [32] revealed that processing history affects both electronic $\left(\sigma_{e}\right)$ and ionic $\left(\sigma_{I}\right)$ conductivities while the latter also shows dependence on the oxygen partial pressure. The leakage current in thin Pb (Zr, Ti)$O_{3}$ films is affected by the defect states and concentrations in the film, the electrode physical and chemical composition, and the voltage and temperature [26,84,85]. These effects are suspected to obscure differences in dielectric’s stoichiometry and together with differences in characterization protocols, they result in enormous discrepancies observed in the interpretation of results reported by different groups.

#### 2.8.1. The Influence of the Ca Content on Morphology and Electrical Characteristics of PCT Thin Films

Nagarbawadi [86] studied the influence of Ca doping on structural and electrical properties of bulk FE lead titanate ceramics prepared by mechanical mixing of their oxides in molar proportion and crystallized into perovskite phase.

1. Polycrystalline ceramic showed tetragonal structure with c/a ratio close to that of the pure PT, which decreased with increasing Ca mole percent.
2. Tetragonality disappeared at about 20 mole % of Ca.
3. The lattice parameters and volume of the unit cell decreases by increasing Ca content.
4. DC conductivity values at room temperature and around phase transition temperature (Tc), are thermally activated and are affected by Ca content.
5. Increase in Ca concentration lowers the curie temperature of the Ca-modified PT ceramic.
6. As the Ca concentration increases, grain size first increases up to 10 mol% but decreases consequently. This has been attributed to the solubility limit of Ca in PT.

**Figure 35:**
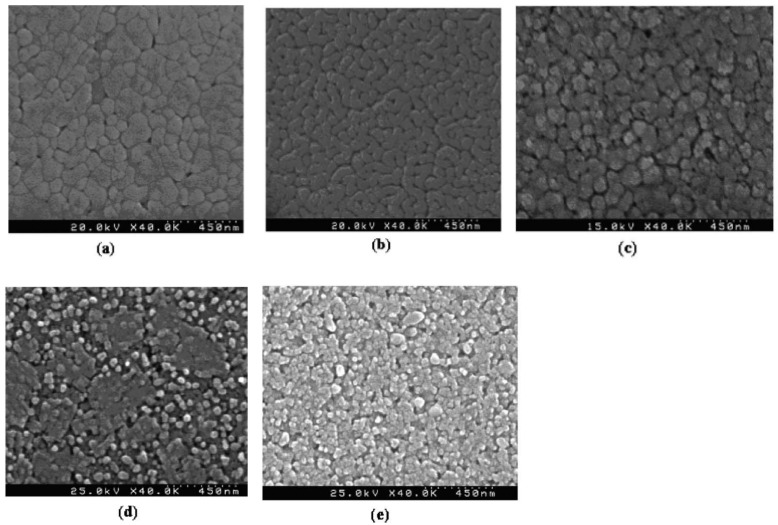
Surface morphology of PCT thin films with different Ca contents on Pt/Ti/SiO_2_/Si substrates after annealing at 650 °C for 15 min: (**a**) PCT (0), (**b**) PCT (25), (**c**) PCT (30), (**d**) PCT (40), and (**e**) PCT (50) [88]. Reprinted from [88], Figure 2, with the permission of AIP Publishing.

Huffman [87] reported on long switching and relaxation times for polycrystalline films of Ca modified PZT prepared by RF magnetron sputtering for Ca modified specimens. Chang and Lai [88] prepared capacitors utilizing magnetron-RF-sputtered ${Pb}_{1-x}{Ca}_{x}TiO_{3}$(PCT) thin films with different Ca contents (with x=0, 0.25, 0.3, 0.4, 0.5) as their dielectrics on $Pt/Ti/SiO_{2}/Si$ substrates to examine the influence of the Ca content on the morphology and electrical characteristics of PCT thin films. The SEM micrographs shown in Figure 35 reveal that grain uniformity in the film decreases as the Ca content is increased.

Their AFM data show that surface roughness of the films decreased with increasing in the Ca content. Also, an XRD analysis shows that as the Ca content is increased, the diffraction peak positions of the PCT thin films shift towards higher θ values. Furthermore, as x increases from 0 to 0.50 in PCT thin films (annealed at 650 °C for 15 min, measured at 0.3 kHz),

1. The relative permittivity of the films increases 148 to 356,
2. The dissipation factor increases from 2.5 to 2.84,
3. At T=50 °C, the pyroelectric coefficient increases from $0.8\times 10^{-4}\;C/m^{2}K$ to $1.89\times 10^{-4}\;C/m^{2}K$,
4. The coercive field decreases from 150 kV/cm for PCT 0 thin films to 40 kV/cm for PCT 50,
5. The remnant polarization decreases from $45.45\;C/m^{2}$ for PCT 0 to $6.81/m^{2}$ for PCT 50 thin films, and
6. PCT 30 thin film showed highest values for the figure of merit for voltage $F_{V}=3.3\times 10^{-2}\;m^{2}C^{-1}$ and the figure of merit for detectivity $F_{D}=8.62\times 10^{-7}{\;\left(m^{3}J^{-1}\right)}^{1/2}$.

The data suggest that the thin PCT 30 film is a good candidate for pyroelectric infrared (PIR) sensors. PIR sensors made of this material revealed that the voltage response and the specific detectivity increased with the x and this trend continued up to 3 mole %, Ca content, above which the voltage response and specific detectivity degraded.

#### 2.8.2. The Effect of Interfacial Diffusion across the Film Interfaces

Chi et al. [89] reported on the growth of a highly (100)-oriented ${Pb}_{0.8}{La}_{0.1}{Ca}_{0.1}{Ti}_{0.975}O_{3}\;(PLCT)/Pb\left({Nb}_{0.01}{La}_{0.1}{Zr}_{0.2}{Ti}_{0.8}\right)O_{3}\;(PNZT)$ multilayer film on a $Pt/Ti/SiO_{2}/Si$ substrate at a temperature as low as 450 °C. This technique takes advantage of the low temperature crystallization and high orientation capabilities of thin films of (PLCT), which minimizes the interfacial diffusion across boundaries of the multilayer film. These films are advantageous for applications in high figure-of-merit pyroelectric thin-film devices. This is because they simultaneously possess a relatively low dielectric constant and a high pyroelectric coefficient.

#### 2.8.3. The Effect of Contacts

Contacts to a PZT sensor may affect its electrical performance through:

1. The choice of contact composition.
2. The modification of the contact’s Schottky barrier height (if a blocking contact is formed).

##### The Choice of Contact Composition

Initially, platinum was used as both top and bottom contact for the FE−PZT devices because of the following advantages:

1. Pt is a non-reactive metal and hence suppresses formation of interfacial oxide with the oxygen containing PZT during high temperature steps of the fabrication processes.
2. The lattice spacing in the (111) Pt closely matches the (001) plane of PZT, and as a bottom electrode, it acts as a favorable growth template yielding highly textured [001] PZT, with the P directed normal to the electrode surfaces [90].

However, the growth of PZT on Pt produces high angle grain boundaries that cause aging and fatigue due to charge segregation and defect accumulation and amount to long-term device performance reliability issues [91]. Also, oxygen vacancies in PZT create several issues to pyroelectric detectors utilizing such sensing elements, which are initiated by the volatilization of PbO molecules since this induces both Pb and O vacancies. For PZT 43:57 compositions, Pb and O vacancies constitute the majority of defects and dominate the conduction mechanism up to 1000 °C [92,93]. Hence, the oxygen-permeable electrodes such as $IrO_{2}$ [94], $RuO_{2}$ [95], $SrRuO_{3},\left(La,Sr\right)CoO_{3}$ [26] were substituted with the top electrode material to reduce the concentration of oxygen vacancies in the PZT material by allowing the reintroduction of oxygen in to the PZT lattice during the post-annealing in the oxygen step. Unlike Pt electrodes, these materials allow for oxygen penetration into the oxide during the electrode annealing step, which reduces oxygen vacancies. Among these oxygen-permeable materials, iridium oxide has attracted much attention due to its thermal stability, reliable performance, relatively high conductivity, and high charge injection capabilities. Iridium oxide hardly reacts with Si, even at high temperatures [96], and makes a good barrier against $H_{2}$ [97] and Pb [96] diffusion. Yet, it is permeable to $O_{2}$ diffusion, a property that alleviates reliability issues in PZT device [94]. Iridium oxide is preferred over Pt as a top contact for pyroelectric applications. This is because:

1. Iridium oxide has good IR absorption properties, eliminating the need for a separate absorption layer such as electrochemically deposited Pt ‘black’ (with an absorption coefficient of ~90% [98]). This design would reduce the effective sensor’s thermal mass and hence provide faster response to the heat compared to Pt [57]. Iridium oxide serves a dual purpose as a top electrode/absorber that provides high FE polarization of the pyroelectric material, optimizes IR absorption, and minimizes the overall thermal mass.
2. Given the same top electrode area, iridium oxide provides a far higher pyroelectric coefficient for PZT films.
3. Iridium oxide has a higher dielectric constant and permittivity which translates to a higher pyroelectric responsivity compared to Pt [99,100].

Bouyssou et al. [94] proposed a qualitative and quantitative behavioral model which describes changes in the leakage current of the $IrO_{2}/PZT/Pt$ capacitor as a function of time, voltage, and temperature.

##### Ohmic versus Blocking Contacts

Blocking contacts present extra impedance to current flow through contact and result in an asymmetric response with respect to the direction of the current. Difficulties may arise for the extreme cases as outlined in Table 3.

Since pyroelectric currents are so small, non-ohmic contact may give a symmetric appearance to I(V) characteristics. This effect is of primary concern when p is being measured rather than when the performance of a practical device is being assessed.

##### The Choice of Electrode Layout

**Figure 36:**
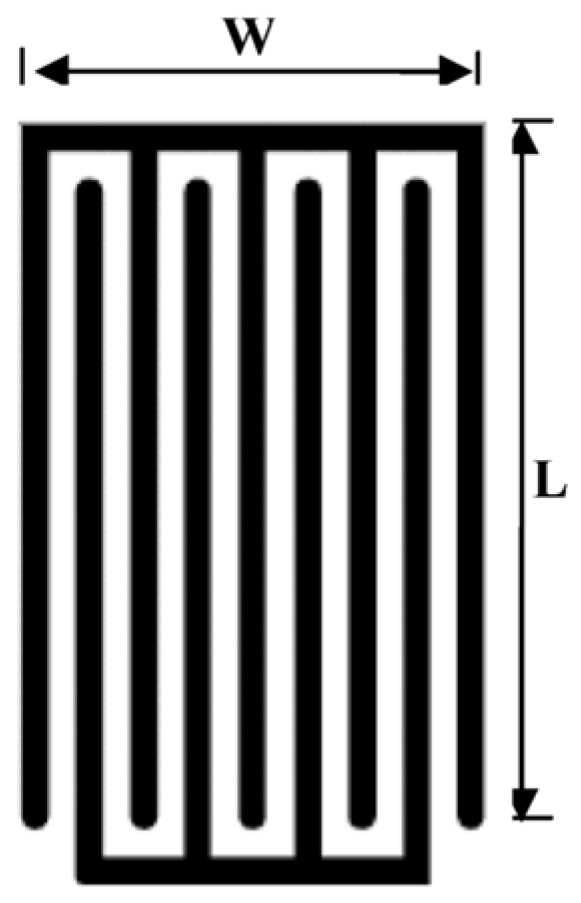
The layout for interdigitated fingers metallization adapted from [103]. Reprinted from [103], Figure 6, with permission from IEEE.

When a polarization vector has a component parallel to the surface, interdigitated fingers (Figure 36) are sometimes used on the top surface of the pyroelectric sensor to enhance current capability of the electrode. However, for the measurement of the pyroelectric constant of a material, such configurations are not the geometry of choice since much of the charge that is collected from electrodes incorporate dipoles due to the fringing field located well below the surface, thereby complicating the device analysis [62].

**Figure 37:**
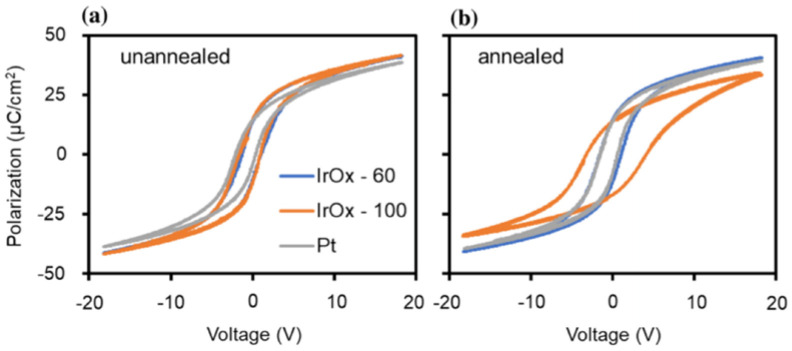
(**a**) Hysteresis loops of PZT capacitor devices before annealing and (**b**) after post-top electrode etch annealing in an $O_{2}$ environment at 650 °C for 30 min. $IrO_{x}$-60, $IrO_{x}$-100, and Pt electrodes have thicknesses of 100 nm, 700 nm, and 100 nm, respectively [100]. Reproduced from [100], Figure 9, with permission from Springer Nature.

Transient current characteristics depicted in Figure 37 follow the Curie-Von Schweidler law for 20 °C<T<100 °C, showing the relaxation behavior expected for FE-PZT thin films [94].

Hanrahan et al. [25] reported on the improvement of the leakage current, remnant polarization, and pyroelectric response of PZT thin film capacitors through the use of the $IrO_{x}$ top electrode. The hysteresis loops shown in Figure 37a for the unannealed $IrO_{x}$ capacitor seem nearly identical, with a minor increase in the maximum and remnant polarizations for the platelet $IrO_{x}$-100 specimen in Figure 35. Imprint is attributed to a passive layer within the film. Figure 37b reveals that the coercive voltage increases by 3X and maximum remnant polarization decreases in the plate-like structured $IrO_{x}$-100. This is ascribed to an additional low-permittivity dielectric layer arranged in series with the bulk of the PZT film [100].

**Figure 38:**
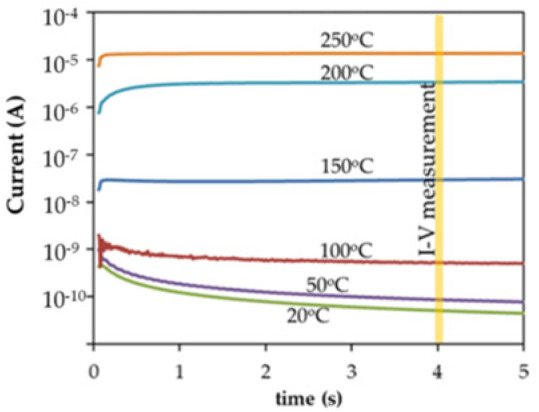
This Steady state current taken on a pre-poled sample subjected to 5 V bias, for temperature range 20 °C<T<250 °C, measured after ∼4 s [25]. Reproduced from [25], Figure 4, Figure 5 and Figure 7, with permission from IOP Publishing.

A transition takes place between 100 °C<T<150 °C, where the measured current increases and an initial current rise is observed at higher temperatures, indicating that a mechanism with relatively slow kinetics is in action. The leakage current in the thin film capacitors above 200 °C is dominated by the oxygen vacancy hopping. The sample had a Pt top electrode and the pyroelectric film consisted of a 15% excess Pb seed layer and a 5% excess PZT(52/48) thin film [25]. Figure 38 depicts the results when the bias voltage was switched between measurements. The data confirm the presence of a static Schottky interface at the bottom electrode. The difference in conductance is attributed to a measurement related artifact. Each high temperature I(V) test was performed on a fresh sample with supposedly uniform distribution of ionic species distributed through the thickness of the PZT. At the end of the positive voltage cycle, a large concentration gradient of ionic species was established within the PZT sample. The ionic species were electrically biased and thermally activated to drift through the thickness of the sample, during the initial testing cycle. As soon as the negative bias cycle begins, the asymmetrical distribution of ions screens the applied voltage and limits the conduction in this direction.

Leakage current in the thin film capacitors above 200 °C is dominated by the oxygen vacancy hopping.

Either Schottky emission, Poole-Frenkel emission, or a combination of these two dominates conduction [26,27].

**Figure 39:**
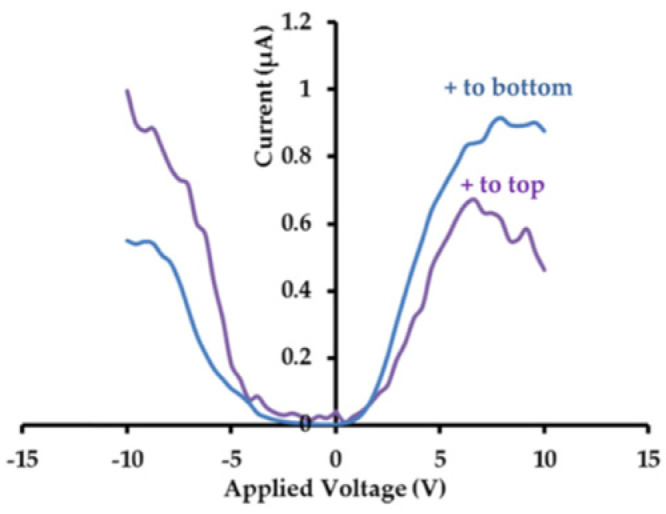
I(V) curves measured at 200 °C on a 20% excess Pb seed layer and 3% excess PZT(52/48) thin film, switching probed electrodes between tests. The results are shown for the 20s–3f Pt sample set [25]. Reproduced from [25], Figure 4, Figure 5 and Figure 7, with permission from IOP Publishing.

This study supports the assumption that at high temperatures, $V_{o}$ conduction primarily dominates leakage current with a secondary effect comprising the emission of electrons at one electrode, detected through the asymmetry in current density as shown in Figure 39.

## 3. Future Trends

The future of pyroelectric materials holds exciting prospects, with several emerging trends shaping the field. Researchers are increasingly exploring the development of multifunctional pyroelectric materials, integrating properties like ferroelectricity and piezoelectricity for versatile applications [104]. Nanotechnology is playing a pivotal role, with a focus on nanostructured pyroelectric materials that exhibit enhanced properties, paving the way for miniaturized devices and improved sensitivity [105]. The trajectory includes a move towards flexible and wearable pyroelectric devices, offering potential applications in health monitoring and energy harvesting from body heat [106]. Efforts are directed towards enhancing the efficiency of pyroelectric energy harvesting, with optimized materials and device designs [107]. The future may witness the evolution of smart and adaptive pyroelectric devices capable of dynamically responding to temperature changes for more versatile functionalities as pyroelectric sensors find utility in smart buildings, environmental monitoring, and energy management systems [108]. Advancements in characterization techniques and a focus on environmental and biomedical applications further highlight the diverse and promising future of pyroelectric materials [104].

## Figures and Tables

**Table 1:** Remnant polarization and coercive electric field for several processes.

Orientation, PCT Composition	Substrate	Fabrication Process	Remnant Polarization (μ$C/{cm}^{2}$)	Coercive Field (kV/cm)	Reference
PCT (24), (111)	Ti/Si$O_{2}$/Si (100)	Sol–gel, 10min RTA 600 °C in oxygen@100 °C/s.	18.2	210	[3]
PCT (30), c-axis	Pt-coated MgO	Multiple cathode sputtering	41	400	[5]
PCT (24)	Pt-coated silicon	Sol-gel	6	54	[6]

**Table 2:** Summary of the proposed conduction mechanisms in PT-based films.

Interface-Controlled	Bulk-Controlled	Mixed Model
Schottky emission [3,22,25,27]	Poole-Frenkel emission [26,27]	Mixed Schottky and Poole-Frenkel [28,29]
Two-carrier injection over Schottky-type [30]	Ionic conduction [22,31,32]	Internal-grain-boundary controlled conduction [33]
Carrier tunneling [34]	Space-charge limited conduction (SCLC) [3,35,36,37]	FE Schottky diode (for pure PT films) [38]

**Table 3:** Difficulties arising from the blocking contacts.

Method	Problem(s) That May Arise
1-Temperature ramping technique [101]	The detection of blocking contacts is challenging because obstruction of the current leaves no signature.
2-Chynoweth method [76]	Blocking contacts behave as a series capacitor and act as a ‘differentiating circuit’ leading to a sharp peak on I(t) and V(t) plots that is evident at the beginning of the heating or cooling cycle with a time constant $\tau \ll \tau_{\left(th\left(sys\right)\right)}$ *
	Initiation of non-symmetric pyroelectric response that are otherwise symmetric with respect to heating and cooling
3-Continuous oscillation technique [62]	Blocking contacts distort the frequency dependence of the pyroelectric currents, but diagnosis is difficult from the appearance of the pyroelectric data. The appropriate measurement technique is the impedance spectroscopy [102].
	Formation of very high resistance blocking contacts with a capacitance typically much larger than the capacitance of the pyroelectric sample itself is common for pyroelectric materials with highly mobile ions. The influence of the contacts is minimized by conducting measurements at a sufficiently high frequency.
	For thin films where the capacitance of the blocking layer is comparable with that of the sample, no general solution is known.

* $\tau_{\left(th\left(sys\right)\right)}$ is the system’s thermal time constant.

**Figure A1:**
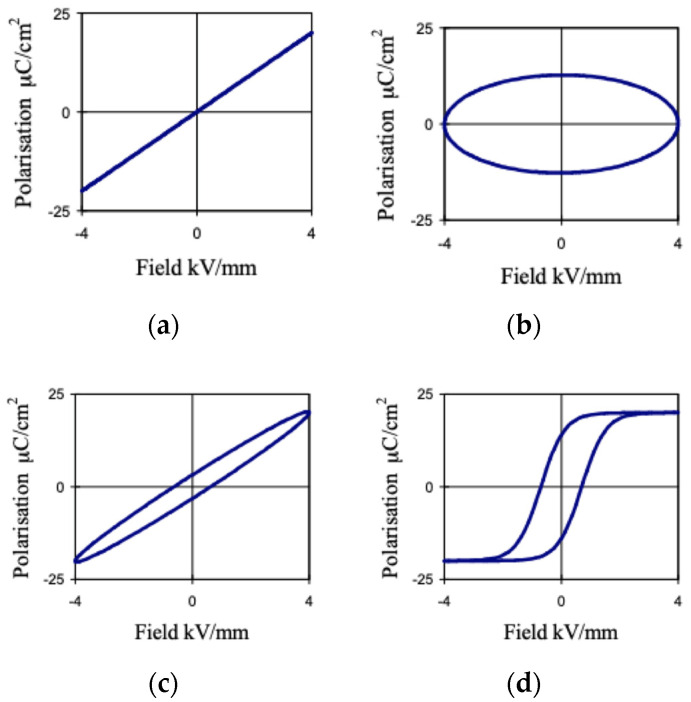
The P(E) loop for (**a**) an ideal linear capacitor, (**b**) an ideal resistor, (**c**) lossy capacitor, and (**d**) a non-linear lossy capacitor [109]. This figure is reprinted with permission from the journal.

**Figure A2:**
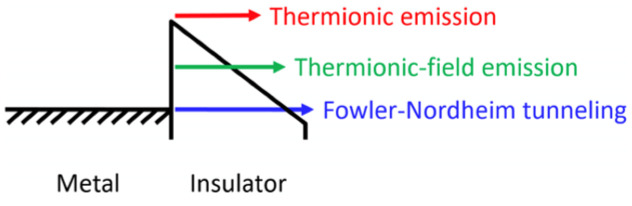
Classification of the three electrode limited leakage mechanisms in insulators: thermionic emission, thermionic-field emission, and Fowler–Nordheim tunneling. Reproduced from [114], Figure 1 (CC BY-NC 3.0).

**Figure A3:**
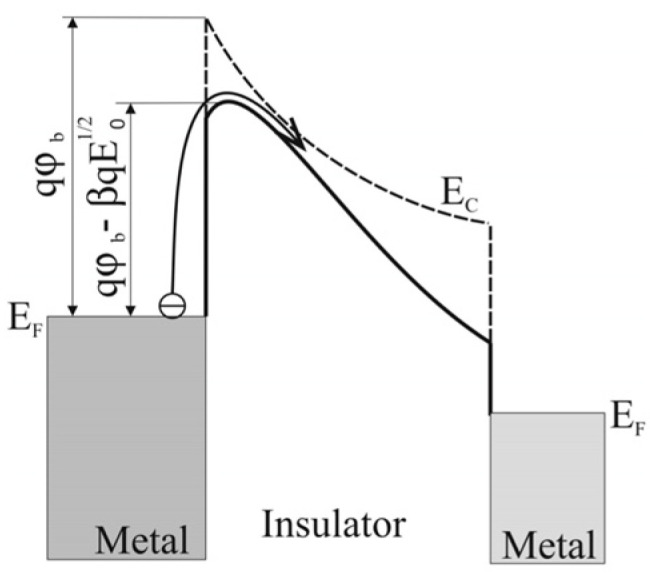
Band diagram of a metal–insulator–metal heterostructure at the Schottky emission conditions. $\beta ={\left(q/4\pi \varepsilon \right)}^{1/2}$β from [119]. Reproduced from [119], Figure 2, Figure 4 and Figure 7, with permission from MDPI.

**Figure A4:**
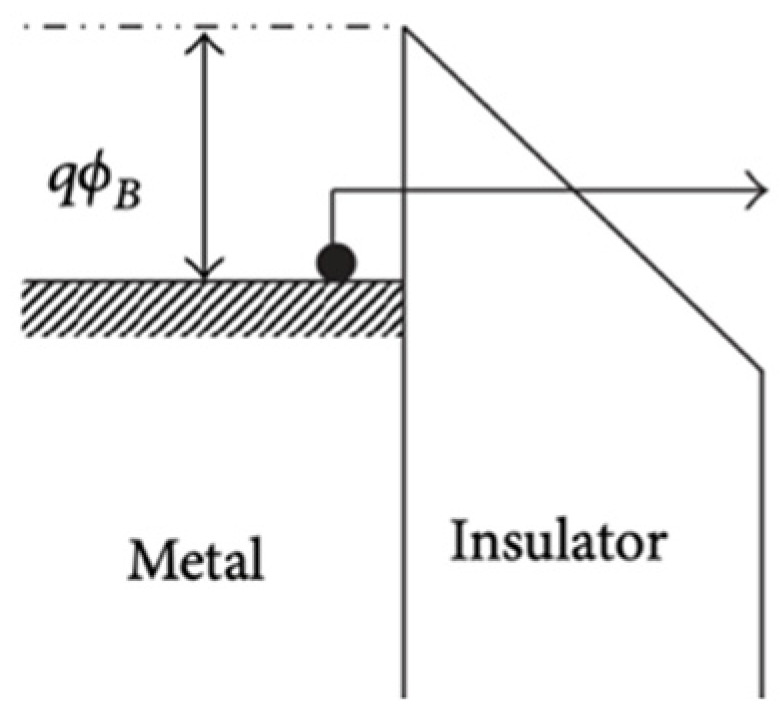
Schematic energy band diagram of thermionic field emission in an MIM structure [119]. Reproduced from [119], Figure 2, Figure 4 and Figure 7, with permission from MDPI.

**Figure A5:**
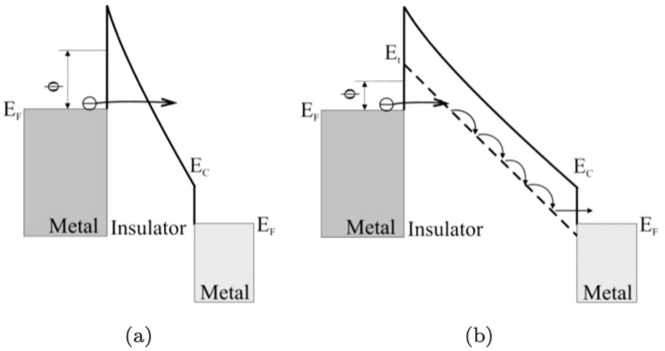
Band diagram of an MIM system under the condition of the F−N tunneling conduction mechanism for (**a**) thin insulator and (**b**) thick insulator with the trap levels $E_{t}$ below the conduction band from [119]. Reproduced from [119], Figure 2, Figure 4 and Figure 7, with permission from MDPI.

**Figure A6:**
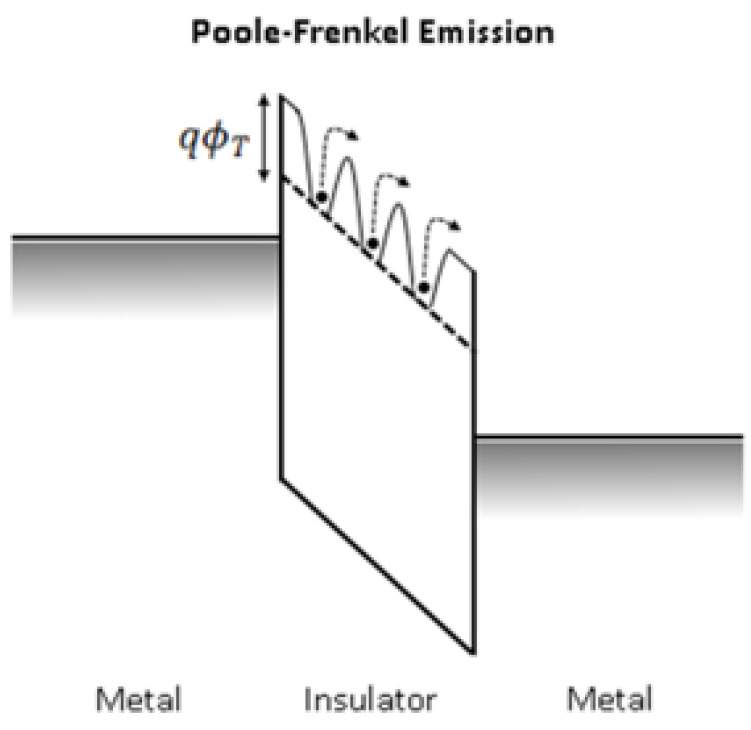
Schematic energy band diagram of Poole-Frenkel emission in MIM structure [120]. Reproduced from [120], with permission from MDPI.

**Figure A7:**
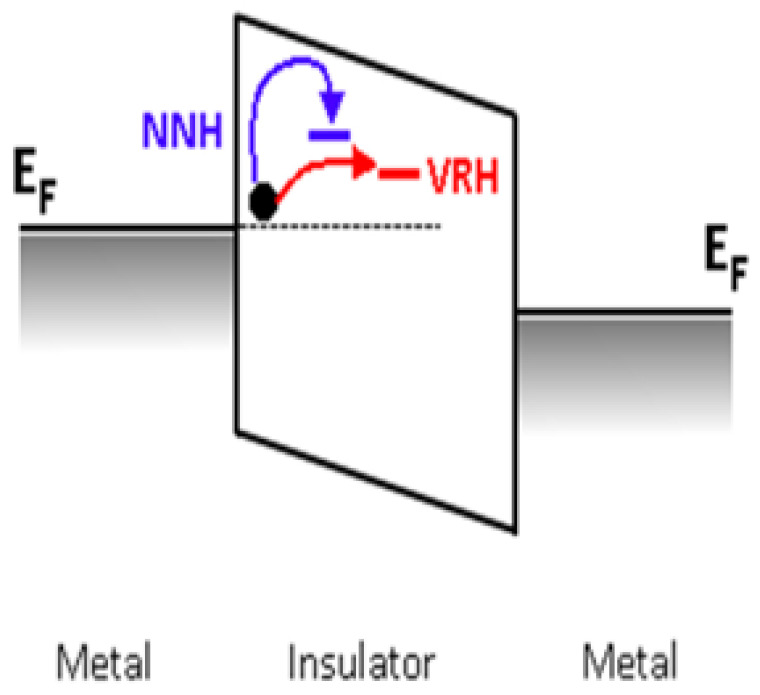
Schematic energy band diagram of Nearest Neighbor Hopping (NNH) and Variable-range Hopping (VRH) model in an MIM structure [120]. Reproduced from [120], with permission from MDPI.

**Figure A8:**
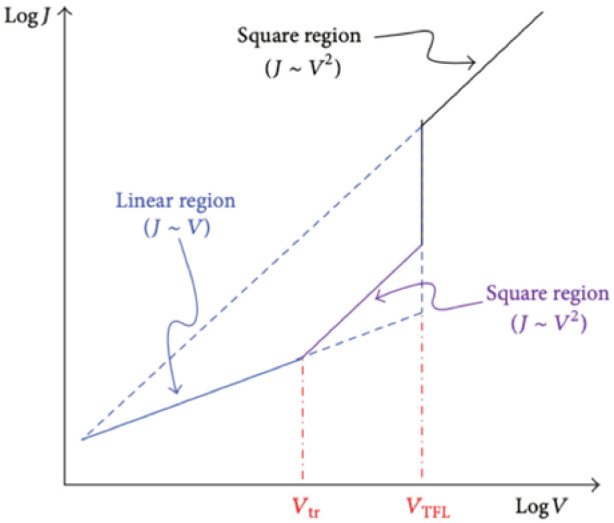
A typical current density-voltage characteristic of space-charge-limited conduction current. $V_{ohm}$ is called the transition voltage. $V_{TFL}$ is the trap-filled limit voltage [113]. “Copyright © 2014 Chiu, F.C. A review on conduction mechanisms in dielectric films. Advances in Materials Science and Engineering, 2014, 2014, pp. 1–18, Figure 24, Figure 28 and Figure 30. https://doi.org/10.1155/2014/578168. This is an open access article distributed under the Creative Commons Attribution License, which permits unrestricted use, distribution, and reproduction in any medium”.

**Figure A9:**
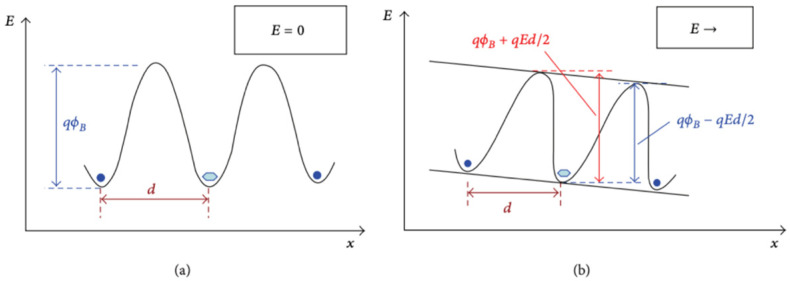
Energy band diagram of ionic conduction (**a**) without an applied electric field and (**b**) with the applied electric field [113]. Reproduced from Chiu et al. [113], an open access article distributed under the Creative Commons Attribution License, which permits “unrestricted use, distribution, and reproduction in any medium”.

**Figure A10:**
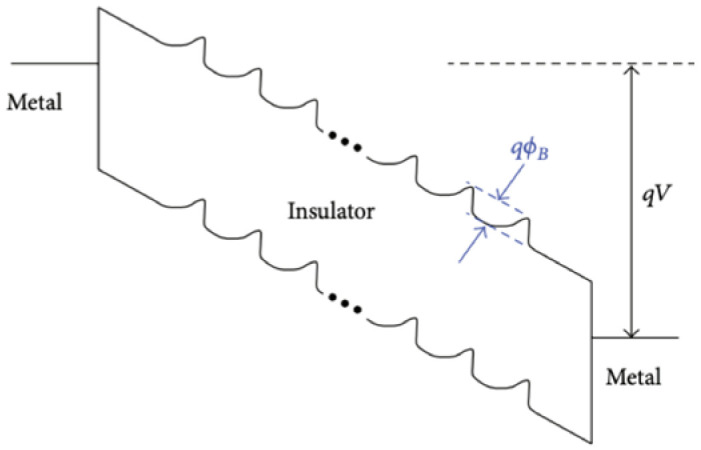
Schematic energy band diagram for grain-boundary limited conduction in a metal-insulator-metal structure [113]. Reproduced from Chiu et al. [113], an open access article distributed under the Creative Commons Attribution License, which permits “unrestricted use, distribution, and reproduction in any medium”.

**Figure A11:**
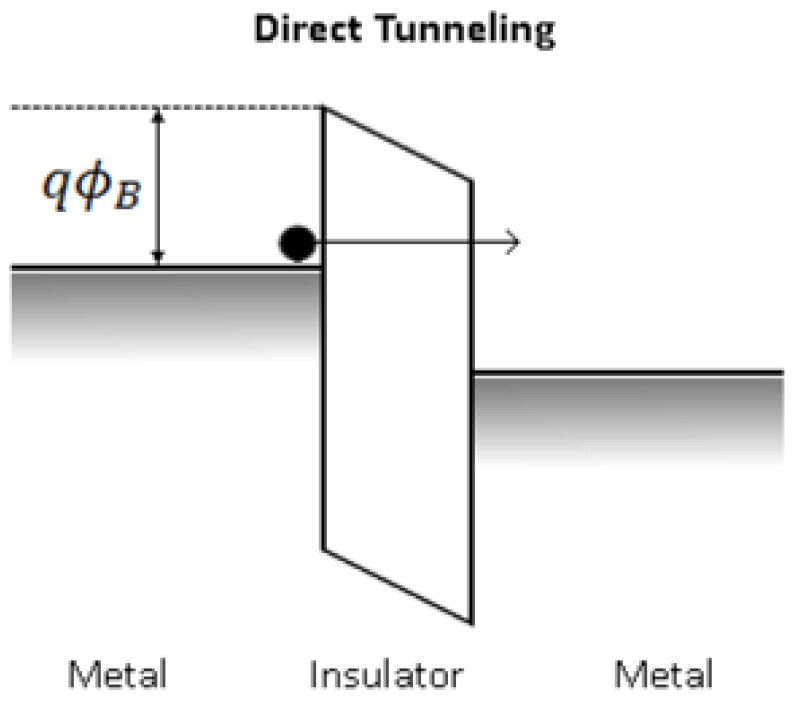
Direct tunneling of electrons [120]. Reproduced from [120], with permission from MDPI.

**Figure A12:**
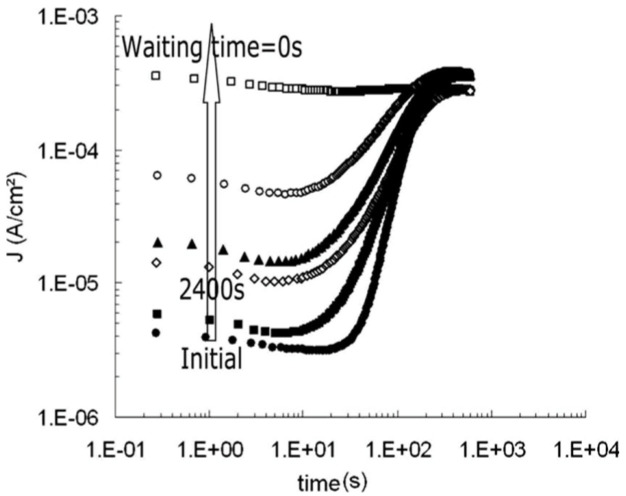
J(t) characteristics of PZT capacitors biased at 5 V at 140 °C during 600 s, after waiting periods ranging from 0 s to 2400 s [42]. Reversible RD mechanisms occur during the waiting period (i.e., the time during which 0 V is applied to the capacitor). Reprinted from [42], Figure 3, with permission from Elsevier.

**Figure A13:**
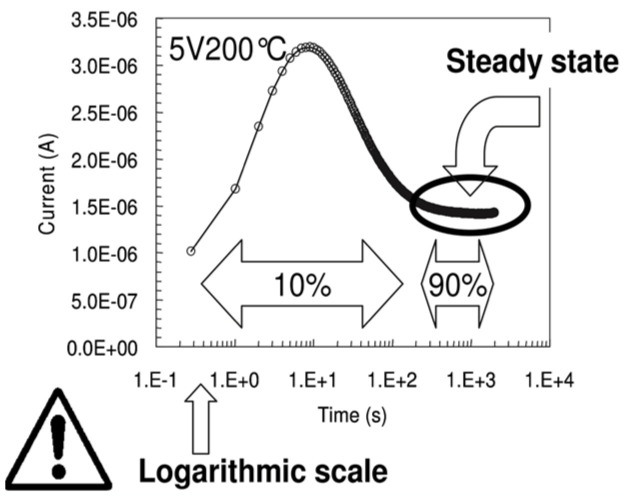
Typical evolution of leakage current for a capacitor submitted to a CVS of 5 V at a temperature of 200 °C. At the end of RR, the leakage current remains steady, and it lasts up to 90% of the capacitor lifetime [27]. Reprinted from [27], Figure 5, Figure 7, Figure 8 and Figure 14, with the permission of AIP Publishing.

**Figure A14:**
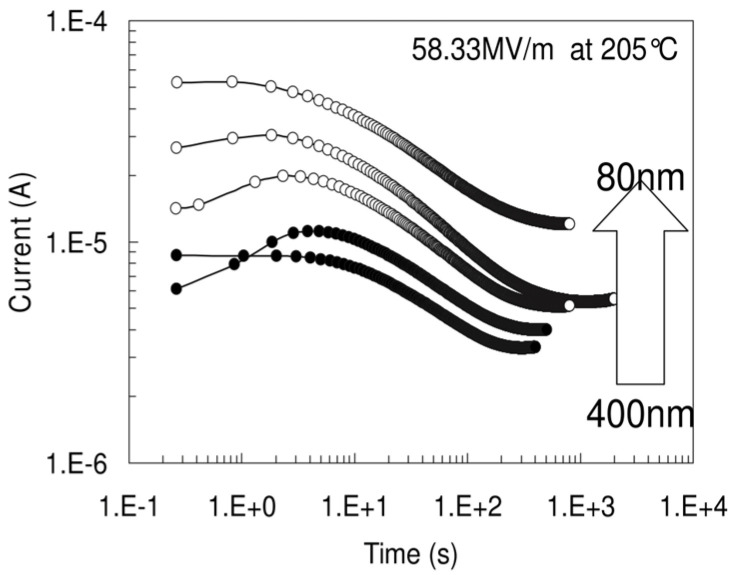
I(t) characteristics of prestressed capacitors before I(E) test. The prestress is performed under a constant electric field of 58.33 MV/m at 205 °C. The prestress is stopped when RR has ended [27]. Reprinted from [27], Figure 5, Figure 7, Figure 8 and Figure 14, with the permission of AIP Publishing.

**Figure A15:**
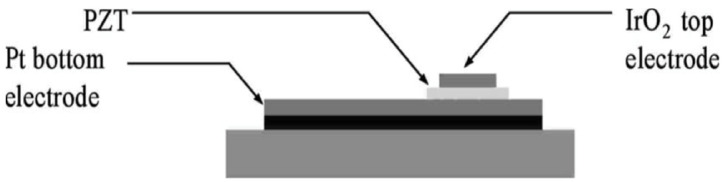
Schematic representation of tested structures [27]. Reprinted from [27], Figure 5, Figure 7, Figure 8 and Figure 14, with the permission of AIP Publishing.

**Figure A16:**
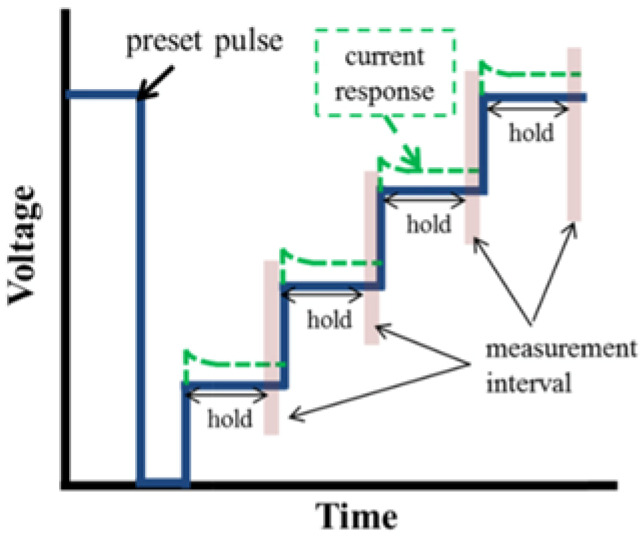
Schematic of a stepwise voltage increase utilized for I–V testing procedure. Samples are exposed to a pulsed voltage equal to the maximum bias to be experienced by the sample during the I(V) characterization. Charge is measured for 100 ms following a 4-s hold after a step voltage increase for all but one test, which used a 10-ms hold and measurement intervals. Voltage is swept from zero to the maximum positive voltage, either 5 or 10 V (10 or 20 MV/m equivalent electric fields), and then reset to zero and measured in the negative direction. For temperatures >200 °C, the bi-polar bias was reduced from 10 V to 5 V to prevent dielectric breakdown. Temperature was controlled with a resolution of ±5 °C [25]. Reproduced from [25], Figure 4, Figure 5 and Figure 7, with permission from IOP Publishing.

## Data Availability

No new data had been created as part of this study.

## Appendix A

### Appendix A.1. P(E) Loops: Development, Artifacts, and Measurement

For an ideal (linear) parallel-plate capacitor with FE insulator, the charge is linearly proportional to voltage (or field) as shown in Figure A1a. The P(E) loop is then a straight line passing through the origin, whose gradient is proportional to capacitance.

The charge $Q_{c}$ is given by:

(A1) $$Q_{C}=2EP_{r}A,$$

where $P_{r}$ is the remnant polarization and A is the electrode area. For an ideal resistor, where the current and voltage are in phase, the P(E) loop is generally an ellipse whose center rests at the origin (Figure A1b). If the electrical conductivity of the linear dielectric resistor is σ, the charge in transit is given by:

(A2) $$Q_{R}=\sigma Et,$$

where t is the measurement time. At fields below which FE switching occurs, ferroelectric material acts as a lossy linear dielectric and may be modeled by an equivalent resistor R representing the dielectric loss in parallel with an ideal capacitor C. Thus, the current supplied to this parallel RC network is:

(A3) $$I=\frac{V}{R}+\frac{CdV}{dt}.$$

Conceptually, the appearance of the P(E) loop for a linear lossy capacitor may be visualized by combining the above two components in parallel, as shown in Figure A1c. Here, the slope is proportional to the capacitance, but the area inside the loop is proportional to the loss tangent $\left(\tan\delta \right)$ of the device. If the electrical conductivity of the linear dielectric is σ, and the applied electric field is E, then:

(A4) $$Q_{R//C}=2EP_{r}A+\sigma Et.$$

For a non-linear dielectric such as that of a ferroelectric material where charges saturate at high electric field, a P(E) loop is produced, such as that shown in Figure A1d.

Several artifacts may arise during the measurement of P(E) loops. Some originate from measurement equipment, while others result due to the sample’s leakage current. A few examples are given in Table A1.

**Table A1:** Examples of sources for artifacts which may appear as hysteresis [110]. Reprinted from [110], with permission from the American Physical Society.

Appearance of the Hysteresis Curve	Description
	Dead short in a Sawyer-Tower bridge type measurement. This kind of result is obtained when the conductivity of the dielectric under test is high.
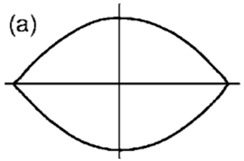
	Linear lossy dielectric. The points where the loop crosses $V_{a}=0$ are often misinterpreted as $P_{r}$ values. The elliptical shape is achieved because of the phase shift caused by the dielectric loss.
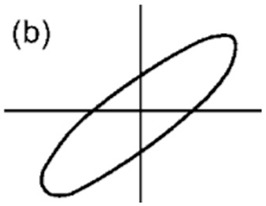
	Saturation of the amplifier in the measurement system leads to an apparently perfectly square hysteresis loop, particularly when the conductivity in (5) is large. When dipole reversal saturates, $P_{r}$ should not change with $V_{a}$ in a true hysteresis curve.
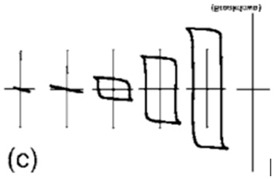
	P(E) resembles a real hysteresis curve for a nonlinear lossy dielectric even if it is phase-compensated. One can verify whether it is real or an artifact only by varying the measuring frequency. Artifacts due to dielectric loss are apt to be highly frequency dependent [111].
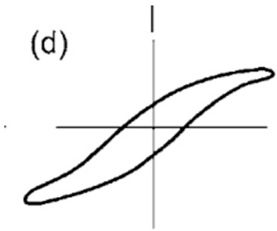

To construct P(E) loops, it is necessary to select a set of data representing a single cycle of the electric field waveform [112]. The data corresponding to the start and end points of the loop are identified by detecting when the electric field waveform crosses the horizontal time axis with either a positive or negative slope. The charge Q(t) stored in the device under test (DUT) at any given time is calculated via numerical integration of the current (I):

(A5) $$Q\left(t\right)=\int_{t_{2}}^{t_{1}}Idt.$$

Assuming that specimen area is A, the dielectric displacement (D) is calculated as:

(A6) $$D\left(t\right)=\frac{Q(t)}{A}.$$

For high permittivity ferroelectrics, P(t)≈D(t). However, for low permittivity dielectrics, a small correction is needed. Then, P(t) is calculated from [112]:

(A7) $$P\left(t\right)=\varepsilon_{0}E\left(t\right)-D\left(t\right).$$

### Appendix A.2. Measurement of the Dielectric Permittivity

It is given by $\varepsilon_{r}=1+P/E$, where P is the polarization density. Because E=0 under the static condition inside the conductor, for a metal, $\varepsilon_{r}\to \infty$. For a lossy dielectric, relative dielectric constant $\varepsilon_{r}^{*}$ is a complex number with two components: one in phase, $E\left(\varepsilon_{r}^{\prime }\right)$ and the other out of phase, $E\left(\varepsilon_{r}^{\prime \prime }\right)$. It is represented as $\varepsilon_{r}^{*}=\varepsilon_{r}^{\prime }-j\varepsilon_{r}^{\prime \prime }$. Upon the application of a voltage to such a dielectric, most of the absorbed energy is lost to heat. Dissipation factor (DF) or loss tangent $\left(\tan\delta \right)$ is defined as the inverse ratio of the dielectric’s capacitive reactance to its equivalent series resistance, or the ratio between the permittivity and the conductivity of an insulator $\left(\varepsilon_{r}^{\prime }/\varepsilon_{r}^{\prime \prime }\right)$ at a specified frequency. DF is a dimensionless quantity that indicates how much energy a material loses to heat. For an ideal insulator, DF=0. The ratio $\varepsilon_{r}^{\prime }/\left|\varepsilon \right|$ is called power factor.

Debye’s relaxation model characterizes the dynamic polarization response of an ideal, noninteracting population of dipoles with only one relaxation time to an alternating external electric field. Assuming $\varepsilon_{\infty }$ and $\varepsilon_{S}$ are the low (static) and high frequency limits of the permittivity, respectively, it is expressed in the complex permittivity (ε) of a medium as a function of the angular frequency of the electric field (ω) as:

(A8) $$\varepsilon^{*}\left(\omega \right)=\varepsilon_{\infty }+\frac{∆\varepsilon }{\left(1+i\omega \tau \right)}\;,$$

where $∆\varepsilon =\varepsilon_{S}-\varepsilon_{\infty }$ and τ is the characteristic relaxation time of the medium. The real and imaginary parts of the complex dielectric permittivity $\varepsilon^{*}\left(\omega \right)$ are given by (A9) and (A10) respectively:

(A9) $$\varepsilon^{\prime }=\varepsilon_{\infty }+\frac{\varepsilon_{S}-\varepsilon_{\infty }}{\left(1+\omega^{2}\tau^{2}\right)},$$

(A10) $$\varepsilon^{\prime \prime }=\frac{\varepsilon_{S}-\varepsilon_{\infty }}{\left(1+\omega^{2}\tau^{2}\right)}.$$

The dielectric loss (loss tangent) is given by:

(A11) $$\tan\delta =\frac{\varepsilon^{\prime \prime }}{\varepsilon^{\prime }}=\frac{\left(\varepsilon_{S}-\varepsilon_{\infty }\right)\omega \tau }{\left(1+\varepsilon_{\infty }\omega^{2}\tau^{2}\right)}.$$

To measure $\varepsilon_{r}$, a small AC signal superimposed upon a DC bias is applied to the sample, and an impedance analyzer is used to measure the real and imaginary parts of the impedance. By doing so, the impedance analyzer balances sample impedance with a set of internal reference impedances. There are now commercial systems that automatically measure both the capacitance and loss tangent.

### Appendix A.3. Transport Mechanisms in Thin Films

A knowledge of conduction mechanisms in the dielectric films is indispensable since it helps to identify device failure mechanisms and allows for the minimization of the undesirable leakage current. Transport characteristics in thin dielectric films may be traced either to the contact (contact-limited) or to the bulk (bulk-limited) properties of the dielectric. In practice, depending on the specific experimental conditions (applied field, temperature, illumination density, etc.), one or more of the mechanisms discussed below may govern the overall I(V) characteristics. The methods for distinguishing between these mechanisms rely on how conductivity is influenced by temperature and/or the applied voltage [113].

#### Appendix A.3.1. Contact-Limited Conduction Mechanisms

If a blocking contact exists at a metal-dielectric interface, the conduction process is said to be electrode-limited and ordinarily, it will not become bulk-limited with increasing applied voltage unless the dielectric is thick.

Figure A2 schematically shows the three mechanisms an electron may use to pass through a barrier. These are: (i) surmounting the barrier by thermionic (Schottky) emission, (ii) passing through the barrier (Fowler-Nordheim tunneling), and (iii) a thermal excitation followed by a tunneling through a narrow triangular barrier (thermionic-field emission).

##### Thermionic (Schottky) Emission

Thermionic emission is the prevalent transport mechanism in perovskites and metal insulator (M/I) interfaces, especially at relatively high temperatures. It describes a conduction process initiated by thermally activated electrons that are injected over an energy barrier into the conduction band of the perovskite/oxide. Then, by providing sufficient energy, an externally applied field can enable more electrons (compared to equilibrium) to confront the barrier and encourage them to overcome the barrier The conduction is thus limited by the emission of electrons over the M/I barrier. The current density $\left(J_{SE}\right)$ is given by:

(A12) $$J_{SE}=A^{*}T^{2}\exp\left[\frac{-q\left(\varphi_{B}-\sqrt{\frac{qE_{0}}{4\pi \varepsilon }}\right)}{kT}\right],$$

where $A^{*}=2q\mu E_{0}{\left(\frac{2\pi mkT}{h^{2}}\right)}^{3/2}$ is the effective Richardson constant, q is the elementary charge, $m^{*}$ is the electron effective mass in the dielectric insulator, k is Boltzmann’s constant, q is the electronic charge, T is the absolute temperature, h is Planck’s constant, $E_{0}$ is electric field at the M/I interface (or across the oxide), µ is carrier mobility, $q\varphi_{B}$ is the barrier height (i.e., conduction band offset), and $\varepsilon =\varepsilon_{0}\varepsilon_{r}$, where $\varepsilon_{0}$ is the vacuum permittivity and $\varepsilon_{r}$ is the optical dielectric constant. The optical dielectric constant, rather than the static dielectric constant, is used because the dielectric constant is a function of frequency. During the emission process, if the electron jump period from the metal’s Fermi level at the M/I interface to the barrier top is shorter than the dielectric relaxation time, the dielectric will not have enough time to be polarized. Consequently, the proper choice for ε is the optical (high frequency) dielectric constant [115,116], which is smaller than the static (low frequency) dielectric constant, where more polarization mechanisms contribute to the total polarization [117]. The dynamic dielectric constant $\varepsilon_{r}=n^{2}$, where n is the insulator’s refractive index and is thus generally a function of frequency.

The I(V) characteristic is asymmetric with respect to positive and negative voltages. Although the Schottky equation is widely used to describe the conduction in perovskite-type titanate films, it generally yields inconsistent values for $A^{*}$ and ε. Zafar et al. [41] resolved this issue by modifying the standard Schottky equation. Furthermore, they showed that at lower frequencies, charge injection in $SrTiO_{3}$, a substance which has proved to be effective as contact for most perovskites’ films, can be adequately described by the Schottky expression, while at higher fields, unified thermionic and field emission of the approach proposed by Murphy and Good [118] is more consistent.

When an electron departs metal, it leaves behind a positive ion. This process leads to the effective reduction of the Schottky barrier height (called image-force-induced Schottky barrier lowering) by an amount given by [23]:

(A13) $$∆\Phi =\sqrt{\frac{q^{3}E_{0}}{4\pi \varepsilon }},$$

Since the tunneling exponentially depends on the barrier height, image-force barrier lowering effectively increases the insulator’s tunneling current. From Equation (A13), it follows that:

(A14) $$\mathrm{Ln}\left(J_{SE}/A^{*}T^{2}\right)=-\frac{q\varphi_{B}}{kT}+\left(\sqrt{\frac{qE_{0}}{4\pi \varepsilon }}/kT\right)$$

Thus, if the Schottky injection is the dominant mechanism for the carrier transport, a plot of $\mathrm{Ln}\left(J_{SE}/E_{0}T^{\frac{3}{2}}\right)$ versus ${\left(E_{0}\right)}^{1/2}$ is linear with a slope of qβ/kT.

##### Thermionic-Field Emission

Figure A4 depicts a schematic of the energy band diagram for the thermionic-field emission, where electrons having an energy between the metal’s Fermi energy (capable of field emission) and the dielectric’s conduction band edge (capable of Schottky emission) tunnel through the triangular barrier. The current density due to thermionic-field emission can be roughly expressed as [7]:

(A15) $$J_{TFE}=\frac{q^{2}\sqrt{mkT}E}{2h^{2}\sqrt{\pi }}\exp\left(-\frac{q\Phi_{B}}{kT}\right)\exp\left[\frac{h^{2}q^{2}E^{2}}{96m{\left(kT\right)}^{3}}\right],$$

##### Fowler-Nordheim (F-N) Tunneling

Figure A5a depicts the schematic energy band diagram of F-N tunneling. When the insulator thickness is less than 100 A°, electron wave function may penetrate through the potential barrier. The electron tunneling probability depends not only on the thickness, but also on the height, width, and the structure of the barrier, as well as the applied electric field.

The expression of the F-N tunneling current is:

(A16) $$J_{FN}=\frac{q^{3}E_{0}^{2}}{8\pi hq\Phi }\exp\left[\frac{-8\pi {\left(2qm_{T}^{*}\right)}^{1/2}}{3hE_{0}}\right]\Phi^{3/2},$$

where $m_{T}^{*}$ is the electron’s tunneling effective mass in the dielectric. The other notations in the equation above have already been described earlier. Experimental results conform with theory, particularly at higher electric fields. A plot of $\ln\left(J/E^{2}\right)$ versus 1/E (F−N plot) is linear, with a slope $\left(S_{PF}\right)$, which is a function of both electron effective mass and barrier height:

(A17) $$S_{PF}=\left[\frac{-8\pi {\left(2m_{T}^{*}\right)}^{1/2}}{3qh}\right]\Phi^{3/2},$$

Despite the fact that tunneling through thick insulators (>50 A°) is highly unlikely, Stolichnov et al. [44] reports on detection of entirely direct tunneling currents in a 450 nm thick PZT films at temperatures between 100 K and 140 K. Their observation could be explained if it is assumed that shallow traps existed in the insulator band gap. In this case, an electron may tunnel into a trap level and is then transported through the insulator via hopping conduction, as shown in Figure A5b. Then, the barrier height Φ obtained using Equation (A17) will give the energy position of traps $E_{t}$ in the dielectric band gap with respect to the metal’s Fermi level. Nevertheless, the range of data on the electric field (from 2.2 MV/cm to 2.8 MV/cm) is too limited to unambiguously identify the current mechanism.

Since the tunneling probabilities depend on the shape of the tunneling barrier, different probabilities (and hence currents) are obtained for the FN, where the barrier is triangular, and for the direct tunneling, where the barrier is trapezoidal.

In general, if insulator thickness is wide enough, the tunneling effective mass can be assumed to be equal to the electron effective mass for dielectric films. To extract $m_{T}^{*}$ and $\phi_{B}$, it is useful to measure the I(V) at a high temperature where thermionic emission dominates and at a low temperature where the tunneling current dominates [113]. Using a mathematical iteration method, the electron effective mass in the insulator and barrier height at the interface can then be determined using the intercept of Schottky plot at high temperatures and the slope of F−N plot at low temperatures.

#### Appendix A.3.2. Bulk-Limited Conduction Mechanisms

These mechanisms solely depend on the electrical properties of the bulk dielectric insulator such as the trap distribution and energy level and comprise (1) Poole-Frenkel emission, (2) hopping conduction, (3) ohmic conduction, (4) space charge-limited conduction, (5) ionic conduction, and (6) grain-boundary-limited conduction. The study of the bulk-limited conduction mechanisms can reveal some of the important electrical properties in the dielectric films, including the trap energy level, spacing, and density as well as the carrier drift mobility, the dielectric relaxation time, and the density of states in the dielectric conduction band.

##### Poole-Frenkel (P-F)

Poole-Frenkel emission involves the thermal excitation of electrons from bulk traps (e.g., oxygen vacancies) into the dielectric conduction band. Assuming that Coulomb interaction exists between electrons and traps, the potential energy of a trapped electron is then reduced when an electric field is applied across the dielectric and the probability that a trapped electron is thermally emitted into the conduction band of the insulator is increased, as schematically depicted in Figure A6.

In contrast to Schottky emission, for P-F conduction mechanism, the I-V characteristic is symmetric with respect to positive and negative voltages [45].

The current density due to the P-F emission is given by:

(A18) $$J_{PE}=q\mu N_{C}E_{0}\exp\left[\frac{-q\left(\phi_{T}-\sqrt{\frac{qE_{0}}{\pi \varepsilon }}\right)}{kT}\right],$$

where μ is the electron drift mobility, *NC* is the density of states in the dielectric conduction band, and $q\emptyset_{T}$ is the trap energy level. The other relevant notations were defined earlier. The P-F mechanism relies on thermal activation and externally applied electric field and thus dominates under such circumstances. The trap energy level can be extracted experimentally from the Arrhenius plot of $\ln\left(J_{PF}/E_{0}\right)$ versus $E_{0}^{2}$ (P-F plot). The trap barrier height is extracted from the intercept of P-F plot.

The distinction between the Schottky and PF mechanisms is sometimes challenging. This is because, from Equations (A12) and (A16), it follows that:

(A19) $$\ln\left(J_{Sch}\right)={\ln\left(J_{Sch}\right)|}_{E_{0}=0}+S_{Sch}\sqrt{E_{0}},$$

(A20) $$\ln\left(J_{PF}\right)={\ln\left(J_{PF}\right)|}_{E_{0}=0}+S_{PF}\sqrt{E_{0}},$$

where $S_{PF}$ and $S_{Sch}$ represent the slopes of $\ln\left(J_{PF}\right)$ and $\ln\left(J_{Sch}\right)$ versus $\sqrt{E_{0}}$, respectively. Considering Equations (A19) and (A20), both the Schottky and PF models may fit fairly well with experimental data irrespective of the dielectric thickness. To distinguish between the two mechanisms, Chantier et al. [27] proposed extracting the refractive index (n) as follows:

(A21) $$n=\sqrt{\varepsilon }=\frac{q}{kTS_{X}},$$

where x represents either the Schottky or PF mechanism. Then, the comparison between the theoretical values reported in the literature with the experimental data reveals the dominant current mechanism. As a final means to distinguish between the Schottky emission and P-F mechanism, the effect of electrode material on the conduction characteristics can be examined.

##### Hopping Conduction

A trapped electron may initiate a conduction by tunneling into its nearest neighbor trap site. This process is referred to as nearest neighbor hopping (NNH). The current density is given by:

(A22) $$J_{NNH}=\sigma_{0}E\exp\left(-T_{0}/T\right),$$

where $\sigma_{0}$ is the electrical conductivity at ${T=T}_{0}$. A second hopping conduction mechanism known as Mott variable range hopping (VRH) is observed in strongly disordered systems with localized charge-carrier states, where the electron is likely to hop into a trap that is further away but has lower trap energy. The energy band diagram of Figure A7 describes both the NNH and VRH processes [120].

In the P-F emission, carriers need to overcome the trap barrier through a thermionic mechanism, while the hopping conduction relies on a tunneling effect. In hopping conduction, the carrier energy is lower than the maximum energy of the potential barrier between the two trapping sites, but the carriers can still transit using the tunneling mechanism.

Simulation of hopping conduction allows for determining the range of electric field and temperatures for which hopping conduction becomes dominant. Using the slopes of the Arrhenius plot in low fields, the activation energy is extracted. Defects with deeper energy levels are activated at higher temperatures. This leads to an exponential decrease in J. For Mott VRH transport, the current density is given by [120]:

(A23) $$J_{NNH}=\sigma_{0}E\exp{\left(-T_{0}/T\right)}^{1/4},$$

where $\sigma_{0}$ is the electrical conductivity at $T_{0}$. Similar to Ohmic conduction, J is proportional to the applied electric field. Nevertheless, the conductance σ obeys temperature dependency given by $\ln\left(\sigma \right)\propto T^{-1/4}$.

##### Trap-Assisted Tunneling (TAT)

This is another possible hopping mechanism attributed to the tunneling current which is assisted by the defects in the dielectric. Unlike F-N or direct single-step tunneling, the traps in dielectric assist the electrons to tunnel from cathode to anode through a 2-step process by first capturing electrons from the cathode and subsequently emitting it to the anode. For inelastic TAT, the electron will relax to the trap’s energy level by emitting one or more phonons. The expression for the TAT current density is [120]:

(A24) $$J_{TAT}=A\exp\left(\frac{-8\pi \sqrt{2qm^{*}}}{3hE}\Phi_{T}^{3/2}\right),$$

where A is a constant and $\Phi_{T}$ is the energy of the electron traps with respect to the conduction band edge of the dielectric.

##### Ohmic Conduction

Figure A8 shows a schematic energy band diagram for the Ohmic conduction. Even for dielectrics with large band gaps, electrons from valance band or from the impurity levels within the energy gap may still become thermally excited.

Ohmic conduction stems from the movement of thermally generated carriers under an applied field and leads to a linear relationship for J(E), expressed as:

(A25) $$J_{ohm}=\sigma E=nq\mu E\;,$$

where σ is electrical conductivity, μ is electron mobility, and E is the electric field across the dielectric. The concentration of electrons n in the conduction band is given by:

(A26) $$n=N_{C}\exp\left(E_{F}-E_{C}\right)/kT.$$

Here, $N_{C}$ is the effective density of states in the conduction band; the other terms are as defined above. Because the carrier concentration is negligible, $E_{F}\sim E_{i}$ and $\left(E_{C}-E_{F}\right)\sim E_{g}/2$. Hence,

(A27) $$J_{ohm}=q\mu EN_{C}\exp\left(\frac{-E_{g}}{2kT}\right),$$

This current component is very small in magnitude and is observed at very low voltages in I(V) characteristics of the dielectric films [113].

##### Space-Charge-Limited Current (SCLC)

When charges are injected through ohmic contacts in an insulator with traps distributed throughout its band gap, a large fraction of the charges may become trapped in the insulator. These trapped charges reduce the magnitude of the SCLCs and modify the shape of the J(V) from an ideal square law to a higher power dependence on voltage. Furthermore, since the occupancy of a trap is a strong function of temperature, SCLC will have a temperature dependence that depends on the trap energy. In practice, the SCLCs are less than their theoretical value for an ideal crystal by the ratio of free to trapped carriers. Therefore, the SCLCs may be utilized as a simple tool for measuring the imperfections in crystals, even in the range of one part in $10^{15}$.

As depicted in Figure A8, a typical J(V) characteristic for space-charge-limited current plotted in a log-log format is bound by the three limiting curves: (i) ohm’s law $\left(J_{Child}\propto V\right)$, (ii) traps-filled limit $\left(J_{TFL}\propto V^{2}\right)$, and (iii) the Child’s law $\left(J_{Child}\propto V^{2}\right)$. $J_{ohm}$ and $J_{TFL}$ mark the transition voltages at the boundary between ohm’s law and TFL curve, respectively [121]:

(A28) $$J_{ohm}=qn_{th}\mu V_{a}/d,$$

(A29) $$J_{TFL}=\left(9/8\right)\mu \varepsilon \theta V_{a}^{2}/d^{3},$$

(A30) $$J_{Child}=\left(9/8\right)\mu \varepsilon V_{a}^{2}/d^{3},$$

(A31) $$V_{ohm}=\left(9/8\right)\times qn_{th}d^{2}/\left(\varepsilon \theta \right),$$

(A32) $$\theta =\left(N_{C}/g_{n}\right)N_{t}N\exp\left(E_{t}-E_{C}/kT\right),$$

(A33) $$V_{TFL}=qN_{t}d^{2}/2\varepsilon ,$$

(A34) $$\tau_{tr}=d^{2}/\mu \theta V_{ohm},$$

(A35) $$\tau_{el}=\varepsilon /qn\mu \theta ,$$

where $n_{th}$ is the concentration of the free charge carriers in thermal equilibrium, $V_{a}$ is the applied voltage, d is the thickness of thin films, ε is the static dielectric constant dielectric constant, and θ is the quantity defined in Equation (A32). The transit and the dielectric relaxation time are denoted by $\tau_{tr}$ and $\tau_{el}$, respectively.

Table A2 gives further details for carriers’ distribution in dielectric film under carriers’ weak injection $\left(V\leq V_{ohm}\right)$ under space-charge-limited conduction.

**Table A2:** Carriers’ distribution in dielectric film under carriers’ weak injection $\left(V\leq V_{ohm}\right)$ [113]. Reproduced from Chiu et al. [113], an open access article distributed under the Creative Commons Attribution License, which permits “unrestricted use, distribution, and reproduction in any medium”.

Regime	Conditions	Characteristics	Schematic of the Physical Process
Very weak injection $V_{a}\ll V_{ohm}$	$V_{drift}$: slow $\tau_{tr}>\tau_{rel}$ $n_{inj}\ll n_{th}$ $E_{t}\ll E_{fn}$	Space charge exists next to the contact.	
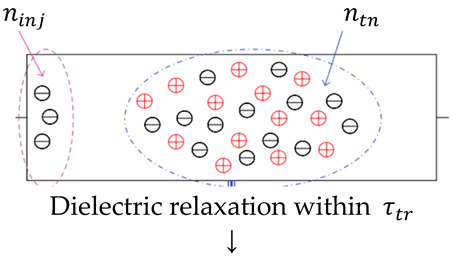
Dielectric relaxation $V_{a}<V_{ohm}$	$\tau_{tr}>\tau_{rel}$, thus $n_{inj}>n_{th}$. For charge neutrality and injected carriers. Redistributed relaxed in a time $\sim \tau_{rel},\;E_{t}<E_{fn}$	The injected carriers cannot travel across the insulator.	
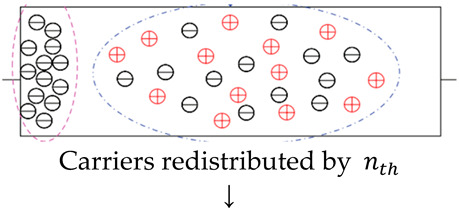
Weak injection $V_{a}=V_{ohm}$	$n_{inj}=n_{th}$, $\tau_{tr}=\tau_{rel}$, $E_{t}=E_{fn}$ Traps are partially filled.	$V_{a}=V_{ohm}$, marks border between the ohmic and SCLC (1st square law) regimes.	
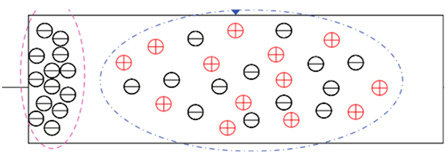

Table A3 gives details for carrier distribution in dielectric films under strong injection $\left(V_{ohm}\leq V_{a}<V_{TFL}\right)$ and in space-charge-limited conduction $\left(V_{TFL}\leq V_{a}\right)$.

**Table A3:** Carriers’ distribution in dielectric film: the case of strong injection, where the traps are filled up and a space charge appears [113]. Reproduced from Chiu et al. [113], an open access article distributed under the Creative Commons Attribution License, which permits “unrestricted use, distribution, and reproduction in any medium”.

Regime	Conditions	Conclusion	Schematic of the Physical Process
Onset of square law: $V_{a}\sim V_{ohm}$, $\tau_{tr}$, is too short for charges due to $n_{inj}$ to be relaxed by those due to $n_{th}$	$\tau_{tr}\leq \tau_{rel}$, $n_{inj}>n_{th}$, both $\tau_{tr}$ and $\tau_{rel}\;$decrease as $V_{a}$ is increased. $E_{t}\leq E_{fn}$, traps remain empty, and dielectric is not relaxed.	Injected carriers transit the device without noticing the traps. $J\propto V^{2}$ applies.	
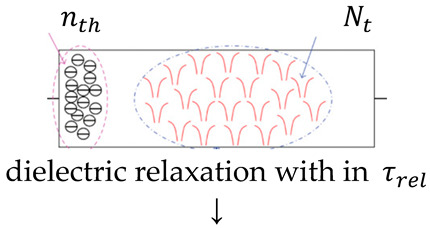
Trap-limited: $V_{ohm}<V_{a}<V_{TFL}$, while $V_{a}$ reaches $V_{TFL}$, the traps are progressively filled.	For $n_{inj}<n_{th}<N_{t}$, as $V_{a}$ increases, $n_{inj}$ also increases. Thus $E_{Fn}$ moves up in energy but still $E_{Fn}<E_{t}$. Some of the traps are filled with injected carriers.	Trapped-filled limited trapped behavior: dielectric becomes partially relaxed in a time $\sim \tau_{rel}$.	
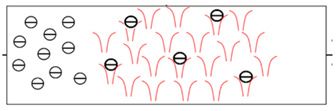
Trap-free SCLC: once all traps are filled, a space charge builds up for $V_{a}>V_{TFL}$ causing a jump in J at $V_{a}=V_{TFL}$.	$n_{inj}>V_{TFL}$, $E_{fn}>E_{t}$ approaches $E_{c\left(min\right)}$. Electric field is no longer constant across the dielectric. SCLC limits further injection of free carriers in the dielectric.	The conduction becomes fully space-charge-limited (Child’s law: $J\propto V^{2}$), with a jump from a low trap-limited value to a high trap-free SCLC.	
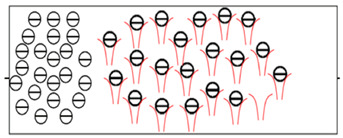

The trap-filled limit (TFL) is the condition for the transition from the trapped J(V) characteristics to the trap-free J(V) characteristics. $V_{TFL}$ is defined as the voltage required to fill the traps, i.e., it is the voltage at which the Fermi level $E_{Fn}$ passes through $E_{t}$. It is possible that space-charge-limited current dominates when a sample is biased in one direction, whereas for the opposite bias, Schottky injection dominates.

##### Ionic Conduction

Under high electric fields and temperatures, ions may travel by jumping over potential barriers from one defect site to another in the direction dictated by the applied electric field. Ions with larger masses will have smaller mobilities. In pyroelectric insulators, ion masses are generally large, while E and T are relatively small; hence, ionic conduction is not very likely.

A schematic energy band diagram is given in Figure A9 for two cases: (a) the absence and (b) the presence of an electric field E. The spacing between ionic sites is d, and the barrier height potential is $q\Phi_{B}$.

The ionic conduction current can be expressed as:

(A36) $$J=J_{0}\exp\left[\frac{q\Phi_{B}}{kT}-\frac{qE_{d}}{2kT}\right],$$

where $J_{0}$ is a proportional constant, $\Phi_{B}$ is the potential barrier height, and d is the spacing between two nearby jumping sites; the other symbols have been defined earlier [113].

##### Grain-Boundary-Limited Conduction

Surface recombination of carriers at the grain boundaries of a polycrystalline material is a function of several parameters, including temperature, grain size, grain orientation, impurity concentration, etc., and is responsible for increased resistivity at the grain boundaries compared to the grain’s bulk. The carriers are scattered by the scattering centers (e.g., charged impurities or defects) residing on the grain boundaries when they are transported from one grain to the next. This mechanism may dominate the conduction properties of the DUT when the grain size is reduced [9,32].

A second mechanism is attributed to the Schottky effect. A potential barrier energy,

(A37) $$q\Phi_{B}=2q^{2}n_{b}^{2}\varepsilon_{r}N,$$

is developed at the grain boundary, where $n_{b}$ is the grain boundary trap density, $\varepsilon_{r}$ is the relative dielectric constant of the polycrystalline material, and N is the local dopant concentration. Therefore, energy bands bend at the grain boundaries, allowing only electrons with a thermal energy $E>q\Phi_{B}$ to cross the barrier (Figure A10). In this case also, the charge transport properties of the polycrystalline film are affected by the electrical properties of the Schottky barrier height at the grain boundaries rather than bulk.

Using impedance spectroscopy [102], grain boundary-limited conduction may be differentiated from the bulk contribution in total conductivity on the account of different relaxation times associated with their response to an AC signal [113].

##### Direct Tunneling

The direct tunneling current component dominates when insulator thickness becomes very thin (<4 nm). In this case, electrons residing on the metal’s Fermi level face the energy barrier of the full oxide thickness. Electrons then will have to tunnel through the trapezoidal potential barrier instead of tunneling through a triangular potential barrier, as was the case for the FN tunneling. The direct tunneling occurs at $V_{ox}<\Phi_{B}$.

The equation governing the current density of the direct tunneling is given by [119]:

(A38) $$J_{DT}\approx \exp\left[\frac{-8\pi kt_{ox}\sqrt{2q}}{3h}{\left(m^{*}\Phi_{B}\right)}^{1/2}\right],$$

Contreras et al. [122] reported on the production of metal-PZT-metal junctions that were sufficiently thin (< 6 nm) for electrons to tunnel through the FE insulator, either directly or through a phonon-assisted process. However, a value much smaller than expected was obtained for the barrier height.

##### Transition from Electrode-Limited to Bulk-Limited Conduction Processes

For highly doped and/or defective insulating film, the conduction process may vary from being electrode-limited at low voltages with essentially thickness-independent and very steep J(V) characteristics to being bulk-limited at high voltages where the current rises less rapidly with increasing voltage, and there is a linear relationship between $\log\left(J\right)$ and $V^{1/2}$. The slope of this curve depends on the nature of the defective structure of the insulator [47]. PT-based insulators generally include donors, traps, and perhaps acceptors that act as compensating centers simultaneously and hence comprise more complicated systems.

#### Appendix A.3.3. Electrical Breakdown

There are two mechanisms generally held responsible for breakdown in the dielectric materials: ionic conduction and thermal breakdown (thermal runaway).

##### Ionic Conduction

Under high electric fields, electrons injected from the cathode by field emission multiply in the dielectric material through ionizing collisions. The breakdown process in FE−PZT arises from the growth of ordered chains of oxygen-deficient material and leads to a dendrite-like conduction pathways through the material, which act as “virtual cathodes”. It can be initiated either at the anode and/or at the cathode of the device. The process of electrical breakdown in PZT arise from dendrite-like conduction pathways through the material which may be initiated at the anodes and/or cathodes with a power-law dependence on thickness as:

(A39) $$E_{B}=Ad^{-\omega },$$

with $\frac{1}{4}<\omega <\frac{1}{2}$, according to the linked defect model by Gerson and Marshall [123]. The dependence on electrode material arises from the work function and the electron affinity of the electrode through the resulting Schottky barrier height. As long as qaE≪kT, where a is the lattice nearest-neighbor oxygen-site hopping distance (approximately a lattice constant), Scott [115] showed that the relation defined as follows:

(A40) $$E_{B}\left(T\right)={\left[\frac{3C_{V}K}{bt_{c}\sigma_{0}}\right]}^{1/2}T\exp\left(\frac{b}{2kT}\right),$$

may be used to estimate the breakdown field for most ferroelectrics. Using (A39), values of approximately 8 MV/cm are predicted to conform well with measurements. On the basis of the electron-lattice scattering mechanism in dielectric materials and the electron behavior in the conduction band of the dielectric, Forlani and Minnaja [124] derived the ionization avalanche probability and evaluated the current density by assuming that electrons are injected through tunneling at the negatively biased contact. The criteria for the onset of breakdown are relevant when the current density reaches some arbitrary value. This treatment reveals a correlation between breakdown field strengths and dielectric thickness. The results derived are applicable to an estimate of the temperature of electrons injected by a tunnel cathode of the metal-dielectric-metal type.

##### Thermal Breakdown (Thermal Runaway)

Power dissipation in a device usually generates heat, which in turn leads to a temperature rise in the device. Since the temperature coefficient of the conductivity is positive, a positive feedback scenario may develop where the device temperature increases without bound. This leads to device instability and eventually to thermal runaway. Dependence of FE oxides on temperature is complicated, generally consisting of a hybrid mechanism comprising an initial electrical phase followed by simple thermal runaway.

### Appendix A.4. Leakage Current Characterization Protocol

Since the time constant associated with an insulator is large, the transient (relaxation) current is generally one of the key artifacts encountered in the measurement of the leakage current, particularly when the insulator is a defective dielectric. As noted in Equation (A4), in a pulsed measurement system, $Q_{R//C}$ depends on the pulse width.

Bouyssou, et al. [42], showed that the resistance degradation (RD) mechanism in PZT is a reversible phenomenon.

Thus, in each measurement step, sufficient time must be allocated to ensure the measurement of leakage current is carried under steady state condition. Based on the transient current data, Chentir et al. [27] established a characterization protocol to minimize the contribution of artifacts to the measurements of leakage current.

A typical representation of the evolution of leakage current for PZT capacitor biased under 5 V at 200 °C is shown in Figure A13. Due to high thermal stress, relaxation and RD occur briefly. After resistance restoration (RR) process is completed, the current remains steady up to 90% of the capacitor’s lifetime.

The leakage current must remain stable during each step of voltage sweep for each temperature. Due to large thermal time constants involved at room temperature, after RR is reached, it may take several weeks to attain steady state. Thus, a pretreatment consisting of applying a constant electric field stress (CES) of 58.33 MV/m at 205 °C is used in order to reach the end of RR state before I(E) measurements, as illustrated in Figure A14. After each prestress, a rather soft CES of about 2 MV/m is kept during temperature sweep to avoid any reversible phenomena.

Once the prestress is accomplished and the test temperature is set, I(E) measurements are performed from 25 MV/m to 83 MV/m at temperatures ranging from 100 °C to 200 °C.

This protocol was applied to determine the influence of PZT thickness on leakage current behavior while excluding perturbations induced by measurement artifacts [38].

Hanrahan [25] utilized a Radiant Technologies Precision Premier II FE testing unit in conjunction with a controlled heating probe station. A schematic of the tested structure is shown in Figure A15. This set up was used to measure bipolar current voltage as well as dielectric hysteresis characteristics at frequencies of 10 kHz<f<50 kHz, with amplitudes of 20–40 V.

The I(V) testing procedure utilized for the assessment of the evolution of the leakage current is depicted in Figure A16. The transient current response at E∼100 MV/m was measured.

Finally, to eliminate the unwanted contribution of polarization (dielectric relaxation) current, researchers [25,38,84,125] used a long hold time to measure leakage current.
